# Experimental benchmarking of quantum state overlap estimation strategies with photonic systems

**DOI:** 10.1038/s41377-025-01755-8

**Published:** 2025-02-12

**Authors:** Hao Zhan, Ben Wang, Minghao Mi, Jie Xie, Liang Xu, Aonan Zhang, Lijian Zhang

**Affiliations:** 1 National Laboratory of Solid State Microstructures, Key Laboratory of Intelligent Optical Sensing and Manipulation, College of Engineering and Applied Sciences, Jiangsu Physical Science Research Center, and Collaborative Innovation Center of Advanced Microstructures, Nanjing University, Nanjing, 210093 Jiangsu China; 2 Department of Physics, Imperial College London, Prince Consort Road, London, SW7 2AZ UK

**Keywords:** Quantum optics, Single photons and quantum effects

## Abstract

Accurately estimating the overlap between quantum states is a fundamental task in quantum information processing. While various strategies using distinct quantum measurements have been proposed for overlap estimation, the lack of experimental benchmarks on estimation precision limits strategy selection in different situations. Here we compare the performance of four practical strategies for overlap estimation, including tomography-tomography, tomography-projection, Schur collective measurement and optical swap test using photonic quantum systems. We encode the quantum states on the polarization and path degrees of freedom of single photons. The corresponding measurements are performed by photon detection on certain modes following single-photon mode transformation or two-photon interference. We further propose an adaptive strategy with optimized precision in full-range overlap estimation. Our results shed new light on extracting the parameter of interest from quantum systems, prompting the design of efficient quantum protocols.

## Introduction

Quantum information processing tasks are normally accomplished by estimating specific parameters encoded in the output states instead of the full knowledge of the states. The estimation of the overlap $c=|\langle \psi |\phi \rangle {|}^{2}$ between two unknown quantum states$\,|\psi \rangle$ and $|\phi \rangle$ is a quintessential example underlying various applications, including relative quantum information^1–5^, entanglement estimation^6–9^, cross-platform verification^10^ and quantum algorithms^11,12^. In particular, state overlap estimation plays a pivotal role in various quantum machine learning algorithms^13,14^, such as quantum neural network training^15–19^, quantum support vector machine^20–25^ and variational quantum learning^26–28^, in which the state overlaps serve as cost functions or kernel functions. However, to date, these applications usually assume the ability of ideal and precise state overlap estimation without considering the precision and imperfections in realistic experiments, which may limit the performance of their actual implementations.

The most intuitive way to estimate the overlap is performing full tomography to reconstruct both quantum states and then calculate the overlap directly. This strategy can be modified by only performing tomography of one state $|\phi \rangle$ and projecting another state $|\psi \rangle$ onto the estimate $|\widetilde{\phi }\rangle$, the success probability of which gives the overlap between the two states. On the other hand, a widely used strategy in many quantum protocols^19,21,29–32^ is a joint measurement on $|\psi \rangle |\phi \rangle$ called the swap test^29^. Swap test has been realized in various quantum systems^33,34^, for example, with the Hong-Ou-Mandel interference (HOMI) of photons^35–37^. There have been efforts to further improve the implementation of the swap test through variational quantum approaches to find shorter-depth algorithms^38^, as well as to estimate both the amplitude and phase of the inner product $\langle \psi |\phi \rangle$^39^. Recently, optimal strategy for overlap estimation has been proposed, achieving ultimate precision among all possible strategies^40^. Yet the optimal strategy involves formidable experimental costs requiring joint measurements on all copies of the quantum states. This gap between theoretical proposals and experimental capabilities, which restricts the practical implementation of many quantum protocols, necessitates benchmarking the attainable precision of overlap estimation strategies feasible with current technologies.

To bridge this gap, here we experimentally evaluate the precision of overlap estimation strategies on the photonic platform. Photonics has emerged as a promising platform for various quantum information applications including quantum machine learning^41,42^, benefited from the development of photonic quantum circuits that have already matured in the implementation of optical neural networks^43–45^. The advantages in high-dimensional encoding and programmable operations using linear optics can be readily generalized to implement quantum-optical neural networks at the single-photon level^15,46^. Developing tailored overlap estimation strategies with optimized precision and efficiency is therefore crucial for the development of photonic quantum machine learning. Moreover, these tailored strategies can be adapted to diverse quantum technology platforms, broadening their application scope.

**Fig. 1:**
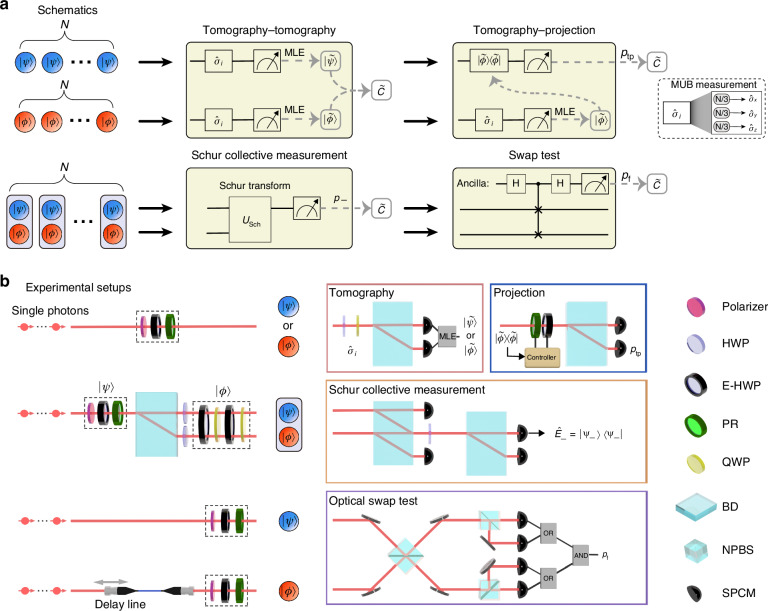
**Schematics and experimental setups of overlap estimation strategies. a** Schematics. Tomography-tomography (TT): Perform quantum state tomography to reconstruct both states using mutually unbiased bases (MUB) and calculate the overlap between $|\widetilde{\psi }\rangle$ and $|\widetilde{\phi }\rangle$. MLE: maximum likelihood estimation. Tomography-projection (TP): $|\phi \rangle$ is reconstructed by tomography, and $|\psi \rangle$ is projected onto the estimate $|\widetilde{\phi }\rangle$. Schur collective measurement (SCM): Schur transform^49^ with computational basis measurements with is applied to project the joint state $|\psi \rangle |\phi \rangle$ on the Schur bases. Swap test: Swap test is conducted on the state $|\psi \rangle |\phi \rangle$, where we realize HOMI as the optical swap test (OST). **b** Experimental setups. Single photons are generated via spontaneous parametric down conversion. Qubits $|\psi \rangle$ and $|\phi \rangle$ are encoded in the polarization DoF of different single photons in TT, TP, and OST. In SCM, the two-qubit state $|\psi \rangle |\phi \rangle$ is encoded in the polarization and path DoF of the single photon. Tomography module (red frame) with wave-plates and a beam displacer performs the measurements of Pauli operators ${\hat{\sigma }}_{i}$ in TT and TP strategies. Projection module (blue frame) registers the successful projections onto $|\widetilde{\phi }\rangle$ to estimate ${p}_{{tp}}$ in TP strategy. SCM module (orange frame) conducts Schur basis projective measurements on $|\psi \rangle |\phi \rangle$. OST module (purple frame) utilizes HOMI to obtain the “fail” probability ${p}_{f}$ of the swap test. (E-)HWP (electronically controlled) half wave-plate, PR liquid crystal phase retarder, QWP quarter wave-plate, BD beam displacer, NPBS non-polarizing beam-splitter, SPCM single photon counting module

In this work, we benchmark four practical overlap estimation strategies suitable for current photonic technologies: tomography-tomography (TT), tomography-projection (TP), Schur collective measurement (SCM), and optical swap test (OST), as illustrated in Fig. 1. By encoding qubit states into various degrees of freedom (DoF) of photons, we experimentally perform the corresponding measurements with linear optics and quantify the estimation precision as a function of the true overlap. Our results demonstrate that different strategies yield varying overlap-dependent precision. By comparing performance across different overlap ranges, we develop an adaptive strategy that combines TP and SCM strategies to achieve optimized precision across the full overlap interval. Furthermore, we quantify the contributions of tomography errors or specific measurement outcome statistics to the final precision for each strategy, elucidating key performance factors. Extending this analysis to higher-dimensional states, we discuss the scaling of the performance of each strategy with respect to state dimension, highlighting the dimension-independence of SCM and OST, and analyzing TT and TP performance under different tomographic measurement schemes including joint and local measurements. These findings provide insights into the analysis of overlap estimation precision and help to design the practical strategy with optimized performance.

## Results

### Overlap estimation strategy performance assessment

Given $N$ pairs of two unknown pure qubit states $|\psi \rangle$ and $|\phi \rangle$, without loss of generality, these states can be expressed as$|\psi \rangle =U|0\rangle$ and $\left|\phi \right\rangle =U\left(\sqrt{c}|0\rangle +{e}^{i\varphi }\sqrt{1-c}|1\rangle \right)$, where $U\in {SU}(2)$ and $c$ represents their overlap. Here, we primarily focus on estimating the overlap between two qubits, with the high-dimensional cases discussed later. An overlap estimation strategy denoted by $s$ involves a general positive operator-valued measure (POVM) $\{{\hat{E}}_{k}^{(s)}\}$ on all copies of the quantum states and the estimation of the overlap as ${\widetilde{c}}_{s}(k)$ based on the outcome $k$. Under a specific choice of $U$ and $\varphi$, the mean squared error of overlap is given by ${v}_{s}(c,\,N|U,\,\varphi )={\sum }_{k}{[{\tilde{c}}_{s}(k)-c]}^{2}{\rm{Tr}}[{\hat{E}}_{k}^{(s)}|\varPhi \rangle \langle \varPhi |]$, where |Φ〉 = (|*ψ*〉|*ϕ*〉)^⨂*N*^. Notably, when the estimator ${\widetilde{c}}_{s}(k)$ is (asymptotically) unbiased, ${v}_{s}(c,N|U,\varphi )$ is equivalent to the variance of ${\widetilde{c}}_{s}(k)$. To compare the average performance of strategy $s$ over all possible quantum states, we consider randomly sampled qubit pairs with a fixed overlap $c$, where $U$ is Haar-distributed in ${SU}(2)$ and $\varphi$ is a uniformly distributed phase between $0$ and $2\pi$. More details can be found in the Supplementary Information (SI). The precision of strategy $s$ can be quantified by the average variance

1 $$\,{v}_{s}\left(c,N\right)=\frac{1}{2\pi }{\int_{U}}{\int_{0}^{2\pi }}{v}_{s}\left(c,{N|U},\varphi \right){dUd}\varphi$$

where ${dU}$ is the Haar measure. We observe that the average variance ${v}_{s}(c,N)$ for each strategy exhibits a scaling behavior of $O(1/N)$. To offset the influence of the copy number $N$, we introduce the scaled average variance $N{v}_{s}(c)$, which only depends on *c* at large $N$, as the performance assessment metric of the overlap estimation strategy *s*. Figure 1a illustrates the four practical strategies for overlap estimation:

#### Tomography-tomography (TT)

Reconstruct the two quantum states through quantum state tomography based on mutually unbiased bases (MUB)^47^, i.e., measuring the Pauli operators $({\hat{\sigma }}_{x},\,{\hat{\sigma }}_{y},\,{\hat{\sigma }}_{z}\,)$ on $N/3$ copies of $|\phi \rangle$ and $|\psi \rangle$ respectively. The estimate states, $|\widetilde{\phi }\rangle$ and $|\widetilde{\psi }\rangle$, yield an overlap estimator as ${\widetilde{c}}_{{tt}}\,=\,{|\langle \widetilde{\psi }|\widetilde{\phi }\rangle |}^{2}$.

#### Tomography-projection (TP)

Reconstruct $|\phi \rangle$ with the same quantum state tomography procedure in TT, project the *N* copies of $|\psi \rangle$ onto the estimate $|\widetilde{\phi }\rangle$ and record the number of successful projections *k*. The overlap estimator is ${\widetilde{c}}_{{tp}}\,=\,k/N$ with the expectation value ${p}_{{tp}}={|\langle \psi |\widetilde{\phi }\rangle |}^{2}$.

#### Schur collective measurement (SCM)

Perform collective measurement on each of the $N$ pairs of the qubits $|\psi \rangle |\phi \rangle$ and record the number of successful projections *k* onto the singlet state $|{\Psi }_{-}\rangle =\,(|01\rangle -\,|10\rangle )/\sqrt{2}$, where the probability of successful projection is ${p}_{-}=(1\,-\,|\langle \psi |\phi \rangle {|}^{2})/2$. The estimator of overlap is given by ${\hat{c}}_{{scm}}=1\,-\,2k/N$.

#### Optical swap test (OST)

Implement a multi- mode HOMI between each of the *N* photon pairs encoding the state $|\psi \rangle |\phi \rangle$ with the pseudo photon-number-resolving detectors (PPNRD). The states will “fail” or “pass” the test and we register $k$ “fail” outcomes out of $N$ measurements (see the SI for definitions). The overlap estimator is ${\hat{c}}_{{ost}}=(1-2k/N)/\varGamma$, where $\varGamma$ is the indistinguishability between the internal modes of two photons (explained later).

We derive the average variances ${v}_{s}(c,N)$ for all the four strategies (see the SI for derivations). The summary of these strategies is presented in Table 1.

**Table 1:** Summary of the four overlap estimation strategies

	TT	TP	SCM	OST
Measurement	${\hat{\sigma }}_{i},\,{\hat{\sigma }}_{i}$	$\,{\hat{\sigma }}_{i},|\widetilde{\phi }\rangle \langle \widetilde{\phi }|$	${\hat{E}}_{-}=|{\Psi }_{-}{{\rangle }}{{\langle }}{\Psi }_{-}|$	HOMI, PPNRD
Estimator $\widetilde{c}$	${|\langle \widetilde{\psi }|\widetilde{\phi }\rangle |}^{2}$	*k*/*N*	1 − 2*k*/*N*	(1 − 2*k*/*N*)/*Γ*
*v*(*c*, *N*)	4*κc*(1 − *c*)/*N*	(2*κ* + 1)*c*(1 − *c*)/*N*	(1 − *c*^2^)/*N*	(3 − *Γc*)(1 − *Γ*^2^ *c*^2^)/2*NΓ*^2^

The average variances $v(c,N)$ are derived in the asymptotic limit ($N\to \infty$). $\{{\hat{\sigma }}_{i}\}$: three Pauli operators. $k$: Measurement outcome statistic in corresponding strategy. $\kappa$: scaled average infidelity in the pure qubit tomography based on MUB (see Materials and methods). *HOMI* Hong-Ou-Mandel interference. *PPNRD* pseudo photon-number-resolving detectors. Γ: indistinguishability between the internal modes of two photons in HOMI

### Photonic implementation of estimation strategies

We experimentally benchmark the aforementioned overlap estimation strategies using photonic systems. The experimental setups, depicted in Fig. 1b, consist of state preparation modules and four measurement modules. Different combination of the state preparation module and the measurement module forms the corresponding strategy.

In the TT and TP strategies, we encode the qubit states $|\psi \rangle$ or $|\phi \rangle$ on the polarization DoF of the heralded single photons generated through the spontaneous parametric down-conversion process. The horizontal $|H\rangle$ and vertical $|V\rangle$ polarizations of the photon represent the computational bases $|0\rangle$ and $|1\rangle$, respectively. In both strategies, measurements of Pauli operators to perform the state tomography are implemented with one half-wave plate, one quarter-wave plate, and one beam displacer (BD). The wave-plates are set into three configurations to implement the three bases in MUB, followed by two single photon counting modules (SPCMs) to register the measurement outcomes. In the TP strategy, a set of electronically-controlled wave-plates enables the projection of $|\psi \rangle$ onto the reconstructed state $|\widetilde{\phi }\rangle$ from the state tomography result of $|\phi \rangle$. The clicks of the corresponding SPCM are registered as the successful projections.

In the SCM strategy, we encode the first qubit $|\psi \rangle$ on the path DoF of a single photon, while the second qubit $|\phi \rangle$ is encoded on the polarization DoF of the photon^48^. The encoding basis is $|00\rangle =|{s}_{0}\rangle |H\rangle$, $|01\rangle =|{s}_{0}\rangle |V\rangle$, $|10\rangle =|{s}_{1}\rangle |H\rangle$, $|11\rangle =|{s}_{1}\rangle |V\rangle$, with ${s}_{0}$ (down) and ${s}_{1}$ (up) denoting two path modes of the photon. The POVM in the SCM strategy involves four projectors which realize the projections on the Schur bases^49^: ${\hat{E}}_{1}\,=|00\rangle \langle 00|$, ${\hat{E}}_{2}\,=\,|11\rangle \langle 11|$, ${\hat{E}}_{+}\,=\,|{\Psi }_{+}\rangle \langle {\Psi }_{+}|$, ${\hat{E}}_{-}=|{\Psi }_{-}\rangle \langle {\Psi }_{-}|$ with $|{\Psi }_{+}\rangle =\,(|01\rangle +\,|10\rangle )/\sqrt{2}$ and $|{\Psi }_{-}\rangle =(|01\rangle -|10\rangle )/\sqrt{2}$. It is noteworthy that we only need the outcome probability of ${\hat{E}}_{-}$ while the other three are need for the normalization condition (see Materials and methods). To realize these projectors, as illustrated at the SCM module in Fig. 1b, the first BD splits the horizontal and vertical polarization modes of the two path modes. The horizontal polarization of the ${s}_{0}$ path and the vertical polarization of the ${s}_{1}$ path are detected by two single-photon counting modules (SPCMs), which realize projectors ${\hat{E}}_{1}$ and ${\hat{E}}_{2}$. The half-wave plate and another BD, followed by two SPCMs, implement the projectors ${\hat{E}}_{+}$ and ${\hat{E}}_{-}$ (see the SI for the details).

In the OST strategy, we encode $|\psi \rangle$ and $|\phi \rangle$ on the polarization DOF of two different photons, where we regard other modes of the photon as internal modes. The OST is implemented via a multi-mode HOMI^36^ for each pair of the two photons at a balanced non-polarizing beam splitter (NPBS). After the interference, a combination of a balanced NPBS followed by two SPCMs is placed at each output port of the NPBS to function as a PPNRD, the “pass” outcome of the OST corresponds to the event that both photons exit the same port of the first NPBS, while the “fail” outcome corresponds to the coincidence events that two photons are detected in different output ports of the first NPBS. Due to experimental imperfections, the two photons from the SPDC source exhibit reduced indistinguishability even when they encode the same qubit state, due to the mismatch of their internal modes, primarily the spectral mode^50^. We quantify this indistinguishability as $\varGamma =0.965$, which is estimated by the maximum visibility of HOMI. In the SI, we derive the unbiased overlap estimator and its associated variance in the presence of *Γ*. Our analysis confirms the feasibility of performing overlap estimation using the OST strategy even in the presence of practical experimental imperfections, though the precision is reduced.

### Overlap-dependent precision of strategies

To provide a fair comparison for different overlap estimation strategies, we employ the same number of quantum states for each strategy. Specifically, we perform the experiments for 11 overlap values equally spaced in the range $[0,\,1]$. For each overlap *c*, we uniformly and randomly sample *M* = 100 qubit pairs $|{\psi }_{m}(c)\rangle$ and $|{\phi }_{m}(c)\rangle$, with $|\langle {\psi }_{m}(c)|{\phi }_{m}(c)\rangle {|}^{2}=\,c$ and $m\,\in \,\{1,\,2,...,M\,\}$. For each qubit pair, we collect the measurement outcomes for $N\,=\,900$ copies to obtain an estimated overlap ${\widetilde{c}}_{m}$. This data collection and estimation process is repeated $n=20$ times to give the estimated variance ${\widetilde{v}}_{m}(c)$. By averaging over $M$ sampled qubit pairs, which is approximately equivalent to integrate with ${SU}(2)$ in Eq. (1), we obtain the measured average variance for the strategy. To further determine the uncertainties of the estimated variance, $R\,=\,10$ independent experiments are conducted, producing a total data set $\{\{\{{\widetilde{c}}_{m}^{j,r}{\}}_{j=1}^{n}{\}}_{m=1}^{M}{\}}_{r=1}^{R}$ of $100\times 20\times 10$ estimations for each overlap value of a strategy (see Materials and methods for details of data processing).

**Fig. 2:**
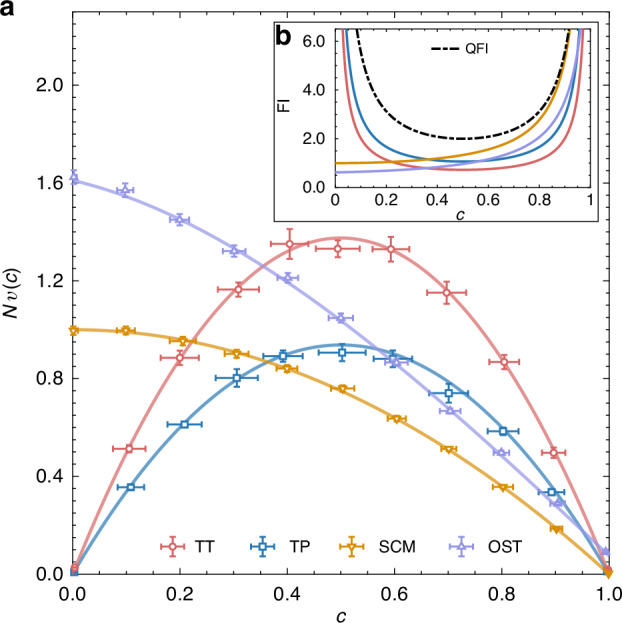
**Experimentally measured scaled average variances** ${\boldsymbol{Nv}}({\boldsymbol{c}})$ **and the corresponding Fisher information vs the value of overlap** ${\boldsymbol{c}}$**. a** Experimentally determined (markers) and theoretical (solid lines) ${Nv}(c)$ for four overlap estimation strategies with the copy number $N\,=\,900$. Vertical error bars represent the uncertainties of ${Nv}(c)$ over $10$ runs of the experiments. Horizontal error bars denote the standard deviation of the exact overlap values for different qubit pairs generated in the experiments (see “Materials and methods”). **b** Normalized Fisher information (FI) per state pair at large $N$ for each strategy. The black dashed line indicates the quantum Fisher information (QFI)

Figure 2a shows the experimentally measured average variances scaled by the number of copies ${Nv}(c)$ for the four strategies. The results exhibit a clear overlap-dependent performance for all strategies, aligning well with theoretical predictions. The average variances of the two local measurement strategies, TT and TP, show symmetric behaviors in the entire overlap range. Both strategies achieve higher precision near $c=0$ and $c=1$ but lower precision for intermediate overlaps around $0.5$. TP outperforms TT for all values of $c$, due to the fact that the tailored projective measurement in TP provides more overlap information compared with tomography. In contrast, the two joint measurement strategies, SCM and OST, exhibit monotonic behaviors, i.e., they achieve lower precision for small *c* but higher precision for large *c* in comparison with TT and TP. Notably, SCM and OST are two different experimental realizations of the destructive swap test^36^. Therefore, they are expected to exhibit the same performance. Yet, the actual performance of OST in our experiment worse than that of SCM. We attribute this performance gap to experimental imperfections in the OST setup, detailed further in the SI. The first factor is the use of PPNRDs, which introduce extra photon loss and alter the outcome probability distribution, which is especially detrimental for small overlaps. The second factor is the limited HOMI visibility of two photons, leading to a constant reduction in precision over the whole range of overlaps. These two factors together contribute to the reduced OST precision observed in the experiment. Furthermore, we evaluate the overlap estimator in each strategy by calculating the normalized Fisher information (FI) per state pair, as shown in Fig. 2b. The Cramér-Rao bound, defined as the inverse of the FI, provides a lower bound on the variance of any unbiased estimator for a parameter^51^. In the large *N* limit, the normalized FI is equivalent to the inverse of corresponding ${Nv}(c)$ for each strategy, indicating that their overlap estimators saturate the Cramér-Rao bound. Notably, when considering large overlaps, the FI for the SCM strategy converges towards the quantum Fisher information^40,52^, which is the upper bound of FI for all possible measurement strategies, indicating the SCM strategy achieves ultimate precision for large overlaps. It reveals that the collective measurements involving more copies of states cannot outperform the SCM only involving a pair of states when the overlap approaches unity.

**Fig. 3:**
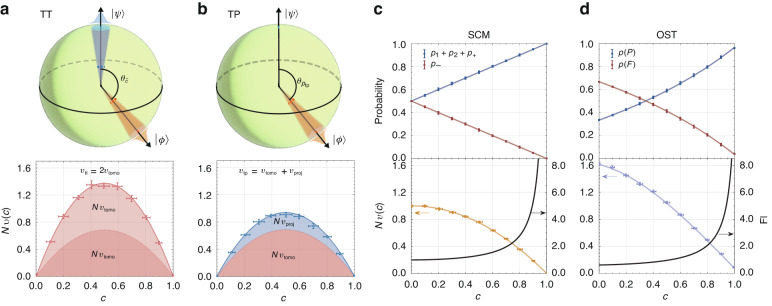
**Detailed analysis of the contributions to the average variance of different overlap estimation strategies**. Representations of the tomography error distributions in the Bloch sphere and their overlap-dependent contributions to the average variance in (**a**) TT and (**b**) TP strategies. Projection error accounts for the remaining portion of the average variance in TP strategy. Outcome probabilities, scaled average variances *Nv*(*c*) and normalized Fisher information (FI) are presented in (**c**) for SCM, and (**d**) for OST. In (**c**) “${p}_{1}+{p}_{2}+{p}_{+}$” represents the sum of the probabilities of the first three projectors, while ${p}_{-}$ corresponds to the probability of the last projector ${\hat{E}}_{-}$. In (**d)** $p(P)$ and $p(F)$ indicate the raw detection probability of “pass” and “fail” outcomes under the PPNRD setup, respectively. Markers and lines denote experimental and theoretical results, respectively. Experimentally measured probabilities are estimated from total datasets comprising $\mathrm{180,000}$ measurements per qubit pair, and the error bars represent the standard deviation of the probabilities obtained from different qubit pairs

In fact, the distinct behaviors for the four estimation strategies arise from the characteristics of their measurements and estimators. Both separable measurement strategies can be separated into two separable measurement and estimation processes. Therefore, the average variance can be decomposed into the contribution either from two state tomography processes (TT), or one state tomography and the projective measurement (TP). The contribution of each part is given as (see the SI for derivations)

2 $$\,{v}_{{tomo}}\left(c,N\,\right)=\frac{2\kappa c\left(1\,-\,c\right)}{N}$$

3 $$\,{v}_{{proj}}\left(c,N\,\right)=\frac{c\left(1\,-\,c\right)}{N}$$

respectively, where $\kappa$ denotes the scaled average infidelity in the tomography process (see Materials and methods). It is noteworthy that the inherent error in tomography process is independent of the overlap $c$, whereas its contribution to the overlap estimation variance is overlap-dependent. Different combinations of the variances in Eqs. (2 and 3) lead to the overall average variance of the two strategies, as illustrated in Fig. 3a, b. For the joint measurement strategies, the overlap is estimated directly from outcomes of the joint measurements on the qubit pair. The average variance is directly related to the Fisher information from the probability distribution of measurement outcomes, as shown in Fig. 3c, d.

The aforementioned variance results allow us to determine the number of copies of states required to achieve a desired precision in overlap estimation. Applying Chebyshev’s inequality, an overlap can be estimated with an error bounded by $|{\widetilde{c}}_{s}-c|\le \varepsilon$ and ensure a probability exceeding $1-\eta$ by using approximately $N \sim {f}_{s}(c)/\eta {\varepsilon }^{2}$ copies of states (see the SI for details). Here, ${f}_{s}(c)$ denotes the scaled average variance for strategy $s$. Consequently, the overlap estimation error $\varepsilon$ scales as $O(1/\sqrt{N}\,)$. The fact that different strategies exhibit same scaling behavior for $N$, justifies the efforts on developing practical strategies to reduce the scaled average variance.

### Adaptive overlap estimation strategy

**Fig. 4:**
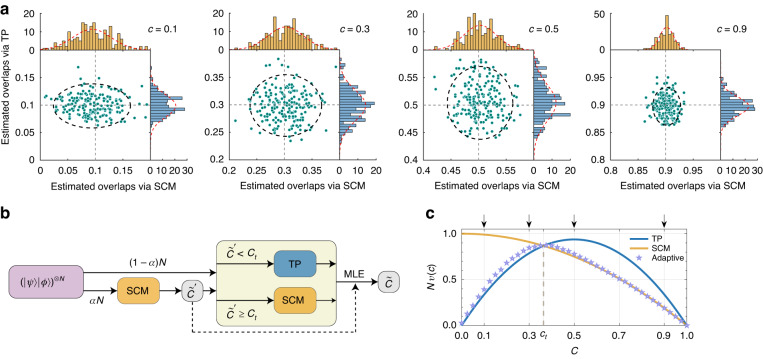
**Adaptive overlap estimation strategy based on TP and SCM. a** Comparison between the TP strategy and the SCM strategy for four exemplary overlaps indicated in (**c**). Markers represent the experimentally estimated overlaps obtained using both strategies with $N\,=900$ copies of the sampled qubit pair. The histograms along the horizontal (SCM) and vertical (TP) axes depict the marginal distributions of $200$ estimated overlaps. The red dashed lines in the histograms represent the corresponding Gaussian function fittings. The black dashed line ellipse in each panel, with principal axes proportional to the standard deviations of the marginal distributions, demonstrates the relative precision of the two strategies. **b** Schematic of the two-step adaptive strategy for overlap estimation. In the first step, an initial estimation of overlap ${\widetilde{c}}^{{\prime} }$ is obtained using SCM with $\alpha N$ ($0 < \alpha < 1$) copies of $\left|\psi \right\rangle |\phi \rangle$. Based on the comparison between ${\widetilde{c}}^{{\prime} }$ and ${c}_{t}$, one of the TP and SCM strategies is chosen for the second step with $(1-\alpha )N$ copies of $|\psi \rangle |\phi \rangle$. The final estimation $\widetilde{c}$ is obtained through the maximum likelihood estimation (MLE) using the two-step overlap estimation results. **c** Simulation results of the adaptive strategy. $N=900$ and $\alpha =1/30$ are adopted in the simulation

From the above experiments we can conclude that the optimal strategy among the four investigated ones varies with overlap value. As a detailed comparison, Fig. 4a compares the experimentally estimated overlaps $\widetilde{c}$ with TP and SCM for different *c*. From this comparison and the average variances in Fig. 2a, we identify that the average variances of TP and SCM intersect at overlap ${c}_{t}=\,4/11$. In other words, the most efficient strategy among the four strategies is TP when the overlap $c < {c}_{t}$ and SCM when$\,c\ge {c}_{t}$. Leveraging this observation, we propose a two-step adaptive strategy that combines TP and SCM strategies, as illustrated in Fig. 4b. Our simulation results ${Nv}(c)$ for the adaptive strategy are shown in Fig. 4c. In the first step of the adaptive strategy, the SCM strategy is employed on $\alpha N$ pairs of states to get a rough estimation ${\widetilde{c}}^{{\prime} }$, which then determines the strategy used in the second step. Notably, the copies of states used in the first step are not used in tomography process when the second step involves the TP strategy. Although the estimation variance of the adaptive strategy slightly deviates from that of the TP strategy when $c < {c}_{t}$ due to the resource consumption in the first step, our adaptive strategy still achieves nearly optimal estimation precision across the full range of overlap values compared with the four static strategies.

### Overlap estimation of high-dimensional states

The preceding analysis of the average variance of overlap estimation strategies can be generalized to high-dimensional and multi-qubit quantum states. Consider estimating the overlap between two $d$-dimensional states in a sufficient-copy scenario ($N\,\gg \,d$). For separable measurement strategies, the contribution of tomography errors to the average variance in this high-dimensional case is (see the SI for derivations)

4 $$\,{v}_{{tomo}}\left(c,N\,\right)=\frac{2\kappa c\left(1\,-\,c\right)}{(d-1)N}$$

where $\kappa$ is the scaled average infidelity of the underlying pure state tomography approach. For $d\,=\,2$, this recovers the result in Eq. (2). The factor $1/(d-1)$ in Eq. (4) arises because, in high-dimensional state spaces, the ratio of the tomography errors projected onto the subspace spanned by $|\psi \rangle$ and $|\phi \rangle$ diminishes, thereby reducing their impact on overlap estimation. The scaling of $\kappa$ with respect to $d$ depends on the specific tomography measurements employed. When joint measurements across all copies are allowed, or when arbitrary independent measurements on each copy are permitted, $\kappa$ scales as $O(d)$^53–55^, resulting in a dimension-independent average variance of $O(c(1-c)/N)$ for both TT and TP strategies. This dimension-independence holds specifically under the sufficient-copy condition $(N\gg d)$. When the high-dimensional states are $n$-qubit states $(d={2}^{n})$, restricting tomography to local, single-qubit measurements leads to $\kappa$ scaling as $O({d}^{2}\mathrm{log}d)\,=\,O({4}^{n}n)$^56^, and a dimension-dependent average variance of $O({4}^{n}nc(1\,-\,c)/N\,)$ for TT and TP.

For two joint measurement strategies, both SCM and OST can be extended to higher dimensions while maintaining precision independent of $d$ (see the SI for details). In the sufficient-copy scenario, allowing joint measurements for tomography yields comparable performance across all four strategies. However, SCM and OST significantly outperform TT and TP for multi-qubit states when the latter are restricted to local measurements on each qubit for tomography.

In the limited-copy scenario ($N\sim d$), tomography yields highly inaccurate estimations due to information incompleteness and substantial statistical errors. Therefore, the errors of TT and TP strategies deviate significantly from the average variances derived in the sufficient-copy scenario, as the bias becomes non-negligible. Both ${v}_{{tt}}$ and ${v}_{{tp}}$ are then dominated by a constant error scale as $O(1)$ (see the SI for derivations). This problem is exacerbated by increasing qubit number $n$, leading to exponential growth in $d$ and rendering quantum state tomography infeasible. In these situations, SCM and OST offer a significant advantage due to their inherent dimension independence.

## Discussion

In this work, we present a comprehensive investigation of four representative strategies for estimating the overlap of two unknown quantum states using a photonic setup. We compare the performance in terms of the average estimation variance of the separable measurement strategies including TT and TP with that of the joint measurements strategies including SCM and OST. Our experimental results demonstrate the superior performance of the TP strategy over the TT strategy for all overlap values considered. Moreover, although in principle the OST strategy matches the performance of the SCM strategy, it exhibits poorer performance in the presence of experimental imperfections when compared to the SCM strategy, which indicates that high-dimension encoded single-photon systems are more robust against experimental errors. These results reveal that the optimal strategy among the four varies depends on the overlap values. To approach the optimal performance across the full range of overlaps, we further design an adaptive strategy combining TP and SCM strategies. Our experiments with single qubits show that separable measurements involving tomography achieve precision comparable to joint measurements performed on pairs of states. Yet, for quantum states with higher dimensions and multiple qubits, theoretical analysis reveals that SCM and OST benefit from dimension-independence, providing a significant advantage, whereas TT and TP become highly dimension-dependent when the number of copies is limited or only single-qubit measurements are available for tomography. By elucidating the overlap-dependent precision with practical setups, our work provides new insights into designing measurement strategies for extracting parameters of interest from quantum states, a vital task in quantum information applications^1–10,17–31^.

Several avenues exist for future research to enhance the strategies presented here. The separable measurement strategies, TT and TP, can benefit from adaptive quantum state tomography techniques^57–59^. The SCM strategy can be further improved by incorporating collective measurements involving more than one pair of states, surpassing the performance of the ideal swap test, as discussed in^40^. Moreover, the SCM and OST strategies can be generalized and applied to higher dimensional or multi-qubit quantum systems^37^^,60^. In practice, the efficiency of all strategies can be improved by utilizing faster optical systems^61^^,62^.

Our work provides an example of striking a balance between optimized performance and experimental complexity, aiming to minimize the gap between theoretical proposals and experimental attainable performance in overlap estimation strategies. Given the prevalence of overlap estimation in quantum machine learning algorithms^17–31^, the optimized estimation strategies can find immediate applications in quantum algorithms involving readout of state overlaps as the cost function or quantum kernel function^18–23^. State overlaps quantify the similarity between data points mapped into the quantum feature space in quantum kernel methods, which have wide-ranging applications from data classification to training quantum models^62–68^. We anticipate that the strategies explored here, along with the understanding of their corresponding precision, can be applied to construct quantum kernels, learn quantum systems and train quantum neural networks, resulting in improved training efficiency and overall performance.

## Materials and methods

### Precision of separable measurement strategies

In TT and TP strategies, reconstructing the qubit states relies on the tomography based on MUB measurements with the prior knowledge that the state is pure. The tomography fidelity can be quantified by $F\,=\,{|\langle \psi |\widetilde{\psi }\rangle |}^{2}$, defined as the overlap between the true state $|\psi \rangle$ and the reconstructed state $|\widetilde{\psi }\rangle$. We consider the average fidelity $\bar{F}$, averaged over the distribution of the reconstructed state and the unitary $U$ where $|\psi \rangle =\,U|0\rangle$. At the asymptotic limit (the number of copies $N\to \infty$), the average fidelity is derived as $\bar{F}=1-\kappa /N$, and $\kappa =N\left(1-\bar{F}\right)$ is defined as the scaled average infidelity, with the analytical value $\kappa =11/8$ for MUB measurements. Through the error analysis in the tomography process, we can represent the reconstructed states $|\widetilde{{\psi }}\rangle$ as

5 $$|\widetilde{{\psi }}{{\rangle }}=\cos \chi |{\psi }{{\rangle }}+\sin \chi {e}^{i\zeta }|{{\psi }}_{\perp }{{\rangle }}$$

where |*ψ*_⊥_〉 = *U* |1〉, *χ* and *ζ* are two error parameters introduced by the tomography. Furthermore, we derive the average values for the functions of *χ* and *ζ* as: $\overline{\langle {(\sin \chi \cos \zeta )}^{2}\rangle }\approx \overline{\langle {(\sin \chi \sin \zeta )}^{2}\rangle }\approx \overline{\langle {\chi }^{2}\rangle }/2\approx 2\kappa /N$, where 〈·〉 and the overline denote the average over the conditional probability distribution $p(\chi ,\zeta |U\,)$ and the unitary *U*, respectively.

In the TT strategy, the other reconstructed state $|\widetilde{\phi }\rangle$ has a similar form to Eq. (5), and the variance for TT can be expressed with the error parameters $\chi$ and $\zeta$. The average variance can then be shown as

6 $${v}_{{tt}}\left(c,N\right)=2\overline{\left\langle {\chi }^{2}\right\rangle }c(1-c)+O\left(\frac{1}{{N}^{2}}\right)\approx \frac{4\kappa c\left(1\,-\,c\right)}{N}$$

here we keep the leading term.

In the TP strategy, we only need to consider the tomography error of the one state $|\phi \rangle$, but together with an additional error introduced by the projection procedure. The successful projection probability ${p}_{{tp}}\,=\,{|\langle \widetilde{\phi }|\psi \rangle |}^{2}$ can be shown as the function of parameters $\chi$ and $\zeta$ in $|\widetilde{\phi }\rangle$. The average variance for TP strategy is derived as ${v}_{{tp}}\left(c,N\right)=\overline{\langle {({p}_{{tp}}-c)}^{2}\rangle }+\overline{\langle {p}_{{tp}}(1-{p}_{{tp}})/N\rangle }$, where $\overline{\langle {({p}_{{tp}}-c)}^{2}\rangle }$ implies the tomography error and $\overline{\langle {p}_{{tp}}(1-{p}_{{tp}})/N\rangle }$ denotes the projection error. The final result for ${v}_{{tp}}$ is given by

7 $$\begin{array}{c}{v}_{{tp}}\left(c,N\right)=\overline{\left\langle {\chi }^{2}\right\rangle }c\left(1-c\right)+\frac{c\left(1-c\right)}{N}+O\left(\frac{1}{{N}^{2}}\right)\\ \approx \frac{\left(2\kappa +1\right)c\left(1\,-\,c\right)}{N}\end{array}$$

Generalizing to $d$-dimensional states, the average variances for TT and TP can be shown as

8 $$\begin{array}{c}{v}_{{tt}}\left(c,N\right)\approx \frac{4\kappa c\left(1\,-\,c\right)}{(d-1)N}\\ {v}_{{tp}}\left(c,N\right)\approx \left(\frac{2\kappa }{d-1}+1\right)\frac{c\left(1\,-\,c\right)}{N}\end{array}$$

where *d* denotes the dimension of a single-copy state. The scaled average infidelity $\kappa$ for high-dimensional state tomography depends on $d$ and varies with the tomography approach. In the SI, we provide detailed derivations of the average variances and their high-dimensional generalizations, and also demonstrates that the estimators used in TT and TP are asymptotically unbiased.

### Precision of joint measurement strategies

In a joint measurement strategy, the overlap information is extracted directly by performing a POVM $\{{\hat{E}}_{i}\}$ on the joint state $|{\Phi }_{0}\rangle =\,|\psi \rangle |\phi \rangle$, with each element ${\hat{E}}_{i}$ associated with a measurement outcome $i$. According to Born rule, the probability of obtaining the outcome $i$ is ${p}_{i}={\rm{Tr}}\left({\hat{E}}_{i}\,|{\Phi }_{0}\rangle \langle {\Phi }_{0}|\right)$, which depends on the overlap $c$. Noting that the joint measurement is static, the precision of the overlap estimation is bounded by the Fisher information (FI) derived from the probability distribution as $I(c)={\sum }_{i}\,{p}_{i}{(d\log {p}_{i}/{dc})}^{2}$. In the SCM strategy, the measurements are described by four projectors$\{{\hat{E}}_{1},\,{\hat{E}}_{2},{\hat{E}}_{+},\,{\hat{E}}_{-}\}$, and the corresponding probability distribution is given by

9 $${p}_{1}+{p}_{2}+{p}_{+}=\frac{1+c}{2},\,{p}_{-}=\frac{1-c}{2}$$

where ${p}_{i}=\langle {\Phi }_{0}{|}{\hat{E}}_{i}|{\Phi }_{0}\rangle$. By combining the first three outcomes into one, we obtain binary outcomes where the probabilities solely rely on the overlap $c$. The FI per state pair is given by ${I}_{{scm}}\,=\,1/(1-{c}^{2})$. The overlap estimator ${\widetilde{c}}_{{scm}}=1-2k/N$, where $k$ is the number of occurrences of outcome ${\hat{E}}_{-}$ in $N$ measurements, saturates the Cramér-Rao bound with the variance

10 $${v}_{{scm}}\left(c,N\right)=\frac{1-{c}^{2}}{N}$$

In the OST strategy, the ideal OST yields a binary outcome of either “pass” or “fail” with the probability of “fail” outcome given by ${p}_{f}=(1-c)/2$. However, due to experimental imperfections, the outcome probability distribution deviates from the ideal case. In our experiments, with the internal mode indistinguishability $\varGamma$ between two photons in HOMI and the PPNRD setup, the outcome probability distribution is given by

11 $$p(P)=\frac{1+\varGamma c}{3-\varGamma c},\,p(F)=\frac{2-2\varGamma c}{3-\varGamma c}$$

where $p(P)$ and $p(F)$ denote the probabilities that the PPNRD response the “pass” and “fail” outcomes, respectively. It is worth noting that the PPNRD introduces photon loss, which must be take into account in precision comparison. On average, for $N$ state pairs, only ${N}^{{\prime} }=(3-\varGamma c)N/4$ events are detected. To ensure a fair comparison, we calculate that effective FI per state pair as ${I}_{{ost}}^{e}=\,2{\varGamma }^{2}/(3-\varGamma c)(1-{\varGamma }^{2}{c}^{2})$ to bound the precision of OST (see the SI for detailed derivation). Using the estimator ${\widetilde{c}}_{{ost}}\,=\,(1-2{k}_{f}/N)/\varGamma$, the variance for the OST strategy is given by

12 $$\,{v}_{{ost}}\left(c,N\right)=\frac{(3-\varGamma c)(1-{\varGamma }^{2}{c}^{2})}{2N{\varGamma }^{2}}$$

In these two joint measurement strategies, the outcome probabilities depend solely on the overlap between the two states, rather than the specific states themselves. Therefore, the variance mentioned above is equal to the average variance in SCM and OST.

### Photon source

Frequency-doubled light pulses ($\sim 150$ fs duration, $415$ nm central wavelength) originating from a Ti: Sapphire laser (76 MHz repetition rate; Coherent Mira-HP) pump a beta barium borate ($\beta$-BBO) crystal phase-matched for type-II beamlike spontaneous parametric down conversion (SPDC) to produce degenerate photon pairs ($830$ nm central wavelength). The photon pairs undergo spectral filtering with 3 nm full-width at half-maximum and are collected into single-mode fibers. The pump power is set to $\sim 100$ mW to ensure a low probability of emitting two-photon pairs. In TT, TP, and SCM experiments, one of the photon pair is detected by a SPCM (Excelitas Technologies), while the other serves as a heralded single photon. In the OST experiment, both photons undergo HOMI. Despite the presence of systemic errors and interference drift, an average maximum HOMI visibility of $0.965\pm 0.008$ is observed.

### State preparation

In the TT, TP, and OST strategy experiments, a combination of an electronically controlled half wave-plate (E-HWP) and a liquid crystal phase retarder (LCPR, Thorlabs, LCC1113-B), prepares the horizontal polarized photon to the state

13 $$|\psi \rangle \,{\rm{or}}\,|\phi \rangle =\,\cos 2\theta |H\rangle +{e}^{i\alpha }\,\sin 2\theta |V\rangle$$

where *θ* and *α* denote the E-HWP angle and the relative phase between two polarizations added by the LCPR, respectively. In the SCM strategy experiment, we firstly encode $|\psi \rangle$ on the polarization DoF of the single photon and use a BD and HWPs to transfer the polarization-encoded qubit to a path-encoded qubit. The second qubit $|\phi \rangle$ is then encoded on the polarization DoF of the photon through a E-HWP and a QHQ (QWP-HWP-QWP) wave-plate group, resulting in a two-qubit joint state

14 $$\left|\psi \right\rangle \otimes \left|\phi \right\rangle =\,\left(\cos 2{\theta }_{1}\left|{s}_{0}\right\rangle +\,{e}^{i{\alpha }_{1}}\sin 2{\theta }_{1}\left|{s}_{1}\right\rangle \right) \,\otimes \left(\cos 2{\theta }_{2}\,|H{{\rangle }}+\,{e}^{i{\alpha }_{2}}\sin 2{\theta }_{2}\,|V{{\rangle }}\right)$$

Here, *θ*_1_ and *α*_1_ denote the E-HWP angle and the relative phase from the LCPR used to prepare $|\psi \rangle$, and ${\theta }_{2}$ and ${\alpha }_{2}$ denote the E-HWP angle and the relative phase from the QHQ group used to prepare |*ϕ*〉.

### Data processing and uncertainty quantification

For each chosen overlap $c$ in our experiments, we have a total data set of estimated overlaps $\{\{\{{\widetilde{c}}_{m}^{j,r}{\}}_{j=1}^{n}{\}}_{m=1}^{M}{\}}_{r=1}^{R}$. Here, $R$ groups of data are collected by repetitive runs of the experiments for the TT, OST, and SCM strategies, while in the TP strategy, the data is generated using the Bootstrap method from a single group to reduce data acquisition time. To obtain the estimated average variances ${\left\{{\widetilde{v}}^{r}\right\}}_{r=1}^{R}$, we process the overlap data as

15 $${\widetilde{v}}^{r}=\frac{1}{M}\mathop{\sum }\limits_{m=1}^{M}{\widetilde{v}}_{m}^{r},\,{\widetilde{v}}_{m}^{r}=\frac{1}{n-1}\mathop{\sum }\limits_{j=1}^{n}{\left({\widetilde{c}}_{m}^{j,r}-\frac{1}{n}\mathop{\sum }\limits_{j=1}^{n}{\widetilde{c}}_{m}^{j,r}\right)}^{2}$$

The mean and the standard deviation for the average variance are then calculated as

16 $$\,\widetilde{v}=\frac{1}{R}\mathop{\sum }\limits_{r=1}^{R}{\widetilde{v}}^{r},\delta \widetilde{v}=\sqrt{\frac{{\sum }_{r}{\left({\widetilde{v}}^{r}-\widetilde{v}\right)}^{2}}{R-1}}$$

Scaling the results by the copy number $N$, $N\widetilde{v}$ and $N\delta \widetilde{v}$ correspond to the scaled average variance ${Nv}(c)$ and the vertical uncertainty in Fig. 2a. Considering the systematic errors in state preparation and measurements, the exact overlaps being measured, between different qubit pairs in state preparation, may deviate from the target overlap $c$. To quantify this uncertainty, we estimate the exact overlaps from the data for the same pairs of states in large number of copies to obtain the exact overlap data set $\{{\bar{c}}_{m}{\}}_{m=1}^{M}$, where ${\bar{c}}_{m}={\sum }_{j,r}{\widetilde{c}}_{m}^{j,r}/nR$. The average exact overlap and corresponding standard deviation are given by

17 $$\bar{c}=\frac{1}{R}\mathop{\sum }\limits_{m=1}^{M}{\bar{c}}_{m},\,\delta \bar{c}=\sqrt{\frac{{\sum }_{m}{\left({\bar{c}}_{m}-\bar{c}\right)}^{2}}{M-1}}$$

Here, $\bar{c}$ and $\delta \bar{c}$ indicate the overlap $c$ (markers) and the corresponding uncertainty (horizontal error bars) in Fig. 2a.

## Supplementary information

Supplementary Information for ``Experimental benchmarking of quantum state overlap estimation strategies with photonic systems''

Source files of figures in the main text

Dataset all

## Data Availability

All data needed to evaluate the conclusions in the paper are present in the paper and/or the Supplementary Information.
