# Supplementary material for: Experimental benchmarking of quantum state overlap estimation strategies with photonic systems
Source: Light Sci Appl. 2025 Feb 12;14:83. doi: 10.1038/s41377-025-01755-8 (PMC11814415; doi:10.1038/s41377-025-01755-8)
Supplement: Supplementary file 1 — Supplementary Information for ``Experimental benchmarking of quantum state overlap estimation strategies with photonic systems'' [file 41377_2025_1755_MOESM1_ESM.pdf]

Supplementary Information for "Experimental benchmarking of quantum state overlap estimation strategies with photonic systems"

Hao Zhan,[1] Ben Wang,[1] Minghao Mi,[1] Jie Xie,[1] Liang Xu,[1] Aonan Zhang,[1,2,*] and Lijian Zhang[1,†]

[1] *National Laboratory of Solid State Microstructures, Key Laboratory of Intelligent Optical Sensing and Manipulation, College of Engineering and Applied Sciences, Jiangsu Physical Science Research Center, and Collaborative Innovation Center of Advanced Microstructures, Nanjing University, Nanjing 210093, China*

[2] *Department of Physics, Imperial College London, Prince Consort Road, London SW7 2AZ, United Kingdom*

CONTENTS

I. Overlap estimation strategy performance with qubit pair sampling 2

II. Tomography-Tomography and Tomography-Projection 3
A. Average infidelity of pure state tomography based on MUB 3
B. Tomography error analysis 5
C. Tomography-Tomography strategy precision 8
D. Tomography-Projection strategy precision 9
E. Numerical results of average variance for TT and TP strategies 10
F. TT and TP strategies for high-dimensional quantum states 11
G. TT and TP strategies performance with high-dimensional state tomography 14

III. Optical swap test with experimental imperfections 16
A. Ideal optical swap test 16
B. OST with partially distinguishable photons and non-balanced beam-splitter 17
C. OST with pseudo photon-number-resolving detectors 19
D. OST strategy precision with our experimental setup 20

IV. Experimental details 21

V. Supplementary Results 23

References 26

* Previous email: a.zhang@imperial.ac.uk; Current email: aonan.zhang@physics.ox.ac.uk
† lijian.zhang@nju.edu.cn

I. OVERLAP ESTIMATION STRATEGY PERFORMANCE WITH QUBIT PAIR SAMPLING

In a general overlap estimation scenario, we consider N copies of $|\psi\rangle$ and M copies of $|\phi\rangle$, which can be represented as $|\psi\rangle = U|0\rangle$ and $|\phi\rangle = UW|0\rangle$, with U, W in the special unitary group $SU(d)$. The overlap between two states is given by $c = |\langle\psi|\phi\rangle|^2 = |\langle 0|W|0\rangle|^2$ and the overlap information is solely contained in W. An overlap estimation strategy involves measuring all states using a general measurement $\{E_k\}$ with outcome k and outputting an estimation $\tilde{c}(k)$ of the overlap. We assess the performance of an overlap estimation strategy using the local approach mentioned in [1], assuming that the overlap c is fixed. For a fixed overlap c, the set of possible unitary W is given by $w = \{W \in SU(d) : |\langle 0| W |0\rangle|^2 = c\}$. We quantify the estimation precision by computing the average of the square error over all states with fixed overlap c and over all outcomes:

$$v(c) = \int_{SU(d)} \int_w dU dW \sum_k [\tilde{c}(k) - c]^2 \mathrm{Tr}\left[E_k |\Phi\rangle\langle\Phi|\right], \tag{S1}$$

here dU and dW are Haar measure, and $|\Phi\rangle = |\psi\rangle^{\otimes N} \otimes |\phi\rangle^{\otimes M} = U^{\otimes(N+M)} |0\rangle^{\otimes N} (W|0\rangle)^{\otimes M}$.

In this work, we mainly consider a qubit case with $d = 2$ and $M = N$. To estimate the value of Eq. (S1) experimentally, we randomly sample qubit pairs using the Haar measure. The Haar-distributed unitary matrix U can be parameterized as [2]:

$$U = \begin{pmatrix} e^{i(\beta-\omega)/2}\cos\frac{\theta}{2} & -e^{-i(\beta+\omega)/2}\sin\frac{\theta}{2} \\ e^{i(\beta+\omega)/2}\sin\frac{\theta}{2} & e^{-i(\beta-\omega)/2}\cos\frac{\theta}{2} \end{pmatrix}, \tag{S2}$$

where β and ω are uniformly distributed on $[0, 2\pi)$, and θ follows a probability density function (PDF) $p(\theta) = \frac{1}{2}\sin\theta$ on $[0, \pi]$. The Haar measure on $SU(2)$ is denoted by $dU = (1/8\pi^2)\sin\theta d\theta d\beta d\omega$. From the fixed overlap expression $c = |\langle 0|W|0\rangle|^2$, we can derive the matrix form of W as

$$W = \begin{pmatrix} \sqrt{c}e^{i(\varphi_g-\varphi)/2} & -\sqrt{1-c}e^{-i(\varphi_g+\varphi)/2} \\ \sqrt{1-c}e^{i(\varphi_g+\varphi)/2} & \sqrt{c}e^{-i(\varphi_g-\varphi)/2} \end{pmatrix}, \tag{S3}$$

where φ_g and φ follow the uniform distribution on $[0, 2\pi)$. Consequently, the qubit pair takes the following form:

$$|\psi\rangle = U|0\rangle, \; |\phi\rangle = U(\sqrt{c}|0\rangle + e^{i\varphi}\sqrt{1-c}|1\rangle), \tag{S4}$$

here we have omitted the global phase term $e^{i(\varphi_g-\varphi)/2}$ in $|\phi\rangle$. It is worth noting that sampling such a unitary W is equivalent to sample a pure state with a fixed angle respect to $|0\rangle$ on the Bloch sphere. By sampling U and φ, we obtain random qubit pairs with a fixed overlap, as illustrated in Fig. S1.

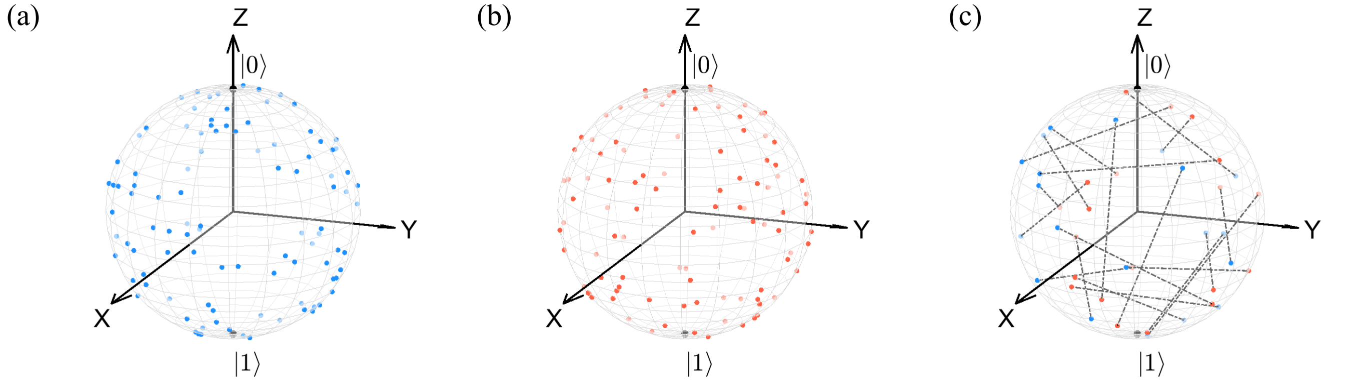

FIG. S1. Bloch sphere representation of pure qubit pairs sampled at a fixed overlap $c = 0.5$. **a** 100 sample states of the qubit state $|\psi\rangle$. **b** 100 sample states of the qubit state $|\phi\rangle$. **c** 20 out of 100 sample qubit pairs represented by dots on the sphere. The dashed lines inside the Bloch sphere have equal lengths, indicating the same overlap between different qubit pairs.

For an overlap estimation strategy denoted by s, which performs a measurement $\hat{E}_k^{(s)}$ on N copies of the qubit pair in Eq. (S4), and uses the estimator $\tilde{c}_s(k)$, the square error can be expressed as

$$v_s(c, N|U, \varphi) = \sum_k [\tilde{c}_s(k) - c]^2 \mathrm{Tr}\left[\hat{E}_k^{(s)}(|\psi\rangle\langle\psi| \otimes |\phi\rangle\langle\phi|)^{\otimes N}\right], \tag{S5}$$

which serves as a function of overlap c and copy number N for given $|\psi\rangle$ and $|\phi\rangle$. From Eq. (S1), the average square error of the strategy s can be written as

$$v_s(c, N) = \frac{1}{2\pi}\int_U \int_0^{2\pi} v_s(c, N|U, \varphi) dU d\varphi, \tag{S6}$$

where dU is the Haar measure of $SU(2)$. The estimators used in our work are unbiased or asymptotically unbiased, hence we also denote $v_s(c, N)$ in Eq. (S6) as average variance of the strategy s. Our analysis shows that as the number of copies N increases, the average variance $v_s(c, N)$ decreases with the scale of $O(1/N)$. Therefore, in the limit of $N \to \infty$, the average variance multiplied by the copy number N, i.e., $Nv_s(c) = \lim_{N\to\infty} N \cdot v_s(c, N)$, only depends on the overlap c. We adopt the scaled average variance $Nv_s(c)$ as a performance assessment for strategy s.

II. TOMOGRAPHY-TOMOGRAPHY AND TOMOGRAPHY-PROJECTION

A. Average infidelity of pure state tomography based on MUB

In this section, we derive an asymptotic solution for the average infidelity in pure qubit state tomography (QST) based on mutually unbiased bases (MUB) and maximum likelihood estimation (MLE). The main text mentioned two local overlap estimation strategies: tomography-tomography (TT) and tomography-projection (TP), both of which require QST based on MUB, i.e., $\{|0\rangle, |1\rangle\}$, $\{|+\rangle, |-\rangle\}$, $\{|L\rangle, |R\rangle\}$ with $|\pm\rangle = (|0\rangle \pm |1\rangle)/\sqrt{2}$ and $|L\rangle, |R\rangle = (|0\rangle \pm i|1\rangle)/\sqrt{2}$. To illustrate the tomography process, we focus on the state $|\psi\rangle$ as an example. Given a total of N copies of $|\psi\rangle$, we measure three Pauli operators $(\hat{\sigma}_x, \hat{\sigma}_y, \hat{\sigma}_z)$ on $N' = N/3$ copies of $|\psi\rangle$ and record the statistics of obtaining the outcome $+1$ for each operator, denoted as (n_x, n_y, n_z). The probabilities of obtaining the outcome $+1$ for the three Pauli operators are as follows

$$p_x = |\langle\psi|+\rangle|^2 = \frac{1}{2}(1 + \sin\theta\cos\omega),\ p_y = |\langle\psi|L\rangle|^2 = \frac{1}{2}(1 + \sin\theta\sin\omega),\ p_z = |\langle\psi|0\rangle|^2 = \cos^2\frac{\theta}{2}, \tag{S7}$$

where $|\psi\rangle = U|0\rangle$ and U is defined in Eq. (S2). Given that $|\psi\rangle$ is pure, we obtain the reconstructed state $|\tilde{\psi}\rangle$ by finding a straightforward yet approximate solution to the maximum likelihood equations in the following form:

$$|\tilde{\psi}\rangle = \sqrt{\frac{n_z}{N'}}|0\rangle + \sqrt{1 - \frac{n_z}{N'}}\frac{(\frac{2n_x}{N'} - 1) + i(\frac{2n_y}{N'} - 1)}{\sqrt{(\frac{2n_x}{N'} - 1)^2 + (\frac{2n_y}{N'} - 1)^2}}|1\rangle. \tag{S8}$$

The tomography result is determined by the outcome (n_x, n_y, n_z) and follows three independent binomial distributions with a PDF as follows:

$$P_{tomo}\left(\tilde{\psi}(n_x, n_y, n_z)|N, U\right) = \mathrm{Bin}(n_x, N', p_x)\mathrm{Bin}(n_y, N', p_y)\mathrm{Bin}(n_z, N', p_z), \tag{S9}$$

where $\mathrm{Bin}(k, N, p) = \binom{N}{k}p^k(1-p)^{N-k}$ is the PDF of the binomial distribution, and p_x, p_y, p_z are defined in Eq. (S7). The fidelity between the true state and the reconstructed state is defined as $F = |\langle\psi|\tilde{\psi}\rangle|^2$ for $|\psi\rangle$ and $|\tilde{\psi}\rangle$. For a given U, the fidelity of the above tomography procedure is given by

$$\begin{aligned}
F_U =& \sum_{n_x, n_y, n_z} |\langle\psi|\tilde{\psi}\rangle|^2 P_{tomo}\left(\tilde{\psi}(n_x, n_y, n_z)|N, U\right) \\
=&\frac{1}{2}\left\langle 1 + \left(\frac{2n_z}{N'} - 1\right)\cos\theta + 2\sin\theta\sqrt{\frac{n_z}{N'}\left(1 - \frac{n_z}{N'}\right)}\frac{(\frac{2n_x}{N'} - 1)\cos\omega + \left(\frac{2n_y}{N'} - 1\right)\sin\omega}{\sqrt{\left(\frac{2n_x}{N'} - 1\right)^2 + \left(\frac{2n_y}{N'} - 1\right)^2}}\right\rangle_{n_x, n_y, n_z} \\
=&\frac{1}{2}\left[1 + \left\langle\frac{2n_z}{N'} - 1\right\rangle_{n_z}\cos\theta + 2\sin\theta\left\langle\sqrt{\frac{n_z}{N'}\left(1 - \frac{n_z}{N'}\right)}\right\rangle_{n_z}\left\langle\frac{(\frac{2n_x}{N'} - 1)\cos\omega + \left(\frac{2n_y}{N'} - 1\right)\sin\omega}{\sqrt{\left(\frac{2n_x}{N'} - 1\right)^2 + \left(\frac{2n_y}{N'} - 1\right)^2}}\right\rangle_{n_x, n_y}\right],
\end{aligned} \tag{S10}$$

where the notation $\langle\cdot\rangle_j$ represents the expectation with respect to the random variable j. We introduce the following notations:

$$X = 2n_x/N' - 1,\ Y = 2n_y/N' - 1,\ Z = n_z/N'. \tag{S11}$$

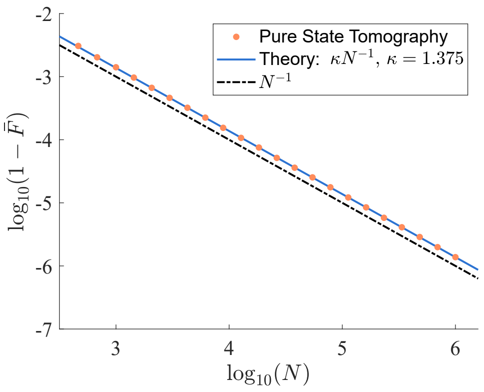

FIG. S2. Average infidelity $1-\overline{F}$ v.s. tomography copy number N for Monte Carlo simulations of the pure state tomography. For each N, we uniformly sample 1000 different qubit states and perform 20 repeated tomographic simulations for each qubit. The simulated results (orange dots) show excellent agreement with the theoretical result (blue line).

Using these notations, we have the following expressions:

$$\begin{aligned}
\langle X\rangle_{n_x} &= \sin\theta\cos\omega,\ \sigma_X^2 = \langle (X-\langle X\rangle_{n_x})^2\rangle_{n_x} = \frac{1-\sin^2\theta\cos^2\omega}{N'},\\
\langle Y\rangle_{n_y} &= \sin\theta\sin\omega,\ \sigma_Y^2 = \langle (Y-\langle Y\rangle_{n_y})^2\rangle_{n_y} = \frac{1-\sin^2\theta\sin^2\omega}{N'},\\
\langle Z\rangle_{n_z} &= \cos^2\frac{\theta}{2},\ \sigma_Z^2 = \langle (Z-\langle Z\rangle_{n_z})^2\rangle_{n_z} = \frac{\sin^2\theta}{4N'},
\end{aligned} \tag{S12}$$

where we can observe that the higher central moments of X, Y and Z are $O(1/N'^2)$ orders. Additionally, we define two functions as follows:

$$f(Z) = \sqrt{Z(1-Z)},\ g(X,Y) = \frac{X\cos\omega + Y\sin\omega}{\sqrt{X^2+Y^2}}. \tag{S13}$$

From the probability theory [3], the expectations of Eq. (S13) can be expanded as

$$\begin{aligned}
\langle f(Z)\rangle_{n_z} =& f(\langle Z\rangle_{n_z}) + \frac{d^2 f}{dZ^2}\Big|_{\langle Z\rangle_{n_z}}\frac{\sigma_Z^2}{2} + O(\frac{1}{N'^2}) \approx \frac{\sin\theta}{2} - \frac{1}{4N'\sin\theta}\\
\langle g(X,Y)\rangle_{n_x,n_y} =& g(\langle X\rangle_{n_x},\langle Y\rangle_{n_y}) + \frac{\partial^2 g}{\partial X^2}\Big|_{\langle X\rangle_{n_x},\langle Y\rangle_{n_y}}\frac{\sigma_X^2}{2} + \frac{\partial^2 g}{\partial Y^2}\Big|_{\langle X\rangle_{n_x},\langle Y\rangle_{n_y}}\frac{\sigma_Y^2}{2} + O(\frac{1}{N'^2})\\
\approx& 1 - \frac{\sin^2\omega(1-\sin^2\theta\cos^2\omega)}{2N'\sin^2\theta} - \frac{\cos^2\omega(1-\sin^2\theta\sin^2\omega)}{2N'\sin^2\theta}\\
=& 1 - \frac{1-2\sin^2\theta\sin^2\omega\cos^2\omega}{2N'\sin^2\theta}.
\end{aligned} \tag{S14}$$

F_U in Eq. (S10) is then given by

$$\begin{aligned}
F_U =& \frac{1}{2}\left[1 + (2\langle Z\rangle_{n_z} - 1)\cos\theta + 2\sin\theta\langle f(Z)\rangle_{n_z}\langle g(X,Y)\rangle_{n_x,n_y}\right]\\
=& 1 - \frac{1-\sin^2\theta\sin^2\omega\cos^2\omega}{2N'} + O(\frac{1}{N'^2}).
\end{aligned} \tag{S15}$$

After averaging over U, the average fidelity of pure qubit tomography based on MUB is then given by

$$\begin{aligned}
\overline{F} = \int_U F_U dU =& \frac{1}{4\pi}\int_0^{\pi}\int_0^{2\pi}(1 - \frac{1-\sin^2\theta\sin^2\omega\cos^2\omega}{2N'})\sin\theta d\theta d\omega + O(\frac{1}{N'^2})\\
=& 1 - \frac{11}{24N'} + O(\frac{1}{N'^2}) \approx 1 - \frac{11}{8N},
\end{aligned} \tag{S16}$$

where $N = 3N'$. Neglecting the high-order terms, the average infidelity $1-\overline{F} = 11/8N$ scales as $O(1/N)$ and the scaled average infidelity is given by

$$\kappa = N(1-\overline{F}) = \frac{11}{8}. \tag{S17}$$

Furthermore, we validate this theoretical result through Monte Carlo simulations of pure state tomography. Figure S2 presents the plot of simulated average infidelity against the copy number N in tomography. We fit the simulated data to the form $1-\overline{F} = \kappa N^{-1}$ and find $\kappa = 1.377 \pm 0.006$ which agree with the theoretical result. Furthermore, We note that with an optimal guess of the reconstructed state using the Bayesian approach in [4], the coefficient κ can be improved to 13/12 with static measurements. By using the adaptive approach for pure qubit tomography, κ can be further improved to 1, saturating the collective measurement bound given by [5].

B. Tomography error analysis

In this section, we derive the asymptotic results for the average error distribution of the reconstructed states given by QST. Starting from Eq. (S4), we can express the orthogonal states of $|\psi\rangle$ and $|\phi\rangle$ as

$$|\psi_\perp\rangle = U|1\rangle\,,\ |\phi_\perp\rangle = U(\sqrt{1-c}\,|0\rangle - e^{i\varphi}\sqrt{c}\,|1\rangle), \tag{S18}$$

here, we have $\langle\psi|\phi_\perp\rangle = \sqrt{1-c}$, $\langle\psi_\perp|\phi_\perp\rangle = -e^{i\varphi}\sqrt{c}$ and $\langle\psi_\perp|\phi\rangle = e^{i\varphi}\sqrt{1-c}$. The reconstructed states of $|\phi\rangle$ and $|\psi\rangle$ by QST can be represented as

$$|\tilde{\psi}\rangle = \cos\frac{\chi_1}{2}|\psi\rangle + \sin\frac{\chi_1}{2}e^{i\zeta_1}|\psi_\perp\rangle\,,\ |\tilde{\phi}\rangle = \cos\frac{\chi_2}{2}|\phi\rangle + \sin\frac{\chi_2}{2}e^{i\zeta_2}|\phi_\perp\rangle\,, \tag{S19}$$

where $\chi_j \in [0,\pi]$ (for $j = 1,2$) denote the deviation of the estimate states from the true state, and $\zeta_j \in [0, 2\pi)$ (for $j = 1,2$) are random phases introduced by QST. The joint PDFs of χ_j and ζ_j are denoted by $p(\chi_1,\zeta_1|N,U)$ and $p(\chi_2,\zeta_2|c,N,U,\varphi)$, which depend on the specific values of U and φ. In this supplementary materials, we use the notation $\langle\cdot\rangle$ to represent the expectation respect to these distributions under a given pair of U and φ. For instance, $\langle\chi_1\rangle = \int_{\chi_1}\int_{\zeta_1}\chi_1 p(\chi_1,\zeta_1|N,U)d\chi_1 d\zeta_1$. On the other hand, the notation $\overline{\langle\cdot\rangle}$ denotes the average expectation over all possible values of U and φ. For example

$$\overline{\langle\chi_2\rangle} = \frac{1}{2\pi}\int_U\int_0^{2\pi}\langle\chi_2\rangle\,dUd\varphi = \frac{1}{2\pi}\int_U\int_0^{2\pi}\left[\int_{\chi_2}\int_{\zeta_2}\chi_2 p(\chi_2,\zeta_2|c,N,U,\varphi)d\chi_2 d\zeta_2\right]dUd\varphi. \tag{S20}$$

Taking $|\tilde{\psi}\rangle$ as an example to analyze the error distribution for the pure state tomography, we temporarily omit the lower indices of χ_j and ζ_j in this section. Considering a Bloch sphere with that z axis represents the true state $|\psi\rangle$, then χ and ζ in $|\tilde{\psi}\rangle$ correspond the polar and azimuthal angles, respectively, as illustrated in Fig. S3. To simplify the analysis, we introduce a coordinate transformation:

$$t^c = \sin\chi\cos\zeta,\ t^s = \cos\chi\sin\zeta, \tag{S21}$$

the two error variables (t^c, t^s) follows a joint PDF denoted as $p(t^c, t^s|N,U)$, which can be derived from $p(\chi,\zeta|N,U)$ with the transformation. By building a relationship between (t^c, t^s) and three independent variables (X,Y,Z) defined in Eq. (S8), namely, the recording statistics (n_x, n_y, n_z) in the tomography, we can derive the means and variances of (t^c, t^s) for a given U and integrate them over $SU(2)$ to get the average moments.

Firstly, we rewrite the $|\tilde{\psi}\rangle$ in Eq. (S19) on the computational basis $\{|0\rangle, |1\rangle\}$ as follows:

$$\begin{aligned}
|\tilde{\psi}\rangle &= \sqrt{A}\,|0\rangle + e^{iB}\sqrt{1-A}\,|1\rangle\,,\ A = \frac{1}{2}\left(1+\cos\chi\cos\theta - \sin\chi\cos(\beta-\zeta)\sin\theta\right),\\
\cos B &= \frac{G_X}{2\sqrt{A(1-A)}},\ G_X = [\cos\chi\sin\theta + \sin\chi\cos(\beta-\zeta)\cos\theta]\cos\omega + \sin\chi\sin(\beta-\zeta)\sin\omega,\\
\sin B &= \frac{G_Y}{2\sqrt{A(1-A)}},\ G_Y = [\cos\chi\sin\theta + \sin\chi\cos(\beta-\zeta)\cos\theta]\sin\omega - \sin\chi\sin(\beta-\zeta)\cos\omega,
\end{aligned} \tag{S22}$$

where θ, β and ω are from the parameterized U defined in Eq. (S2) and we have discarded a global phase. Recalling X, Y and Z defined in Eq. (S8), we have another expression of $|\tilde{\psi}\rangle$ as

$$|\tilde{\psi}\rangle = \sqrt{Z}\,|0\rangle + \sqrt{1-Z}\frac{X+iY}{\sqrt{X^2+Y^2}}. \tag{S23}$$

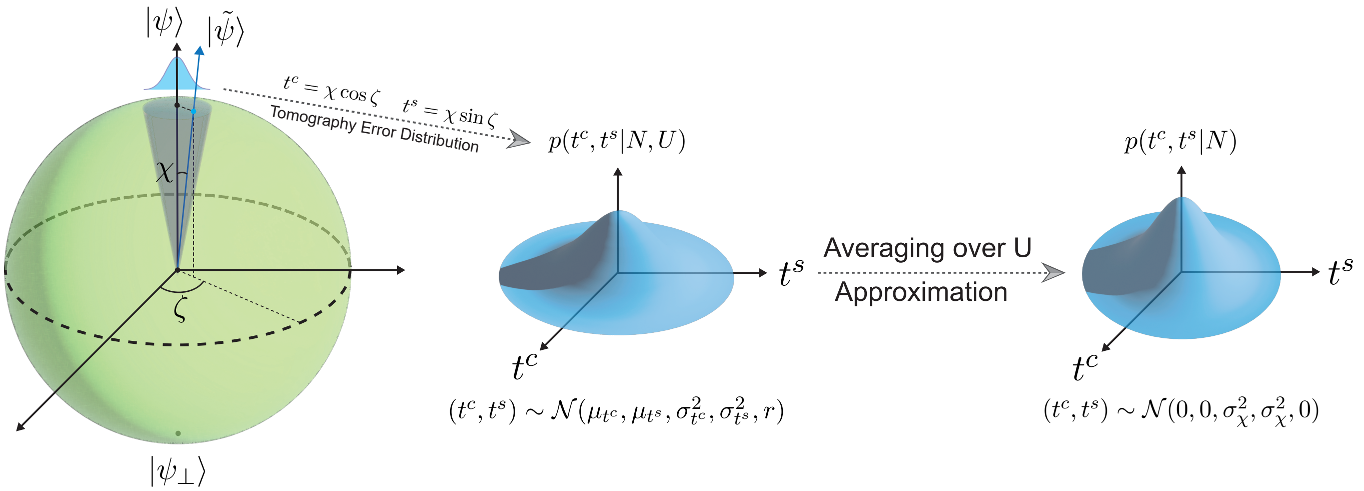

FIG. S3. Schematic of the error distribution in pure qubit MUB tomography. t^c and t^s are obtained through coordinate transformation from ζ and χ. The error probability distribution $p(t^c, t^s|N, U)$, under a specific unitary operator U, can be approximated as a bivariate Gaussian distribution of t^c and t^s. When averaging over all possible U, the average distribution $p(t^c, t^s|N)$ can be approximated as a bivariate Gaussian distribution with the same variance for the marginal distributions. This approximation implies that t^c and t^s are statistically independent in the average case.

Combining two expressions of $|\tilde{\psi}\rangle$ in Eq. (S22) and Eq. (S23), we can get the following equations:

$$G_X = \frac{2X\sqrt{Z(1-Z)}}{\sqrt{X^2+Y^2}},\ G_Y = \frac{2Y\sqrt{Z(1-Z)}}{\sqrt{X^2+Y^2}},\ A = Z. \tag{S24}$$

We can rewrite G_X and G_Y with (t^c, t^s) as follows:

$$\begin{aligned} G_X &= \left[\frac{\cos\beta\cos\omega}{\cos\theta} + \sin\beta\sin\omega\right]t^c + \left[\frac{\sin\beta\cos\omega}{\cos\theta} - \cos\beta\sin\omega\right]t^s + (2Z-1)\cos\omega\tan\theta, \\ G_Y &= \left[\frac{\cos\beta\sin\omega}{\cos\theta} - \sin\beta\cos\omega\right]t^c + \left[\frac{\sin\beta\sin\omega}{\cos\theta} + \cos\beta\cos\omega\right]t^s + (2Z-1)\sin\omega\tan\theta. \end{aligned} \tag{S25}$$

Here we have build a relationship between (X, Y, Z) and (t^c, t^s) through G_X and G_Y. Then we can express t^c and t^s as follows:

$$\begin{aligned} t^c &= (\cos\theta\cos\beta\cos\omega + \sin\beta\sin\omega)G_X + (\cos\theta\cos\beta\sin\omega - \sin\beta\cos\omega)G_Y - (2Z-1)\sin\theta\cos\beta, \\ t^s &= (\cos\theta\sin\beta\cos\omega - \cos\beta\sin\omega)G_X + (\cos\theta\sin\beta\sin\omega + \cos\beta\cos\omega)G_Y - (2Z-1)\sin\theta\sin\beta. \end{aligned} \tag{S26}$$

In the case of large N, with the same approach in Eq. (S14), the means of G_X and G_Y can be derived through the moments of (X, Y, Z) in Eq. (S12) as

$$\begin{aligned} \langle G_X\rangle &= \sin\theta\cos\omega - \frac{\cos\omega}{\sin\theta N'} + \frac{(1+3\cos 2\omega)\sin^2\omega\cos\omega\sin\theta}{2N'} + O(\frac{1}{N'^2}), \\ \langle G_Y\rangle &= \sin\theta\sin\omega - \frac{\sin\omega}{\sin\theta N'} + \frac{(1-3\cos 2\omega)\sin\omega\cos^2\omega\sin\theta}{2N'} + O(\frac{1}{N'^2}), \end{aligned} \tag{S27}$$

with $N' = N/3$. Combining Eq. (S26) with Eq. (S27), the means of t^c and t^s are then given by

$$\begin{aligned} \mu_{t^c} = \langle t^c\rangle &= \frac{\sin\beta}{4N'}\sin\theta\sin 4\omega + \frac{\cos\beta}{4N'}\left(\sin\theta\cos\theta\sin^2 2\omega - 4\cot\theta\right) + O(\frac{1}{N'^2}), \\ \mu_{t^s} = \langle t^s\rangle &= -\frac{\cos\beta}{4N'}\sin\theta\sin 4\omega + \frac{\sin\beta}{4N'}\left(\sin\theta\cos\theta\sin^2 2\omega - 4\cot\theta\right) + O(\frac{1}{N'^2}). \end{aligned} \tag{S28}$$

Here, we show that μ_{t^c} and μ_{t^s} scale as $O(1/N)$, resulting from the asymptotic unbiasedness of maximum likelihood estimation. To obtain the variance of (t^c, t^s), we expand t^c and t^s in Eq. (S26) as functions of (X, Y, Z) up to the

first order terms around the mean point $(\langle X\rangle, \langle Y\rangle, \langle Z\rangle)$, which are given by:

$$\begin{aligned} t^c &= \sin\beta\sin\omega X - \sin\beta\cos\omega Y - \frac{\cos\beta}{\sin\theta}(2Z-1-\cos\theta) + \text{high-order terms}, \\ t^s &= -\cos\beta\sin\omega X + \cos\beta\cos\omega Y - \frac{\sin\beta}{\sin\theta}(2Z-1-\cos\theta) + \text{high-order terms}. \end{aligned} \tag{S29}$$

The leading order of variance is only determined by the first-order terms in t_c and t_s. Consequently, we can derive the variance ($\sigma^2_{t^c}$, $\sigma^2_{t^s}$) and correlation coefficient r as

$$\begin{aligned} \sigma^2_{t^c} &= \frac{1-2\sin^2\theta\sin^2\omega\cos^2\omega\sin^2\beta}{N'} + O(\frac{1}{N'^2}),\ \sigma^2_{t^s} = \frac{1-2\sin^2\theta\sin^2\omega\cos^2\omega\cos^2\beta}{N'} + O(\frac{1}{N'^2}), \\ r &= \left[\frac{\sin 2\beta\left(1-\sin^2\theta\sin^2\omega\cos^2\omega\right)}{N'} + O(\frac{1}{N'^2})\right]/\sigma_{t^c}\sigma_{t^s}. \end{aligned} \tag{S30}$$

Through integrating the results over U, we can get the means, variances and second-order raw moments in the average case as follows:

$$\begin{aligned} \overline{\mu_{t^c}} &= \overline{\langle t^c\rangle} = O(\frac{1}{N^2}), \quad \overline{\sigma^2_{t^c}} = \frac{11}{4N} + O(\frac{1}{N^2}), \quad \overline{\langle (t^c)^2\rangle} = \overline{\sigma^2_{t^c}} + \overline{\langle t^c\rangle^2} = \frac{11}{4N} + O(\frac{1}{N^2}), \\ \overline{\mu_{t^s}} &= \overline{\langle t^s\rangle} = O(\frac{1}{N^2}), \quad \overline{\sigma^2_{t^s}} = \frac{11}{4N} + O(\frac{1}{N^2}), \quad \overline{\langle (t^s)^2\rangle} = \overline{\sigma^2_{t^s}} + \overline{\langle t^s\rangle^2} = \frac{11}{4N} + O(\frac{1}{N^2}), \\ & \qquad \overline{\langle t^c t^s\rangle} = \overline{r\sigma_{t^c}\sigma_{t^s}} = O(\frac{1}{N^2}), \end{aligned} \tag{S31}$$

where $\overline{\langle\cdot\rangle}$ denotes

$$\overline{\langle\cdot\rangle} = \int_U \langle\cdot\rangle\, dU = \frac{1}{8\pi^2}\int_0^{\pi}\int_0^{2\pi}\int_0^{2\pi}(\cdot)\sin\theta d\theta d\beta d\omega. \tag{S32}$$

In the asymptotic limit, the average fidelity shown in Eq. (S16) can be derived through the distribution of χ as

$$\overline{F} = \overline{\left\langle \cos^2\frac{\chi}{2}\right\rangle} \approx \overline{\left\langle 1-\frac{\chi^2}{4}\right\rangle} = 1 - \frac{\overline{\langle\chi^2\rangle}}{4}, \tag{S33}$$

where we use the approximation $\cos^2\chi/2 \approx 1-\chi^2/4$. Together with Eq. (S16), we can build the relationship between $\overline{\langle\chi^2\rangle}$ and the scaled average infidelity κ as

$$\overline{\langle\chi^2\rangle} = \frac{4\kappa}{N},\ \kappa = \frac{11}{8}. \tag{S34}$$

The result can also be derived from the average moments of (t^c, t^s) as

$$\overline{\langle\chi^2\rangle} \approx \overline{\langle\sin^2\chi\rangle} = \overline{\langle (t^c)^2 + (t^s)^2\rangle} = \overline{\langle (t^c)^2\rangle} + \overline{\langle (t^s)^2\rangle} = \frac{11}{2N} + O(\frac{1}{N^2}) \approx \frac{4\kappa}{N}, \tag{S35}$$

where we use the approximation $\sin\chi \approx \chi$. We define $\sigma^2_\chi = 2\kappa/N$, and find that

$$\overline{\sigma^2_{t_c}} \approx \overline{\langle (t^c)^2\rangle} \approx \overline{\sigma^2_{t_s}} \approx \overline{\langle (t^s)^2\rangle} \approx \frac{\overline{\langle\chi^2\rangle}}{2} \approx \sigma^2_\chi = \frac{2\kappa}{N}. \tag{S36}$$

To gain an intuitive understanding of the results, we can approximate the joint PDF $p(t^c, t^s|N,U)$ as a bivariate Gaussian distribution $\mathcal{N}(\mu_{t^c}, \mu_{t^s}, \sigma^2_{t^c}, \sigma^2_{t^s}, r)$ with means (μ_{t^c}, μ_{t^s}), variances $(\sigma^2_{t^c}, \sigma^2_{t^s})$, and correlation coefficient r. Taking the average over U yields an average distribution $p(t^c, t^s|N) = \overline{p(t^c, t^s|N,U)}$, which can be approximated as $\mathcal{N}(0, 0, \sigma^2_\chi, \sigma^2_\chi, 0)$ by neglecting terms that scale as $O(1/N^2)$. This implies that averaging over $SU(2)$ naturally generates a symmetric distribution of tomography errors, as depicted in Fig. S3. Then the average distributions of χ and ζ can be approximated by Rayleigh distribution and uniform distribution with PDFs as $\overline{p(\chi,\zeta|N,U)} \sim p(\chi|N)p(\zeta)$, $p(\chi|N) = \chi/\sigma^2_\chi \exp\left(-\chi^2/2\sigma^2_\chi\right)$, $p(\zeta) = 1/2\pi$.

The above we have discussed is about χ_1 and ζ_1 in $|\tilde{\psi}\rangle$, and the same goes for χ_2 and ζ_2 in $|\tilde{\phi}\rangle$. With the invariant of Haar measure, we can show that

$$\begin{aligned}\overline{p(\chi_2,\zeta_2|c,N,U,\varphi)} &= \frac{1}{2\pi}\int_U\int_0^{2\pi} p(\chi_2,\zeta_2|c,N,U,\varphi)dUd\varphi \\ &= \frac{1}{2\pi}\int_{UW^\dagger}\int_0^{2\pi} p(\chi_2,\zeta_2|c,N,UW^\dagger,\varphi)d(UW^\dagger)d\varphi \\ &= \int_U p(\chi_1,\zeta_1|N,U)dU = \overline{p(\chi_1,\zeta_1|N,U)},\end{aligned} \tag{S37}$$

where $p(\chi_2,\zeta_2|c,N,U,\varphi) = p(\chi_1,\zeta_1|N,UW)$ and W is defined in Eq. (S3). The average distributions of the tomography error for both $|\psi\rangle$ and $|\phi\rangle$ is identical, thanks to the reference-frame average.

C. Tomography-Tomography strategy precision

In this section, we derive the theoretical results for the average overlap estimation precision in the TT strategy. The overlap between two reconstructed states $|\tilde{\psi}\rangle$ and $|\tilde{\phi}\rangle$ in Eq. (S19) is given by

$$\begin{aligned}\tilde{c}_{tt} = |\langle\tilde{\phi}|\tilde{\psi}\rangle|^2 = \frac{1}{2}\Big[&1 + (2c-1)\cos\chi_1\cos\chi_2 + 2\sqrt{c(1-c)}\big[\cos(\zeta_1-\varphi)\sin\chi_1\cos\chi_2 + \cos\zeta_2\cos\chi_1\sin\chi_2\big] \\ &- (2c-1)\cos(\zeta_1-\varphi)\cos\zeta_2\sin\chi_1\sin\chi_2 - \sin(\zeta_1-\varphi)\sin\zeta_2\sin\chi_1\sin\chi_2\Big].\end{aligned} \tag{S38}$$

Average mean. Firstly, we derive the average mean of the estimated overlap in TT strategy. With the error variables defined in Eq. (S21), we rewrite the expression of $\tilde{c}_{tt}$ by ignoring terms higher than the second order as

$$\begin{aligned}\tilde{c}_{tt} \approx\ & c + \sqrt{c(1-c)}\,(t_1^c\cos\varphi + t_1^s\sin\varphi + t_2^c) - \frac{2c-1}{4}(\chi_1^2+\chi_2^2) \\ &- \frac{2c-1}{2}(t_1^c\cos\varphi + t_1^s\sin\varphi)t_2^c - \frac{1}{2}(t_1^c\sin\varphi - t_1^s\cos\varphi)t_2^s,\end{aligned} \tag{S39}$$

then the mean is given by

$$\begin{aligned}\overline{\langle\tilde{c}_{tt}\rangle} &= \frac{1}{2\pi}\int_U\int_\varphi\int_{\zeta_1,\zeta_2,\chi_1,\chi_2} \tilde{c}_{tt}p(\chi_1,\zeta_1|N,U)p(\chi_2,\zeta_2|c,N,U,\varphi)dUd\varphi d\zeta_1 d\zeta_2 d\chi_1 d\chi_2 \\ &= c - \frac{2c-1}{4}\left(\overline{\langle\chi_1^2\rangle} + \overline{\langle\chi_2^2\rangle}\right) + O(\frac{1}{N^2}) \\ &= c - \frac{2\kappa}{N}(2c-1) + O(\frac{1}{N^2}),\end{aligned} \tag{S40}$$

where we use $\overline{\langle t_1^c\rangle}, \overline{\langle t_1^s\rangle}, \overline{\langle t_2^c\rangle}, \overline{\langle t_2^s\rangle}, \langle t_1^c t_2^c\rangle, \langle t_1^s t_2^c\rangle, \langle t_1^c t_2^s\rangle, \langle t_1^s t_2^s\rangle = O(1/N^2)$ and Eq. (S34). Here, we show that the overlap estimator is asymptotically unbiased in TT strategy.

Average variance. With ignoring terms higher than the second order, the squared error can be expressed as

$$\begin{aligned}(\tilde{c}_{tt}-c)^2 &\approx c(1-c)\,(t_1^c\cos\varphi + t_1^s\sin\varphi + t_2^c)^2 \\ &= c(1-c)\left[(t_1^c)^2\cos^2\varphi + (t_1^s)^2\sin^2\varphi + (t_2^c)^2 + t_1^c t_1^s\sin 2\varphi + 2t_1^c t_2^c\cos\varphi + 2t_1^s t_2^c\sin\varphi\right].\end{aligned} \tag{S41}$$

The average variance of overlap for TT strategy is then given by

$$\begin{aligned}v_{tt} = \overline{\langle(\tilde{c}_{tt}-c)^2\rangle} &= c(1-c)\left(\frac{1}{2}\overline{\langle(t_1^c)^2\rangle} + \frac{1}{2}\overline{\langle(t_1^s)^2\rangle} + \overline{\langle(t_2^c)^2\rangle}\right) + O(\frac{1}{N^2}) \\ &= \frac{c(1-c)}{2}\left(\overline{\langle\chi_1^2\rangle} + \overline{\langle\chi_2^2\rangle}\right) + O(\frac{1}{N^2}) \\ &= \frac{4\kappa c(1-c)}{N} + O(\frac{1}{N^2}).\end{aligned} \tag{S42}$$

with Eq. (S36). We have shown a direct relationship between the tomography infidelity and overlap estimation precision in the TT strategy. The overlap estimation error in the TT strategy originates from tomography error, and both errors scale as $O(1/N)$.

Fisher information. We treat $\tilde{c}$ as a random variable and aim to determine its PDF $p(\tilde{c}|c,N)$ which depends on the true overlap c. After averaging over U, we approximate the average tomography error distribution $p(t^c,t^s|N)$ as the bivariate Gaussian distribution $\mathcal{N}(0,0,\sigma_\chi^2,\sigma_\chi^2,0)$. Then χ_j will follow the Rayleigh distribution, while $\zeta_1-\varphi$ and ζ_2 follow the uniform distribution. $t_1'=\chi_1\cos(\zeta_1-\varphi)$ and $t_2'=\chi_2\cos\zeta_2$ are independent and both follow the same Gaussian distribution with the PDF $p(t_j')=1/\sqrt{2\pi}\sigma_\chi\exp(-(t_j')^2/2\sigma_\chi^2),\ j=1,2$. By retaining only the first-order terms, we can rewrite Eq. (S38) as follows:

$$\frac{\tilde{c}_{tt}-c}{\sqrt{c(1-c)}}\approx\chi_1\cos(\zeta_1-\varphi)+\chi_2\cos\zeta_2=t_1'+t_2'=T\sim\mathcal{N}(0,2\sigma_\chi^2), \tag{S43}$$

where $\mathcal{N}(\mu,\sigma^2)$ denotes the Gaussian distribution with mean μ and variance σ^2. We can observe that the estimated overlap $\tilde{c}_{tt}$ will asymptotically follow a Gaussian distribution $\mathcal{N}(c,2c(1-c)\sigma_\chi^2)$. Furthermore, we can also rederive the variance v_{tt} as mentioned in Eq. (S42). The Fisher information (FI) of overlap in TT strategy is then given by

$$\begin{aligned}I_{tt}(c)&=\left\langle\left(\frac{\partial\log p(\tilde{c}|c,N)}{\partial c}\right)^2\right\rangle_{\tilde{c}}\\&=\frac{(c-2(1-c)(2c-1)\sigma_\chi^2)^2}{16c^2(1-c)^4\sigma_\chi^4}+\frac{c-2(1-c)(2c-1)\sigma_\chi^2}{4c(1-c)^4\sigma_\chi^4}\langle\tilde{c}\rangle_{\tilde{c}}\\&\quad+\frac{c(2c^2+2c-1)-2(1-c)(2c-1)^2\sigma_\chi^2}{8c^3(1-c)^4\sigma_\chi^4}\langle\tilde{c}^2\rangle_{\tilde{c}}-\frac{(2c-1)}{4c^2(1-c)^4\sigma_\chi^4}\langle\tilde{c}^3\rangle_{\tilde{c}}+\frac{(2c-1)^2}{16c^4(1-c)^4\sigma_\chi^4}\langle\tilde{c}^4\rangle_{\tilde{c}}\\&=\frac{1-4c(1-c)}{2c^2(1-c)^2}+\frac{1}{2c(1-c)\sigma_\chi^2}=\frac{1-4c(1-c)}{2c^2(1-c)^2}+\frac{N}{4\kappa c(1-c)}\approx\frac{N}{4\kappa c(1-c)}\end{aligned} \tag{S44}$$

where we use the expressions of high order moments of Gaussian distribution in the third equal sign.

D. Tomography-Projection strategy precision

In this section, we derive the theoretical results for the average overlap estimation precision in the TP strategy. After the tomography procedure, we get the reconstructed state $|\tilde{\phi}\rangle$ as in Eq. (S19). Then in the projection procedure, the probability of successfully projecting the state $|\psi\rangle$ onto $|\tilde{\phi}\rangle$ is given by

$$p_{tp}=|\langle\psi|\tilde{\phi}\rangle|^2=\frac{1}{2}\big[1+(2c-1)\cos\chi_2+2\sqrt{c(1-c)}\cos\zeta_2\sin\chi_2\big]. \tag{S45}$$

The expression of p_{tp} can be expand to the second order as follows:

$$p_{tp}\approx c+\sqrt{c(1-c)}t_2^c-\frac{2c-1}{4}\chi_2^2. \tag{S46}$$

After conducting N trials of projection, the number of successful projections follows a binomial distribution, denoted as $k\sim\mathrm{Bin}(k,N,p_{tp})$. The overlap estimator in TP strategy is given by $\tilde{c}_{tp}=k/N$.

Average mean. The average mean of $\tilde{c}_{tp}$ is given by

$$\begin{aligned}\overline{\langle\tilde{c}_{tp}\rangle}&=\int_U\int_{\zeta_2,\chi_2}\left\langle\frac{k}{N}\right\rangle_k p(\chi_2,\zeta_2|N,U)dUd\zeta_2d\chi_2=\overline{\langle p_{tp}\rangle}\\&=c-\frac{2c-1}{4}\overline{\langle\chi_2^2\rangle}=c-\frac{\kappa}{N}(2c-1)+O(\frac{1}{N^2}),\end{aligned} \tag{S47}$$

where $\langle\cdot\rangle_k$ denotes the expectation with respect to k according to the binomial distribution and $\langle\cdot\rangle$ represents the expectation with respect to variables in p_{tp}. We neglect the integration respect to φ due to the reference-frame average. Here, we show that the overlap estimator is asymptotically unbiased in TP strategy.

Average variance. The average variance of TP strategy is given by

$$v_{tp}=\int_U\int_{\zeta_2,\chi_2}\left\langle(\frac{k}{N}-c)^2\right\rangle_k p(\chi_2,\zeta_2|N,U)dUd\zeta_2d\chi_2. \tag{S48}$$

From the binomial distribution, the expectation of squared error with respect to k can be derived as

$$\begin{aligned}\left\langle(\frac{k}{N}-c)^2\right\rangle_k &= \frac{1}{N^2}\left\langle k^2\right\rangle_k - \frac{2c}{N}\left\langle k\right\rangle_k + c^2 = \frac{1}{N^2}\cdot\left[Np_{tp}(1-p_{tp}+N^2p_{tp}^2)\right] - \frac{2c}{N}\cdot Np_{tp} + c^2 \\ &= \frac{p_{tp}(1-p_{tp})}{N} + (p_{tp}-c)^2.\end{aligned} \tag{S49}$$

The expectations of $p_{tp}(1-p_{tp})$ and $(p_{tp}-c)^2$ in the average case are given by

$$\begin{aligned}\overline{\langle p_{tp}(1-p_{tp})\rangle} &= c(1-c) + O(\frac{1}{N}), \\ \overline{\langle (p_{tp}-c)^2\rangle} &= c(1-c)\overline{\langle (t_2^c)^2\rangle} + O(\frac{1}{N^2}) = \frac{2\kappa c(1-c)}{N} + O(\frac{1}{N^2}).\end{aligned} \tag{S50}$$

Then v_{tp} in Eq. (S48) can be derived as

$$v_{tp} = \overline{\left\langle \frac{p_{tp}(1-p_{tp})}{N}\right\rangle} + \overline{\langle (p_{tp}-c)^2\rangle} = \frac{(1+2\kappa)c(1-c)}{N} + O(\frac{1}{N^2}). \tag{S51}$$

The variance of TP strategy can be decomposed into two components. The first component, $\overline{\langle p_{tp}(1-p_{tp})\rangle}/N$, denotes the error introduced by the finite number of projections. The second component, $\overline{\langle (p_{tp}-c)^2\rangle}$, represents the error introduced by the deviation of $|\tilde{\phi}\rangle$ from $|\phi\rangle$ during the tomography procedure.

Fisher information. In TP strategy, the probability distribution of k after averaging respect to χ_2 and ζ_2 can be expressed as:

$$p(k|c,N) = \overline{\left\langle \binom{N}{k} p_{tp}^k (1-p_{tp})^{N-k}\right\rangle}, \tag{S52}$$

The FI of overlap in TP strategy with N copies of qubit pairs is then given by

$$I_{tp}(c) = \left\langle \left(\frac{\partial \log p(k|c,N)}{\partial c}\right)^2\right\rangle_k. \tag{S53}$$

It is challenging to calculate this expression analytically. Instead, we numerically evaluate Eq. (S53) with $N = 900$, which corresponds to our experimental setting. The numerical results are shown in Fig. S4, indicating that the estimator in the TP strategy saturates the Cramér-Rao bound.

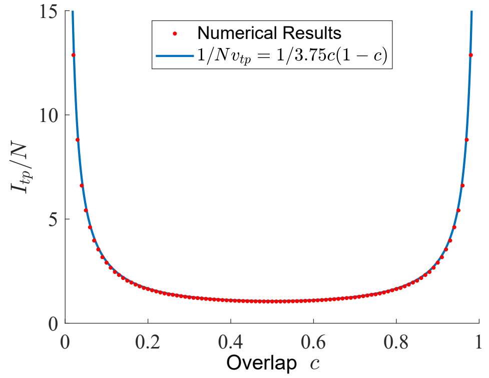

FIG. S4. Fisher information of overlap in Tomography-Projection strategy. Numerical results (red dots) are given by calculating the unit FI I_{tp}/N with $N = 900$. The inverse of scaled average variance (blue line) in TP strategy is derived form Eq. (S51).

E. Numerical results of average variance for TT and TP strategies

In this section, we show the numerical verification of the theoretical results of average overlap estimation variance in TT and TP strategies. In TP strategy, for a given reconstructed state $|\tilde{\phi}\rangle$, the probability of recording k successful

projection out of N measurements is given by

$$P_{proj}(k|\tilde{\phi}, N) = \binom{N}{k}(|\langle\tilde{\phi}|\psi\rangle|^2)^k(1 - |\langle\tilde{\phi}|\psi\rangle|^2)^{N-k}. \tag{S54}$$

Under a specific choice of U and φ, the expressions of overlap estimation variance for TT and TP strategy are given by

$$v_{tt}(c, N|U, \varphi) = \sum_{n_x^1, n_y^1, n_z^1=0}^{N/3} \sum_{n_x^2, n_y^2, n_z^2=0}^{N/3} \left(|\langle\tilde{\phi}|\tilde{\psi}\rangle|^2 - c\right)^2 \times P_{tomo}(\tilde{\phi}(n_x^1, n_y^1, n_z^1)|c, N, U, \varphi) \times P_{tomo}(\tilde{\psi}(n_x^2, n_y^2, n_z^2)|N, U), \tag{S55}$$

$$v_{tp}(c, N|U, \varphi) = \sum_{n_x, n_y, n_z=0}^{N/3} \left[\sum_{k=0}^{N}(\frac{k}{N} - c)^2 P_{proj}(k|\tilde{\phi}, N)\right] \times P_{tomo}(\tilde{\phi}(n_x, n_y, n_z)|c, N, U, \varphi), \tag{S56}$$

where P_{tomo} is defined in Eq. (S9). By averaging the variance with respect to U and φ and multiplying by the copy number N, the scaled average variance can be expressed as

$$Nv(c) = \frac{N}{2\pi}\int_U \int_0^{2\pi} v(c, N|U, \varphi) dU d\varphi, \tag{S57}$$

where $v(c, N|\varphi, U)$ is given by Eq. (S55) for TT strategy and Eq. (S56) for TP strategy. We employ Monte Carlo integration technique to numerically compute the integration in Eq. (S57) for both the TT and TP strategies. For a given overlap value and an estimation strategy, we randomly sample 1000 pairs of U and φ using the Haar measure, and then calculate the average variance as the integration value using these samples. As illustrated in Fig. S5, we select 41 overlap values for both TT and TP to calculate the scaled average variance with $N = 900$ copies. We obtain the numerical expressions of $Nv(c)$ by using $Nv(c) = \alpha c(1 - c) + \beta$ as the fitting formula. With a large N, the undetermined coefficient $\beta = O(1/N)$ term can be ignored.

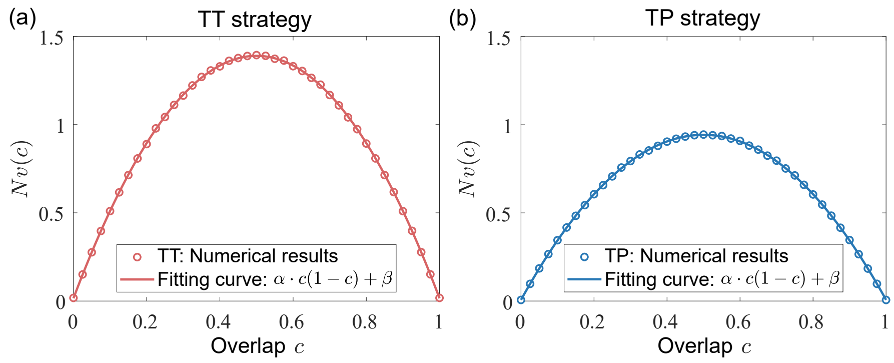

FIG. S5. Numerical calculation results and the fitting curves of the scaled average variance $Nv(c)$ in two local strategies. **a** Tomography-Tomography (TT) strategy. The fitting expression is $Nv(c) = 5.496c(1-c) + 0.016$ with the 95% confidence bound of α as $(5.484, 5.507)$. **b** Tomography-Projection (TP) strategy. The fitting expression is $Nv(c) = 3.746c(1-c) + 0.006$ with the 95% confidence bound of α as $(3.737, 3.754)$. The coefficients of determination R^2 for both fittings are 1.000.

F. TT and TP strategies for high-dimensional quantum states

The analysis of the precision of TT and TP strategies for estimating the overlap of two unknown qubits can be generalized to high-dimensional quantum states. As discussed in Section I, without loss of generality, we can express two d-dimensional quantum states (qudits) $|\psi\rangle$, $|\phi\rangle \in \mathbb{C}^d$ as:

$$\begin{aligned} |\psi\rangle &= U|0\rangle = UW\left(\sqrt{c}|0\rangle + \sqrt{1-c}\left|\phi_\perp^{r,0}\right\rangle\right) = \sqrt{c}|\phi\rangle + \sqrt{1-c}|\phi_\perp^r\rangle, \\ |\phi\rangle &= UW|0\rangle = U\left(\sqrt{c}|0\rangle + \sqrt{1-c}\left|\psi_\perp^{r,0}\right\rangle\right) = \sqrt{c}|\psi\rangle + \sqrt{1-c}|\psi_\perp^r\rangle, \end{aligned} \tag{S58}$$

where we assume a real-valued unsquared overlap $\langle\psi|\phi\rangle = \langle 0|W|0\rangle = \sqrt{c}$. Here, $|\psi_\perp^r\rangle$ and $|\phi_\perp^r\rangle$ are orthogonal to $|\psi\rangle$ and $|\phi\rangle$, respectively, and satisfy $\langle\phi_\perp^r|\psi_\perp^r\rangle = -\sqrt{c}$. Both $|\psi_\perp^{r,0}\rangle$ and $|\phi_\perp^{r,0}\rangle$ are orthogonal to $|0\rangle$.

We consider a sufficient-copy scenario for tomography ($N \gg d$), ensuring the tomography fidelity approaching 1. In this regime, a general tomography approach reconstructs a qudit with an average fidelity of $\overline{F} = 1 - \kappa/N + O(1/N^2)$. Our analysis focuses on the leading-order term, κ/N, which dominates the average infidelity for large N. Similar to Eq. (S19), the reconstructed states for $|\psi\rangle$ and $|\phi\rangle$, along with their corresponding probability distributions, can be expressed as

$$\begin{aligned} |\tilde{\psi}\rangle &= \cos\frac{\chi_1}{2}|\psi\rangle + \sin\frac{\chi_1}{2}|\psi_\perp^t\rangle = U\left(\cos\frac{\chi_1}{2}|0\rangle + \sin\frac{\chi_1}{2}|\psi_\perp^{t,0}\rangle\right), \quad p(\chi_1, |\psi_\perp^t\rangle\,|N, U), \\ |\tilde{\phi}\rangle &= \cos\frac{\chi_2}{2}|\phi\rangle + \sin\frac{\chi_2}{2}|\phi_\perp^t\rangle = UW\left(\cos\frac{\chi_2}{2}|0\rangle + \sin\frac{\chi_2}{2}|\phi_\perp^{t,0}\rangle\right), \quad p(\chi_2, |\phi_\perp^t\rangle\,|N, UW), \end{aligned} \tag{S59}$$

where $|\psi_\perp^t\rangle$ and $|\phi_\perp^t\rangle$ are orthogonal to $|\psi\rangle$ and $|\phi\rangle$, respectively, residing within two $(d-1)$-dimensional subspaces. The average fidelity of tomography implies $\overline{\langle\chi_1^2\rangle} = \overline{\langle\chi_2^2\rangle} = 4\kappa/N + O(1/N^2)$. Note that $|\psi_\perp^{r,0}\rangle$ from Eq. (S58) and $|\psi_\perp^{t,0}\rangle$ from Eq. (S59) both reside in the $(d-1)$-dimensional subspace orthogonal to $|0\rangle$, spanned by $\{|1\rangle, |2\rangle, .., |d-1\rangle\}$. We consider an approximation of the joint PDF of χ_1 and $|\psi_\perp^{t,0}\rangle$ after averaging over U. In the reconstructed state $|\tilde{\psi}\rangle$, the parameter χ_1 denotes the magnitude of tomography error, and $|\psi_\perp^t\rangle$ represents the error direction in the subspace orthogonal to $|\psi\rangle$. Averaging over the Haar-distributed of $|\psi\rangle$ makes the error direction isotropic and independent of the error magnitude. Therefore, for a sufficiently large number of copies N used for tomography, we approximate the distribution of $|\psi_\perp^{t,0}\rangle = U^\dagger |\psi_\perp^t\rangle$ as Haar distributed in the $(d-1)$-dimensional subspace orthogonal to $|0\rangle$, which means $\int_U p(\chi_1, |\psi_\perp^t\rangle\,|N, U)dU \approx p_\chi(\chi_1|N)$. Then, suppose there is a function with the form $f(\chi_1, \langle\psi_\perp^r|\psi_\perp^t\rangle) = f_1(\chi_1)f_2(\langle\psi_\perp^r|\psi_\perp^t\rangle)$, the average of $f(\chi_1, \langle\psi_\perp^r|\psi_\perp^t\rangle)$ can be shown as

$$\overline{\langle f_1(\chi_1)f_2(\langle\psi_\perp^r|\psi_\perp^t\rangle)\rangle} \approx \overline{\langle f_1(\chi_1)\rangle} \cdot \overline{\left\langle f_2(\langle\psi_\perp^{r,0}|\psi_\perp^{t,0}\rangle)\right\rangle}. \tag{S60}$$

For example, the following holds:

$$\begin{aligned} \overline{\left\langle \chi_1^2 \left|\langle\psi_\perp^r|\psi_\perp^t\rangle\right|^2 \right\rangle} &= \int_U \int_{\chi_1} \int_{\psi_\perp^t} \chi_1^2 \left|\langle\psi_\perp^r|\psi_\perp^t\rangle\right|^2 p\left(\chi_1, |\psi_\perp^t\rangle\,|N, U\right) dU d\chi_1 d\psi_\perp^t, \\ &= \int_{\chi_1} \int_{\psi_\perp^{t,0} \in \mathbb{C}^{d-1}} \chi_1^2 \left|\langle\psi_\perp^{r,0}|\psi_\perp^{t,0}\rangle\right|^2 \left(\int_U p(\chi_1, U|\psi_\perp^{t,0}\rangle\,|N, U)dU\right) d\chi_1 d\psi_\perp^{t,0}, \\ &\approx \int_{\chi_1} \chi_1^2 p_\chi(\chi_1|N)d\chi_1 \int_{\psi_\perp^{t,0} \in \mathbb{C}^{d-1}} \left|\langle\psi_\perp^{r,0}|\psi_\perp^{t,0}\rangle\right|^2 d\psi_\perp^{t,0} \\ &= \overline{\langle\chi_1^2\rangle}\,\overline{\left|\langle\psi_\perp^{r,0}|\psi_\perp^{t,0}\rangle\right|^2}, \end{aligned} \tag{S61}$$

where $d\psi_\perp^t$ and $d\psi_\perp^{t,0}$ denote the Haar measure. We use $\langle\psi_\perp^r|\psi_\perp^t\rangle = \langle\psi_\perp^{r,0}|\psi_\perp^{t,0}\rangle$ and approximate the joint PDF of χ_1 and $|\psi_\perp^{t,0}\rangle$, noting that $\langle\psi_\perp^{r,0}|\psi_\perp^{t,0}\rangle$ is independent of U. We further consider the average inner product between $|\psi_\perp^{r,0}\rangle$ and $|\psi_\perp^{t,0}\rangle$ as

$$\begin{aligned} \overline{\langle\psi_\perp^{r,0}|\psi_\perp^{t,0}\rangle} &= \int_{\psi_\perp^{t,0} \in \mathbb{C}^{d-1}} \left(\int_\omega \langle\psi_\perp^{r,0}|\psi_\perp^{t,0}\rangle\, dW\right) \left(\int_U \int_{\chi_1} p(\chi_1, U|\psi_\perp^{t,0}\rangle\,|N, U)dU d\chi_1\right) d\psi_\perp^{t,0}, \\ &\approx \int_{\psi_\perp^{t,0} \in \mathbb{C}^{d-1}} \left(\int_\omega \langle\psi_\perp^{r,0}|\psi_\perp^{t,0}\rangle\, dW\right) d\psi_\perp^{t,0} \\ &= \int_{\psi_\perp^{r,0} \in \mathbb{C}^{d-1}} \int_{\psi_\perp^{t,0} \in \mathbb{C}^{d-1}} \langle\psi_\perp^{r,0}|\psi_\perp^{t,0}\rangle\, d\psi_\perp^{r,0} d\psi_\perp^{t,0} \\ &= 0, \end{aligned} \tag{S62}$$

where $d\psi_\perp^{r,0}$ and $d\psi_\perp^{t,0}$ denote the Haar measure. The second equality in Eq. (S62) arises because $|\psi_\perp^{r,0}\rangle$ depends only on W subject to the constraints $\langle 0|W|0\rangle = \sqrt{c}$ and $\langle 0|\psi_\perp^{r,0}\rangle = 0$. Therefore, integrating over W (i.e., $\int_\omega \langle\psi_\perp^{r,0}|\psi_\perp^{t,0}\rangle\, dW$) is equivalent to integrating over the Haar-random $|\psi_\perp^{r,0}\rangle$ in the $(d-1)$-dimensional subspace. This result reflects the fact that the average inner product of two Haar-random, $(d-1)$-dimensional states is zero, i.e., $\overline{\langle\psi_\perp^{r,0}|\psi_\perp^{t,0}\rangle} = 0$. For the (squared) overlap between $|\psi_\perp^{r,0}\rangle$ and $|\psi_\perp^{t,0}\rangle$, we have

$$\overline{\left|\langle\psi_\perp^{r,0}|\psi_\perp^{t,0}\rangle\right|^2} \approx \int_{\psi_\perp^{t,0} \in \mathbb{C}^{d-1}} \int_{\psi_\perp^{r,0} \in \mathbb{C}^{d-1}} \left|\langle\psi_\perp^{r,0}|\psi_\perp^{t,0}\rangle\right|^2 d\psi_\perp^{r,0} d\psi_\perp^{t,0} = \frac{1}{d-1}, \tag{S63}$$

which represents the average overlap between two Haar-random states in the $(d-1)$-dimensional subspace orthogonal to $|0\rangle$ [6]. Due to the Haar measure, we can express $\langle\psi_\perp^{r,0}|\psi_\perp^{t,0}\rangle$ as $|\langle\psi_\perp^{r,0}|\psi_\perp^{t,0}\rangle|e^{i\zeta}$ where ζ is uniformly distributed. Consequently, $\overline{\left(\langle\psi_\perp^{r,0}|\psi_\perp^{t,0}\rangle\right)^2}=\overline{\left(\langle\psi_\perp^{t,0}|\psi_\perp^{r,0}\rangle\right)^2}=0$. Therefore, $\overline{(\mathrm{Re}[\langle\psi_\perp^{r,0}|\psi_\perp^{t,0}\rangle])^2}=1/2(d-1)$. Analogous results for χ_2, $\langle\phi_\perp^{r}|\phi_\perp^{t}\rangle$ and $\langle\phi_\perp^{r,0}|\phi_\perp^{t,0}\rangle$ can be obtained using the same approach. In summary, the following relationships hold:

$$\begin{aligned}\overline{\langle\psi_\perp^{r,0}|\psi_\perp^{t,0}\rangle}=\overline{\langle\phi_\perp^{r,0}|\phi_\perp^{t,0}\rangle}=\overline{\mathrm{Re}\left[\langle\psi_\perp^{r,0}|\psi_\perp^{t,0}\rangle\right]}=\overline{\mathrm{Re}\left[\langle\phi_\perp^{r,0}|\phi_\perp^{t,0}\rangle\right]}=0,\\ \overline{\left|\langle\psi_\perp^{r,0}|\psi_\perp^{t,0}\rangle\right|^2}=\overline{\left|\langle\phi_\perp^{r,0}|\phi_\perp^{t,0}\rangle\right|^2}=2\overline{\left(\mathrm{Re}\left[\langle\psi_\perp^{r,0}|\psi_\perp^{t,0}\rangle\right]\right)^2}=2\overline{\left(\mathrm{Re}\left[\langle\phi_\perp^{r,0}|\phi_\perp^{t,0}\rangle\right]\right)^2}=\frac{1}{d-1}.\end{aligned} \tag{S64}$$

For the TT strategy, the overlap estimator is given by

$$\begin{aligned}\tilde{c}_{tt}&=|\langle\tilde{\psi}|\tilde{\phi}\rangle|^2\\&=\left|\cos\frac{\chi_1}{2}\cos\frac{\chi_2}{2}\langle\psi|\phi\rangle+\cos\frac{\chi_1}{2}\sin\frac{\chi_2}{2}\langle\psi|\phi_\perp^t\rangle+\sin\frac{\chi_1}{2}\cos\frac{\chi_2}{2}\langle\psi_\perp^t|\phi\rangle+\sin\frac{\chi_1}{2}\sin\frac{\chi_2}{2}\langle\psi_\perp^t|\phi_\perp^t\rangle\right|^2\\&=\left|\cos\frac{\chi_1}{2}\cos\frac{\chi_2}{2}\sqrt{c}+\cos\frac{\chi_1}{2}\sin\frac{\chi_2}{2}\sqrt{1-c}\,\langle\phi_\perp^r|\phi_\perp^t\rangle+\sin\frac{\chi_1}{2}\cos\frac{\chi_2}{2}\sqrt{1-c}\,\langle\psi_\perp^t|\psi_\perp^r\rangle+\sin\frac{\chi_1}{2}\sin\frac{\chi_2}{2}\langle\psi_\perp^t|\phi_\perp^t\rangle\right|^2\\&\approx\left|\left(1-\frac{\chi_1^2+\chi_2^2}{8}\right)\sqrt{c}+\frac{\sqrt{1-c}}{2}\left(\chi_2\langle\phi_\perp^r|\phi_\perp^t\rangle+\chi_1\langle\psi_\perp^t|\psi_\perp^r\rangle\right)+\frac{\chi_1\chi_2}{4}\langle\psi_\perp^t|\phi_\perp^t\rangle\right|^2\\&\approx\left(1-\frac{\chi_1^2+\chi_2^2}{4}\right)c+\left(\chi_1\mathrm{Re}[\langle\psi_\perp^r|\psi_\perp^t\rangle]+\chi_2\mathrm{Re}[\langle\phi_\perp^r|\phi_\perp^t\rangle]\right)\sqrt{c(1-c)}+\left(\chi_1^2|\langle\psi_\perp^r|\psi_\perp^t\rangle|^2+\chi_2^2|\langle\phi_\perp^r|\phi_\perp^t\rangle|^2\right)\frac{1-c}{4}\\&\quad+\chi_1\chi_2\mathrm{Re}\left[\langle\psi_\perp^t|\psi_\perp^r\rangle\langle\phi_\perp^r|\phi_\perp^t\rangle\right]\frac{1-c}{2}+\frac{\chi_1\chi_2}{2}\mathrm{Re}\left[\langle\psi_\perp^t|\phi_\perp^t\rangle\right]\sqrt{c},\end{aligned} \tag{S65}$$

where terms higher than second order in χ_i and χ_j have been neglected. We further assume that $|\psi_\perp^{t,0}\rangle$ and $|\phi_\perp^{t,0}\rangle$ as independent and identically distributed, meaning $\int_U p(\chi_1,\psi_\perp^t|N,U)p(\chi_2,\phi_\perp^t|N,UW)dU\approx p_\chi(\chi_1|N)p_\chi(\chi_2|N)$. We expect the approximation applied to the probability distribution to introduce only higher-order errors, scaling as $O(1/N^2)$. Consequently, we can derive that $\overline{\langle\psi_\perp^t|\psi_\perp^r\rangle\langle\phi_\perp^r|\phi_\perp^t\rangle}=0$ and $\overline{\langle\psi_\perp^t|\phi_\perp^t\rangle}=0$. The average mean of the TT strategy is then given by:

$$\overline{\langle\tilde{c}_{tt}\rangle}=c-\frac{dc-1}{4(d-1)}\left(\overline{\langle\chi_1^2\rangle}+\overline{\langle\chi_2^2\rangle}\right)+O(\frac{1}{N^2})=c-\frac{2\kappa(dc-1)}{N(d-1)}+O(\frac{1}{N^2}), \tag{S66}$$

which is the general form of Eq. (S40). Considering only the first-order terms of χ_1 and χ_2 in Eq. (S65), together with Eq. (S64), the average variance of the TT strategy is:

$$\begin{aligned}v_{tt}=\overline{\langle(\tilde{c}_{tt}-c)^2\rangle}&=c(1-c)\overline{(\chi_1\mathrm{Re}[\langle\psi_\perp^r|\psi_\perp^t\rangle]+\chi_2\mathrm{Re}[\langle\phi_\perp^r|\phi_\perp^t\rangle])^2}+O(\frac{1}{N^2})\\&=c(1-c)\left(\overline{\langle\chi_1^2\rangle}\overline{\left(\mathrm{Re}\left[\langle\psi_\perp^{r,0}|\psi_\perp^{t,0}\rangle\right]\right)^2}+\overline{\langle\chi_2^2\rangle}\overline{\left(\mathrm{Re}\left[\langle\phi_\perp^{r,0}|\phi_\perp^{t,0}\rangle\right]\right)^2}\right)+O(\frac{1}{N^2})\\&=\frac{4\kappa c(1-c)}{(d-1)N}+O(\frac{1}{N^2}).\end{aligned} \tag{S67}$$

For $d=2$, this reduces to the qubit case in Eq. (S42).

For the TP strategy, the success projection probability is given by

$$\begin{aligned}p_{tp}=|\langle\psi|\tilde{\phi}\rangle|^2&=\left|\cos\frac{\chi_2}{2}\sqrt{c}+\sin\frac{\chi_2}{2}\sqrt{1-c}\,\langle\phi_\perp^r|\phi_\perp^t\rangle\right|^2\\&=\cos^2\frac{\chi_2}{2}\cdot c+\sin\chi_2\mathrm{Re}\left[\langle\phi_\perp^r|\phi_\perp^t\rangle\right]\sqrt{c(1-c)}+\sin^2\frac{\chi_2}{2}(1-c)\left|\langle\phi_\perp^r|\phi_\perp^t\rangle\right|^2\\&\approx c+\chi_2\mathrm{Re}\left[\langle\phi_\perp^r|\phi_\perp^t\rangle\right]\sqrt{c(1-c)}-\frac{\chi_2^2}{4}\left[\left(\left|\langle\phi_\perp^r|\phi_\perp^t\rangle\right|^2+1\right)c-\left|\langle\phi_\perp^r|\phi_\perp^t\rangle\right|^2\right],\end{aligned} \tag{S68}$$

which is the general form of Eq. (S46). The average mean of the TP strategy is

$$\overline{\langle\tilde{c}_{tp}\rangle}=\overline{\langle p_{tp}\rangle}=c-\frac{\overline{\langle\chi_2^2\rangle}}{4}\frac{dc-1}{d-1}+O(\frac{1}{N^2})=c-\frac{\kappa(dc-1)}{N(d-1)}+O(\frac{1}{N^2}). \tag{S69}$$

Similar to the qubit case, the average variance of the TP strategy can be decomposed into two parts:

$$\overline{\langle p_{tp}(1-p_{tp})\rangle} = c(1-c) + O(\frac{1}{N}),$$
$$\overline{\langle (p_{tp}-c)^2\rangle} = c(1-c)\overline{\langle \chi_2^2\rangle}\overline{\left(\text{Re}\left[\langle \phi_\perp^{r,0}|\phi_\perp^{t,0}\rangle\right]\right)^2} + O(\frac{1}{N^2}) = \frac{2\kappa c(1-c)}{(d-1)N} + O(\frac{1}{N^2}). \tag{S70}$$

The average variance for TP strategy is then

$$v_{tp} = \overline{\left\langle \frac{p_{tp}(1-p_{tp})}{N}\right\rangle} + \overline{\langle (p_{tp}-c)^2\rangle} = \left(\frac{2\kappa}{d-1}+1\right)\frac{c(1-c)}{N} + O(\frac{1}{N^2}). \tag{S71}$$

For $d=2$, this reduces to the qubit case in Eq. (S51).

The overlap estimation errors for the TT and TP strategies can be also decomposed into two components: a tomography error (v_{tomo}) and a projection error (v_{proj}):

$$v_{tomo} = \frac{2\kappa c(1-c)}{(d-1)N}, \quad v_{proj} = \frac{c(1-c)}{N}. \tag{S72}$$

Note that v_{tomo} includes a factor $1/(d-1)$, while v_{proj} remains the same as in the single-qubit case. Similarly, the average variances can then be expressed as $v_{tt} = 2v_{tomo}$ and $v_{tp} = v_{tomo} + v_{proj}$.

G. TT and TP strategies performance with high-dimensional state tomography

Here, we analyze the scaled average infidelity κ of high-dimensional quantum states using various measurement approaches in the sufficient-copy scenario. Consider the tomography of a d-dimensional pure quantum state $|\psi\rangle$ using N copies. Therefore, the total quantum state is $|\psi\rangle^{\otimes N}$. We examine three categories of tomography measurements:

Joint measurements across all copies. The first category involves joint measurements on $|\psi\rangle^{\otimes N}$. The optimal measurement approach [7] achieves an average fidelity of $\overline{F} = (N+1)/(N+d)$ [8]. Therefore, the optimal scaled average infidelity is $\kappa_{opt} = d-1$. Applying this optimal tomography to the TT and TP strategies for overlap estimation effectively yields the *estimate-and-estimate* and *estimate-and-project* strategies described in [1], with $v_{tt} = 4c(1-c)/N$ and $v_{tp} = 3c(1-c)/N$, respectively.

Independent measurements on each copy. The second category allows arbitrary measurements within each copy of $|\psi\rangle$, but restricts measurements to be independent across copies. For a quantum state ρ of rank at most r, using only independent, non-adaptive measurements on each copy, the optimal tomography approach achieves a sample complexity of $N = O(dr^2/\epsilon^2)$ to estimate ρ within trace distance ϵ [9, 10], where trace distance is defined as $T(\rho,\tilde{\rho}) = \text{tr}|\rho-\tilde{\rho}|/2$. For pure states ($r=1$, $\rho = |\psi\rangle\langle\psi|$), the estimation $\tilde{\rho}$ from the approach in [9] is generally not exact rank-1. A rank-1 estimate $|\tilde{\psi}\rangle\langle\tilde{\psi}|$ can be constructed from the eigenvector $|\tilde{\psi}\rangle$ corresponding to the largest eigenvalue of $\tilde{\tilde{\rho}}$. Given $T(\rho,\tilde{\rho}) \le \epsilon$, let the sorted eigenvalues of ρ and $\tilde{\rho}$ be $(1,0,\ldots,0)$ and $(\lambda_1,\lambda_2,\ldots,\lambda_d)$ with $\lambda_1 \ge \lambda_2 \ge \cdots \ge \lambda_d$, respectively. Weyl's inequality implies $|1-\lambda_1| \le \|\tilde{\rho}-\rho\|_2 \le \text{tr}|\rho-\tilde{\rho}| \le 2\epsilon$ and $\max_{j\neq 1}\{|\lambda_j|\} \le 2\epsilon$. Applying the Davis-Kahan ($\sin\theta$) theorem [11] with a spectral gap $\delta \ge 1-2\epsilon$, the infidelity between $|\psi\rangle$ and $|\tilde{\psi}\rangle$ is bounded by:

$$\sqrt{1-|\langle\psi|\tilde{\psi}\rangle|^2} \le \frac{\|\tilde{\rho}-\rho\|_2}{\delta} \le \frac{2\epsilon}{1-2\epsilon} \approx 2\epsilon = O\left(\sqrt{\frac{d}{N}}\right). \tag{S73}$$

Therefore, post-processing $\tilde{\rho}$ to obtain $|\tilde{\psi}\rangle$ introduces at most a constant factor of 2 in the error. Consequently, $1-|\langle\psi|\tilde{\psi}\rangle|^2 \le 4\epsilon^2 = O(d/N) \sim \kappa_{ind}/N$, where $\kappa_{ind} = O(d)$ denotes the optimal scaled infidelity for tomography using independent measurements. Combining this into the TT and TP strategies, yields the following average variances:

$$v_{tt} = \frac{O(d)\cdot 4c(1-c)}{(d-1)N} \sim O\left(\frac{c(1-c)}{N}\right), \quad v_{tp} = \left(2\frac{O(d)}{d-1}+1\right)\frac{c(1-c)}{N} \sim O\left(\frac{c(1-c)}{N}\right). \tag{S74}$$

These average variances become independent for $d \gg 1$, similar to the optimal joint measurement approach in the first category. This stems from the same $N \sim O(d/\epsilon^2)$ scaling of the sample complexity for tomography of rank-1 states, regardless of whether joint or independent measurements are used [10, 12].

Local measurements on each qubit of multi-qubit states. Now we consider the situation that the d-dimensional quantum state is composed of n qubits ($d = 2^n$). If one can perform a joint measurement on all qubits of each copy, it reduces to the second approach discussed above. Here we consider a more practical tomography approach involving only local measurements on each qubit of each copy of the n-qubit state, such as Pauli measurements [13, 14]. In [13], the authors show that using $N = O(r^2 d^2 \log d/\epsilon^2) = O(r^2 4^n n/\epsilon^2)$ copies allows reconstruction of a rank-r ρ with trace distance less than ϵ. Thus, for pure state tomography using local measurements, the scaled infidelity is given by $\kappa_{loc} = O(4^n n)$. The average variances for the TT and TP strategies under this restriction are:

$$
\begin{aligned}
v_{tt} &= \frac{O(4^n n) \cdot 4c(1-c)}{(2^n - 1)N} \sim O\left(\frac{2^n nc(1-c)}{N}\right), \\
v_{tp} &= \left(2\frac{O(4^n n)}{2^n - 1} + 1\right)\frac{c(1-c)}{N} \sim O\left(\frac{2^n nc(1-c)}{N}\right),
\end{aligned}
\tag{S75}
$$

where the approximations hold when $d \gg 1$. However, the projective measurement on $|\tilde{\psi}\rangle$ in the TP strategy is generally non-local. Therefore, restricting to local operations effectively precludes the TP strategy.

Table I summarizes the results of average variances. We emphasize that these results are derived asymptotically under the sufficient-copy assumption ($N \gg d$). This assumption arises from the requirement of a sufficiently small ϵ in our average variance analysis, combined with the tomography sample complexity scaling of $N \sim O(d/\epsilon^2)$ or $O(4^n n/\epsilon^2)$. In practice, implementing joint measurements across all copies becomes significantly challenging as N increases. Independent measurements on each copy mitigate this issue and also yield dimension-independent average variances for TT and TP, as shown in Eq. (S74), although implementation challenges persist for large d. Restricting measurements to local operations explicitly introduces dimension-dependent average variances and generally renders the TP strategy impractical.

	Joint measurements	Independent measurements	Local measurements
κ	$d-1$	$O(d)$	$O(4^n n)$
v_{tt}	$4c(1-c)/N$	$O(c(1-c)/N)$	$O(2^n nc(1-c)/N)$
v_{tp}	$3c(1-c)/N$	$O(c(1-c)/N)$	$O(2^n nc(1-c)/N)$

TABLE I. The scaled average infidelity κ, and average variances v_{tt} and v_{tp} for the TT and TP strategies with different tomography approaches for high-dimensional quantum states in the sufficient-copy scenario ($N \gg d$). For local measurements, we consider each copy is an n-qubit state, therefore $d = 2^n$.

In the following, we consider the limited-copy scenario, where $N = \alpha d$ with $\alpha \sim O(1)$. We focus on the regime where $d \gg 1$, making this scenario common. In this case, qudit tomography suffers from both information incompleteness and significant statistical errors, resulting in poor and biased estimations. Therefore, the overlap estimators for the TT and TP strategies are generally biased. Following the supplemental material of [1], which derives the mean square error (MSE) for *estimate-and-project* (Eq. (106) in [1]) and *estimate-and-estimate* (Eq. (78) in [1]) strategies, we can calculate MSE for the TT strategy with the optimal joint measurement tomography as follows:

$$
\begin{aligned}
v_{tt}(c, N, d) &= \frac{(2N+d)[(c(cd-2)(d+1)+2)(2N+d+1) + 2c(1-c)N^2]}{(N+d)^2(N+d+1)^2} \\
&= \frac{(2\alpha+1)d[(c(cd-2)(d+1)+2)(2\alpha d+d+1) + 2c(1-c)\alpha^2 d^2]}{(\alpha+1)^2 d^2(\alpha d+d+1)^2} \\
&= \frac{(2\alpha+1)[c^2(2\alpha+1)d + 2c(1-c)\alpha^2]}{(\alpha+1)^4 d} + O\left(\frac{1}{\alpha^2 d^2}\right) \\
&= \frac{(2\alpha+1)^2}{(\alpha+1)^4}c^2 + \frac{2(2\alpha+1)\alpha^2}{(\alpha+1)^4 d}c(1-c) + O\left(\frac{1}{\alpha^2 d^2}\right) \\
&= \frac{(2\alpha+1)^2}{(\alpha+1)^4}c^2 + \frac{2(2\alpha+1)\alpha^3}{(\alpha+1)^4 N}c(1-c) + O\left(\frac{1}{N^2}\right).
\end{aligned}
\tag{S76}
$$

Since $\alpha \sim O(1)$, the term $(2\alpha+1)^2 c^2/(\alpha+1)^4$ introduces a constant-order error in the overlap estimation, leading to an MSE of $v_{tt} = O(1)$ for the TT strategy and demonstrating its inefficiency in the limited-copy scenario. To recover the sufficient-copy scenario, where the first term is negligible compared to the $O(1/N)$ terms, it requires $1/\alpha^2 \sim o(1/N)$, or equivalently, $N \sim \omega(d^2)$. In this case, the constant term vanishes, and the second term dominates,

yielding $v_{tt} = 4c(1-c)/N$, consistent with our previous discussion. Similarly, the MSE for the TP strategy with the optimal joint measurement tomography is:

$$\begin{aligned}
v_{tp}(c,N,d) &= \frac{c^2(d^2N + dN - 3N^2 + N) + c(3N^2 - dN - 5N) + 3N + d - 1}{N(N+d)(N+d+1)} \\
&= \frac{c^2(\alpha d^2 + \alpha d - 3\alpha^2 d + \alpha)d + c(3\alpha^2 d^2 - \alpha d^2 - 5\alpha d) + 3\alpha d + d - 1}{\alpha(\alpha+1)(\alpha d + d + 1)d^2} \\
&= \frac{c^2 d + c(3\alpha - 1)}{(\alpha+1)^2 d} + \frac{c^2(1-3\alpha)}{(\alpha+1)^2 d} + O\left(\frac{1}{\alpha^2 d^2}\right) \\
&= \frac{c^2}{(\alpha+1)^2} + \frac{3\alpha - 1}{(\alpha+1)^2 d} c(1-c) + O\left(\frac{1}{\alpha^2 d^2}\right) \\
&= \frac{c^2}{(\alpha+1)^2} + \frac{\alpha(3\alpha - 1)}{(\alpha+1)^2 N} c(1-c) + O\left(\frac{1}{N^2}\right).
\end{aligned} \tag{S77}$$

Again, the first term represents an $O(1)$ error when $\alpha \sim O(1)$. When $\alpha \gg 1$, it becomes negligible, and the TP strategy becomes asymptotically unbiased, with an MSE consistent with the average variance $v_{tp} = 3c(1-c)/N$. Therefore, due to the limitations of tomography in the limited-copy scenario, both TT and TP strategies provide biased overlap estimations with mean square errors scaling as $O(1)$.

III. OPTICAL SWAP TEST WITH EXPERIMENTAL IMPERFECTIONS

A. Ideal optical swap test

The optical swap test (OST) is a modified version of the swap test that can be implemented practically via a multi-mode Hong-Ou-Mandel interference (HOMI), which uses a non-polarizing beam-splitter (NPBS) to perform the interference between two photons encoded by $|\psi\rangle$ and $|\phi\rangle$, respectively. For the overlap estimation task, we can utilize the OST to estimate the overlap by recording the test results as either "pass" or "fail" over N trials. As illustrated in Fig. S6, we consider an ideal case that two perfect indistinguishable photons have been prepared as a two-qudit joint state, which can be expressed as

$$|\psi\rangle_1 \otimes |\phi\rangle_2 = (\sum_{i=1}^{d} \alpha_i \hat{a}_{i,1}^\dagger |0\rangle_1) \otimes (\sum_{j=1}^{d} \beta_j \hat{a}_{j,2}^\dagger |0\rangle_2), \tag{S78}$$

here $\hat{a}_{i,1}^\dagger$ and $\hat{a}_{j,2}^\dagger$ denote photon creation operator in i mode at input port 1 of NPBS and j mode at input port 2 respectively, $|0\rangle_1$ and $|0\rangle_2$ represent the vacuum state for two input sides. The balanced NPBS has following transformation on creation operators of input modes: $\hat{a}_{i,1}^\dagger \to (\hat{a}_{i,3}^\dagger + \hat{a}_{i,4}^\dagger)/\sqrt{2}$, $\hat{a}_{j,2}^\dagger \to (\hat{a}_{j,3}^\dagger - \hat{a}_{j,4}^\dagger)/\sqrt{2}$, where 3 and 4 denote two output ports. The output field can be written as

$$\begin{aligned}
|\Psi^{\text{out}}\rangle &= \frac{1}{2}\sum_{i=1}^{d}\sum_{j=1}^{d} \alpha_i \beta_j (\hat{a}_{i,3}^\dagger + \hat{a}_{i,4}^\dagger)(\hat{a}_{j,3}^\dagger - \hat{a}_{j,4}^\dagger) |0\rangle_3 |0\rangle_4 \\
&= \sum_{i=1}^{d} \frac{\alpha_i \beta_i}{\sqrt{2}} (|2_i\rangle_3 |0\rangle_4 - |0\rangle_3 |2_i\rangle_4) + \sum_{1 \le i < j \le d} \frac{\alpha_i \beta_j + \alpha_j \beta_i}{2} (|1_i 1_j\rangle_3 |0\rangle_4 - |0\rangle_3 |1_i 1_j\rangle_4) \\
&\quad + \sum_{i \neq j}^{d} \frac{\alpha_i \beta_j - \alpha_j \beta_i}{2} |1_i\rangle_3 |1_j\rangle_4 ,
\end{aligned} \tag{S79}$$

where $|1_i 1_j\rangle_3$ or $|1_i 1_j\rangle_4$ describe two photons occupying the same output port 3 or 4, but in different mode i and j. After the interference, photon detectors are used to detect the photon distribution of the output filed. In the OST, "pass" outcomes correspond to cases where both photons are detected in the same output port of the interference NPBS, such as $|1_i 1_j\rangle_3 |0\rangle_4$ and $|2_i\rangle_3 |0\rangle_4$. Conversely, cases where the photons are detected in different output ports

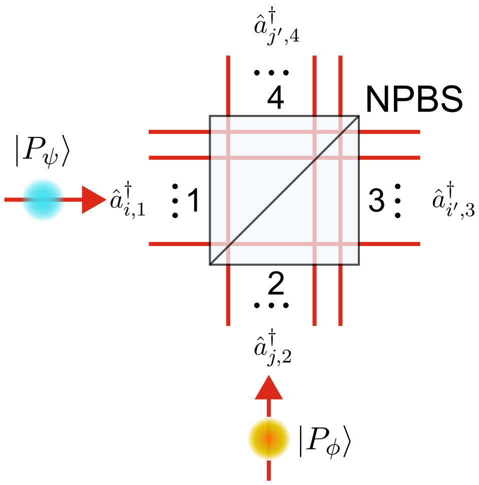

FIG. S6. Schematic of multimode Hong-Ou-Mandel interference. The non-polarizing beam splitter (NPBS) has two input ports (1 and 2) and two output ports (3 and 4). $|P_\psi\rangle$ and $|P_\phi\rangle$ represent the quantum states of the photon encoded by the qudits $|\psi\rangle$ and $|\phi\rangle$, respectively. The photons are distributed among d discrete modes, such as path modes and polarization modes. The creation operators for the input ports 1 and 2 are denoted as $\hat{a}^\dagger_{i,1}$ and $\hat{a}^\dagger_{j,2}$, while for the output ports 3 and 4, they are represented as $\hat{a}^\dagger_{i',3}$ and $\hat{a}^\dagger_{j',4}$.

like $|1_i\rangle_3 |1_j\rangle_4$, denote the "fail" outcomes. From Eq. (S79), the probability of "fail" outcomes for OST is given by

$$\begin{aligned} p(f) &= \sum_{i\neq j}^{d} \left|\frac{\alpha_i\beta_j - \alpha_j\beta_i}{2}\right|^2 = \sum_{i=1,j=1}^{d} \left|\frac{\alpha_i\beta_j - \alpha_j\beta_i}{2}\right|^2 \\ &= \frac{1}{2}\sum_{i=1,j=1}^{d} |\alpha_i|^2|\beta_j|^2 - \frac{1}{2}(\sum_{i=1}^{d}\alpha_i\beta_i^*)(\sum_{j=1}^{d}\alpha_j^*\beta_j) \\ &= \frac{1}{2}(1 - |\langle\psi|\phi\rangle|^2), \end{aligned} \tag{S80}$$

with the (unsquared) overlap $\langle\psi|\phi\rangle = \sum_j \alpha_j^*\beta_j$ and the normalization conditions $\sum_i |\alpha_i|^2 = \sum_i |\beta_i|^2 = 1$. Hence, the overlap between two states determines the probability of "fail" outcomes in the OST. After N trials of OST, if we faithfully record the counts of "pass" and "fail" outcomes, we can use the "fail" counts k_f to estimate the overlap as $\tilde{c}_{ost} = 1 - 2k_f/N$. The overlap estimation variance through the ideal OST strategy is then given by $v(c,N) = (1-c^2)/N$, where $c = |\langle\psi|\phi\rangle|^2$ is the true overlap.

B. OST with partially distinguishable photons and non-balanced beam-splitter

In a HOMI experiment, photon pairs generated by spontaneous parametric down conversion are usually not perfectly indistinguishable due to slightly different spectral mode or spatial mode. In this section, we consider the case that two photons with discrete modes being encoded as $|\psi\rangle$ and $|\phi\rangle$, and have different properties on their non-encoded modes where we regard them as internal modes. The spectral mode in the internal modes is mainly considered. To begin with, we describe these photons as follows:

$$|P_\psi\rangle = \int d\omega S_1(\omega)\sum_{i=1}^{d}\alpha_i\hat{a}_i^\dagger(\omega)|0\rangle\,,\ |P_\phi\rangle = \int d\omega S_2(\omega)\sum_{j=1}^{d}\beta_j\hat{a}_j^\dagger(\omega)|0\rangle\,, \tag{S81}$$

here $|P_\psi\rangle$ and $|P_\phi\rangle$ denote the quantum states of the photons whose discrete modes are encoded by $|\psi\rangle$ and $|\phi\rangle$, respectively. $S_1(\omega)$ and $S_2(\omega)$ represent the spectral amplitudes of two photons, which may be not identical. The input state on ports 1 and 2 of the NPBS is then given by

$$|\Phi^{\text{in}}\rangle = |P_\psi\rangle_1 |P_\phi\rangle_2 = \int d\omega_1 \int d\omega_2 S_1(\omega_1)S_2(\omega_2)\sum_{i,j=1}^{d}\alpha_i\beta_j\hat{a}^\dagger_{i,1}(\omega_1)\hat{a}^\dagger_{j,2}(\omega_2)|0\rangle_1|0\rangle_2\,. \tag{S82}$$

We consider that the NPBS in experiment is not perfectly balanced, with reflectivity η. The non-balanced NPBS transforms the creation operators as follows: $\hat{a}^{\dagger}_{i,1}(\omega) \to \sqrt{1-\eta}\hat{a}^{\dagger}_{i,3}(\omega) + \sqrt{\eta}\hat{a}^{\dagger}_{i,4}(\omega)$, $\hat{a}^{\dagger}_{i,2}(\omega) \to \sqrt{\eta}\hat{a}^{\dagger}_{i,3}(\omega) - \sqrt{1-\eta}\hat{a}^{\dagger}_{i,4}(\omega)$. The output state on ports 3 and 4 is then given by

$$\begin{aligned}
|\Phi^{\text{out}}\rangle &= \sum_{i,j=1}^{d} \alpha_i \beta_j \int d\omega_1 \int d\omega_2 S_1(\omega_1) S_2(\omega_2) \left[\sqrt{1-\eta}\hat{a}^{\dagger}_{i,3}(\omega_1) + \sqrt{\eta}\hat{a}^{\dagger}_{i,4}(\omega_1)\right] \left[\sqrt{\eta}\hat{a}^{\dagger}_{j,3}(\omega_2) - \sqrt{1-\eta}\hat{a}^{\dagger}_{j,4}(\omega_2)\right] |0\rangle_3 |0\rangle_4 \\
&= \sum_{i,j=1}^{d} \alpha_i \beta_j \int d\omega_1 \int d\omega_2 S_1(\omega_1) S_2(\omega_2) \times \Big[\eta \hat{a}^{\dagger}_{i,4}(\omega_1)\hat{a}^{\dagger}_{j,3}(\omega_2) - (1-\eta)\hat{a}^{\dagger}_{i,3}(\omega_1)\hat{a}^{\dagger}_{j,4}(\omega_2) \\
&\quad + \sqrt{\eta(1-\eta)}\hat{a}^{\dagger}_{i,3}(\omega_1)\hat{a}^{\dagger}_{j,3}(\omega_2) - \sqrt{\eta(1-\eta)}\hat{a}^{\dagger}_{i,4}(\omega_1)\hat{a}^{\dagger}_{j,4}(\omega_2)\Big] |0\rangle_3 |0\rangle_4 ,
\end{aligned} \tag{S83}$$

where we assume that two photons arrived at NPBS simultaneously and time delay between two photons is zero. The projector of detecting one photon in mode m of port 3, and another one in mode n of port 4, which is corresponding to one possible "fail" outcome of the OST, is shown as

$$\hat{P}_{m,3} \otimes \hat{P}_{n,4} = \int d\omega_3 \hat{a}^{\dagger}_{m,3}(\omega_3) |0\rangle_3 \langle 0|_3 \hat{a}_{m,3}(\omega_3) \otimes \int d\omega_4 \hat{a}^{\dagger}_{n,4}(\omega_4) |0\rangle_4 \langle 0|_4 \hat{a}_{n,4}(\omega_4). \tag{S84}$$

We use $p(f,m,n)$ to denote the probability of detecting this kind of "fail" outcome, and it can be derived as follows:

$$\begin{aligned}
p(f,m,n) &= \langle \Psi^{\text{out}}| \hat{P}_{m,3} \otimes \hat{P}_{n,4} |\Psi^{\text{out}}\rangle \\
&= \sum_{i,j,k,l=1}^{d} \alpha_i^* \beta_j^* \alpha_k \beta_l \int d\omega_1 \int d\omega_2 \int d\omega_1^{'} \int d\omega_2^{'} \int d\omega_3 \int d\omega_4 S_1^*(\omega_1) S_2^*(\omega_2) S_1(\omega_1^{'}) S_2(\omega_2^{'}) \\
&\quad \times \langle 0|_3 \langle 0|_4 \left[\eta \hat{a}_{i,4}(\omega_1)\hat{a}_{j,3}(\omega_2)\hat{a}^{\dagger}_{m,3}(\omega_3)\hat{a}^{\dagger}_{n,4}(\omega_4) - (1-\eta)\hat{a}_{i,3}(\omega_1)\hat{a}_{j,4}(\omega_2)\hat{a}^{\dagger}_{m,3}(\omega_3)\hat{a}^{\dagger}_{n,4}(\omega_4)\right] |0\rangle_3 |0\rangle_4 \\
&\quad \times \langle 0|_3 \langle 0|_4 \left[\eta \hat{a}_{m,3}(\omega_3)\hat{a}_{n,4}(\omega_4)\hat{a}^{\dagger}_{k,4}(\omega_1^{'})\hat{a}^{\dagger}_{l,3}(\omega_2^{'}) - (1-\eta)\hat{a}_{m,3}(\omega_3)\hat{a}_{n,4}(\omega_4)\hat{a}^{\dagger}_{k,3}(\omega_1^{'})\hat{a}^{\dagger}_{l,4}(\omega_2^{'})\right] |0\rangle_3 |0\rangle_4 \\
&= \sum_{i,j,k,l=1}^{d} \alpha_i^* \beta_j^* \alpha_k \beta_l \int d\omega_1 \int d\omega_2 \int d\omega_1^{'} \int d\omega_2^{'} \int d\omega_3 \int d\omega_4 S_1^*(\omega_1) S_2^*(\omega_2) S_1(\omega_1^{'}) S_2(\omega_2^{'}) \\
&\quad \times (\eta\delta(\omega_1-\omega_4)\delta_{in}\delta(\omega_2-\omega_3)\delta_{jm} - (1-\eta)\delta(\omega_1-\omega_3)\delta_{im}\delta(\omega_2-\omega_4)\delta_{jn}) \\
&\quad \times (\eta\delta(\omega_1^{'}-\omega_4)\delta_{kn}\delta(\omega_2^{'}-\omega_3)\delta_{lm} - (1-\eta)\delta(\omega_1^{'}-\omega_3)\delta_{km}\delta(\omega_2^{'}-\omega_4)\delta_{ln}) \\
&= |\alpha_n|^2|\beta_m|^2\eta^2 \int d\omega_3 |S_2(\omega_3)|^2 \int d\omega_4 |S_1(\omega_4)|^2 + |\alpha_m|^2|\beta_n|^2(1-\eta)^2 \int d\omega_3 |S_1(\omega_3)|^2 \int d\omega_4 |S_2(\omega_4)|^2 \\
&\quad - \alpha_n^*\beta_m^*\alpha_m\beta_n\eta(1-\eta)\left|\int d\omega S_2^*(\omega)S_1(\omega)\right|^2 - \alpha_m^*\beta_n^*\alpha_n\beta_m\eta(1-\eta)\left|\int d\omega S_2^*(\omega)S_1(\omega)\right|^2 \\
&= |\alpha_n|^2|\beta_m|^2\eta^2 + |\alpha_m|^2|\beta_n|^2(1-\eta)^2 - (\alpha_n^*\beta_m^*\alpha_m\beta_n + \alpha_m^*\beta_n^*\alpha_n\beta_m)\eta(1-\eta)\Gamma,
\end{aligned} \tag{S85}$$

here $\delta(\omega)$ and δ_{ij} denote Dirac delta function and Kronecker delta respectively, and $\Gamma = \left|\int d\omega S_2^*(\omega)S_1(\omega)\right|^2$ represents indistinguishability of spectral modes of two photons, with the normalized conditions $\int d\omega\, |S_1(\omega)|^2 = \int d\omega\, |S_2(\omega)|^2 = 1$. The spectral indistinguishability Γ should be distinguished from the overlap c between the discrete modes. For the second equation in Eq. (S85), terms with odd number of operators in one port, such as $\langle 0|_3 \langle 0|_4 \hat{a}_3\hat{a}_3\hat{a}_3^{\dagger}\hat{a}_4^{\dagger} |0\rangle_3 |0\rangle_4$ and $\langle 0|_3 \langle 0|_4 \hat{a}_3\hat{a}_4\hat{a}_4^{\dagger}\hat{a}_4^{\dagger} |0\rangle_3 |0\rangle_4$, become zero and have been discarded. Summing Eq. (S85) over m and n, we can get the probability of the "fail" outcomes

$$\begin{aligned}
p(f) = \sum_{m,n} p(f,m,n) &= 1 - 2\eta + 2\eta^2 - 2\eta(1-\eta)\Gamma(\sum_{m=1}^{d} \alpha_m\beta_m^*)(\sum_{n=1}^{d} \alpha_n^*\beta_n) \\
&= 1 - 2\eta + 2\eta^2 - 2\eta(1-\eta)\Gamma c,
\end{aligned} \tag{S86}$$

where $c = |\langle\psi|\phi\rangle|^2 = \left|\sum_{m=1}^{d} \alpha_m\beta_m^*\right|^2$ is the overlap between discrete modes of two photons. When $\eta = 0.5$ and $\Gamma = 1$, Eq. (S86) becomes the usual form $p(f) = (1-c)/2$. In order to estimate the overlap c without bias, from Eq. (S86), the estimator of c should be corrected as

$$\tilde{c}_{ost} = \frac{1 - 2\eta + 2\eta^2}{2\eta(1-\eta)\Gamma} - \frac{k_f}{2\eta(1-\eta)N\Gamma}, \tag{S87}$$

where k_f is the number of "fail" outcomes out of N rounds of OST.

C. OST with pseudo photon-number-resolving detectors

In photonic experiments, deterministic photon-number-resolving detectors are not always available. In cases where we rely on threshold single photon detectors, such as avalanche photodiodes, the accurate measurement of multi-photon bunching term probabilities becomes challenging. This limitation affects the overlap estimation performance of the OST strategy when only pseudo photon-number-resolving detectors (PPNRD) are used. In the worst-case scenario, we consider an OST setup where the PPNRDs response "pass" outcomes, such as $|1_i 1_j\rangle_3 |0\rangle_4$ and $|2_i\rangle_3 |0\rangle_4$ in Eq. (S79), with only a probability of 1/2. The probability of detecting a "pass" outcome is the same as the probability of losing it, which is $(1+c)/4$. Therefore, the probability of detecting an outcome, which includes both "pass" and "fail" outcomes, can be expressed as

$$p(D) = p(f, D) + p(p, D) = \frac{1-c}{2} + \frac{1+c}{4} = \frac{3-c}{4}, \tag{S88}$$

where f and p denote "fail" and "pass" outcomes of the OST, respectively, and D represents the outcome captured by PPNRDs. The conditional PDF for "fail" and "pass" outcomes is given by

$$p(f|D) = \frac{p(f,D)}{p(D)} = \frac{2-2c}{3-c},\ p(p|D) = \frac{p(p,D)}{p(D)} = \frac{1+c}{3-c}, \tag{S89}$$

where the overlap $c \in [0,1]$. In this PPNRD scenario, the number of copies of quantum states used in the OST strategy is also non-deterministic due to the counting procedure based on detection. To accurately count the number of consumed copies of quantum states, a detected "pass" outcome will correspond to two rounds of the OST. In our analysis, we assume that we have detected k_p "pass" and k_f "fail" outcomes, and N is even. For N pairs of states $|\psi\rangle |\phi\rangle$, k_f and k_p must satisfy the restriction: $k_f + 2k_p = N$ if k_f is even, $k_f + 2k_p = N+1$ if k_f is odd and the last outcome is "pass". The conditional PDF of k_f is given by

$$p(k_f|D) = \begin{cases} \binom{\frac{N+k_f}{2}}{k_f} (\frac{2-2c}{3-c})^{k_f} (\frac{1+c}{3-c})^{\frac{N-k_f}{2}}, k_f \text{ is even} \\ \binom{\frac{N+k_f-1}{2}}{k_f} (\frac{2-2c}{3-c})^{k_f} (\frac{1+c}{3-c})^{\frac{N-k_f+1}{2}}, k_f \text{ is odd} \end{cases},\ k_f \in \{0, 1, ..., N\}. \tag{S90}$$

With the PDF of k_f, we can get the normalizing condition, the first and the second order raw moments of k_f as follows:

$$\begin{aligned} \langle 1 \rangle_{k_f} &= \frac{1 + (\frac{1+c}{3-c})^{N+1}}{1 + (\frac{1+c}{3-c})} - \frac{(\frac{1+c}{3-c})^{N+1} - (\frac{1+c}{3-c})}{1 + (\frac{1+c}{3-c})} = 1, \\ \langle k_f \rangle_{k_f} &= \frac{1}{8} [(1-c)(4N+1+c) - (\frac{1+c}{3-c})^N (1-c^2)] = \frac{1}{8}(1-c)(4N+1+c) + O(\frac{1}{N}), \\ \langle k_f^2 \rangle_{k_f} &= \frac{N}{4}(1-c)[2 + c(1-c) + N(1-c)] + O(1), \end{aligned} \tag{S91}$$

where the expression $(\frac{1+c}{3-c})^N (1-c^2)$ scales as $O(1/N)$. The mean of estimated overlap in the OST strategy is given by

$$\left\langle 1 - \frac{2k_f}{N} \right\rangle_{k_f} = 1 - \frac{2}{N} \langle k_f \rangle_{k_f} = c + O(\frac{1}{N}), \tag{S92}$$

here we show that the overlap estimator in OST strategy is asymptotically unbiased. Then the overlap estimation variance for OST strategy in this PPNRD scenario is given by

$$\begin{aligned} v_{ost}(c, N) = \left\langle (1 - \frac{2k_f}{N} - c)^2 \right\rangle_{k_f} &= (1-c)^2 - \frac{4}{N}(1-c) \langle k_f \rangle_{k_f} + \frac{4}{N^2} \langle k_f^2 \rangle_{k_f} \\ &= \frac{(3-c)(1-c^2)}{2N} + O(\frac{1}{N^2}) \approx \frac{(3-c)(1-c^2)}{2N}. \end{aligned} \tag{S93}$$

Compared with the variance $v(c, N) = (1-c^2)/N$ through the ideal OST, the additional factor $(3-c)/2$ in Eq. (S93) reflects the precision reduction introduced by the PPNRDs.

D. OST strategy precision with our experimental setup

The preceding discussion highlights the feasibility of using the OST strategy to estimate the overlap c without bias, even in the presence of imperfect HOMI equipment and partially indistinguishable photons. In this section, we present the theoretical results on the precision of the OST strategy using the experimental setup mensioned in the maint text, as depicted in Fig. S7. The detector is employed to detect "pass" outcomes from the output port 3 (4) of NPBS-1 with a detection probability of 1/2, similar to the PPNRD scenario discussed earlier. We note that the corrected estimator in Eq. (S87) is more sensitive to variations in Γ compared to η, especially when $\Gamma = 1$ and $\eta = 0.5$. For our experimental setup, the reflectivity of NPBS-1 is approximately 0.53, and the imperfections of NPBS can be neglected. Therefore, we utilize $\tilde{c}_{ost} = (1 - 2k_f/N)/\Gamma$ to estimate the overlap. In this case, we modify the overlap in Eq. (S93) as $c' = c \cdot \Gamma$. Here, c represents the overlap between two quantum states that are encoded on polarization of two photons, and is the parameter we aim to estimate. Using the basic property of variance, we derive the overlap estimation variance for the OST strategy under our experimental setup as

$$v_{ost}(c, N) = \frac{(3 - \Gamma c)(1 - \Gamma^2 c^2)}{2N\Gamma^2}. \tag{S94}$$

The value of the spectral mode indistinguishability Γ can be calibrated by performing the OST between two photons with identical encoded states on polarization. In our setup, Γ is measured to be 0.965 ± 0.008, obtained as the average maximum HOMI visibility when different polarized photon pairs are used as inputs. We can express the variance of the OST strategy more insightfully as follows:

$$v_{ost}(c, N) = \frac{(3 - \Gamma c)}{2} \times \left(\frac{1 - \Gamma^2}{N\Gamma^2} + \frac{1 - c^2}{N} \right). \tag{S95}$$

This expression reveals three distinct contributions to the variance. The overlap-dependent factor $(3 - \Gamma c)/2$, arising from the PPNRDs, reaches its maximum at $c = 0$ and minimum at $c = 1$. This behavior indicates that the detrimental effects of PPNRDs are most pronounced when the overlap is small. The overlap-independent term $(1 - \Gamma^2)/N\Gamma^2$ captures the impact of partial photon indistinguishability, leading to a constant reduction in precision across the entire range of overlaps. The final term $(1 - c^2)/N$ matches the variance of both the ideal swap test and the SCM strategy. This observation confirms that, the OST strategy and the SCM strategy would exhibit identical performance without experimental imperfections.

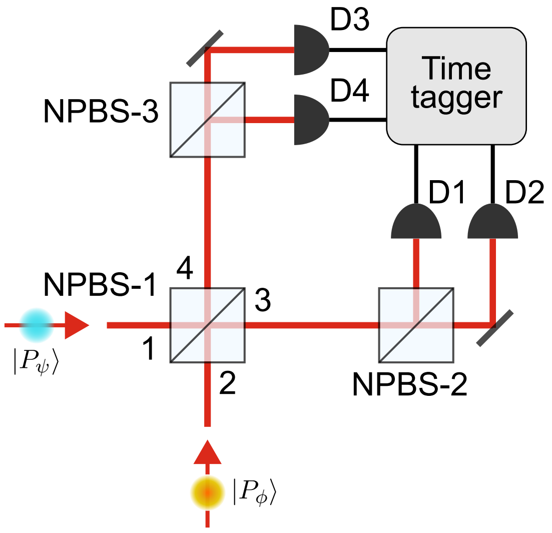

FIG. S7. Experimental setup for Hong-Ou-Mandel interference and pseudo photon-number-resolving detection. Two photons $|P_\psi\rangle$ and $|P_\phi\rangle$, with their polarization modes encoded as $|\psi\rangle$ and $|\phi\rangle$ respectively, undergo interference at NPBS-1. The pseudo photon-number-resolving detector (PPNRD) is achieved using NPBS-2 (NPBS-3) and two single photon counting modules (SPCMs). The electrical signals form SPCMs (D1 to D4) are processed by time tagger (Time Tagger Ultra, Swabian) to produce coincidence counts. NPBS: non-polarizing beam splitter.

Fisher information in OST strategy. Considering the spectral mode indistinguishability $\Gamma < 1$, we rewrite the

probabilities in Eq. (S89) as follows:

$$p(F) = p(f|D) = \frac{2-2\Gamma c}{3-\Gamma c},\ p(P) = p(p|D) = \frac{1+\Gamma c}{3-\Gamma c}, \tag{S96}$$

where $p(F)$ and $p(P)$ represent the original detection probabilities for "fail" and "pass" outcomes. From the Bernoulli distribution, the Fisher information of overlap in a detected OST event is given by

$$\begin{aligned} I_{ost} =& \frac{1}{p(F)}(\frac{\partial p(F)}{\partial c})^2 + \frac{1}{p(P)}(\frac{\partial p(P)}{\partial c})^2 = \frac{8\Gamma^2}{(3-\Gamma c)^3(1-\Gamma c)} + \frac{16\Gamma^2}{(3-\Gamma c)^3(1+\Gamma c)} \\ =& \frac{8\Gamma^2}{(3-\Gamma c)^2(1-\Gamma^2 c^2)}. \end{aligned} \tag{S97}$$

Compared with other overlap estimation strategies, the OST strategy should take into account the photon loss in PPNRD. For N copies of state pairs, the total Fisher information of overlap is given by $N' I_{ost} = 2N\Gamma^2/(3-\Gamma c)(1-\Gamma^2 c^2)$, where $N' = (3-\Gamma c)N/4$ is the mean number of detected events. We define the effective Fisher information of overlap per state pair as

$$I^e_{ost} = \frac{N' I^{ost}}{N} = \frac{2\Gamma^2}{(3-\Gamma c)(1-\Gamma^2 c^2)} \approx \frac{1}{N v_{ost}(c,N)}, \tag{S98}$$

where $v_{ost}(c, N)$ is defined in Eq. (S94). Therefore, we show that the estimator used in OST under our PPNRD scenario saturates the corresponding Cramér-Rao bound asymptotically.

IV. EXPERIMENTAL DETAILS

Photon source. Light pulses with 150 fs duration, centered at 830 nm, from a ultrafast Ti-Sapphire Laser (Coherent Mira-HP; 76 MHz repetition rate) are firstly frequency doubled in a β-type barium borate (β-BBO) crystal to generate a second harmonic beam with 415 nm wavelength. Then the upconversion beam is then utilized to pump another β-BBO with phase-matched cut angle for type-II beam-like degenerate spontaneous down conversion (SPDC) which produces pairs of photons, denoted as signal and idler. The signal and idler photons possess distinct emergence angles and spatially separate from each other. After passing through two clean-up filters with a 3 nm bandwidth, the photons are coupled into separate single-mode fibers. The idler mode is detected by a single photon counting module (SPCM, Excelitas Technologies) with a detection efficiency of approximately 55%, serving as a trigger. This configuration enables the photon source module to function as a herald single-photon source (HSPS). The signal mode is directed to the Tomography, Projection, and SCM modules, as mentioned in the main text, for further experimental operations. Additionally, both the signal and idler photons are directed to the OST module, where they undergo Hong-Ou-Mandel interference.

State preparation. In TP, TT and OST strategy experiments, qubits are encoded in the polarization degree of freedom of photons, i.e., $\{|0\rangle = |H\rangle, |1\rangle = |V\rangle\}$, where $|H\rangle$ and $|V\rangle$ represent horizontal and vertical polarization, respectively. With a electronically controlled half wave-plate (E-HWP, Newport PR50PP Motorized Rotation Stage) followed by a liquid crystal phase retarder (LCPR, Thorlabs, LCC1113-B), the single photon state is prepared as

$$|\psi\rangle \text{ or } |\phi\rangle = \cos 2\theta\, |H\rangle + e^{i\alpha} \sin 2\theta\, |V\rangle\,, \tag{S99}$$

where θ is the E-HWP angle and α is the relative phase between two polarization modes added by LCPR. In experiments, we configure HWPs and LCPRs sequentially for state preparation, ensuring each setting is complete before recording the measurement results. The precision of state preparation has been characterized by quantum state tomography, with an average fidelity up to 0.9989, as shown in Fig. S8. In the SCM strategy experiment, the single photon will be prepared as a two-qubit joint state $|\psi\rangle |\phi\rangle$. After encoding the first qubit $|\psi\rangle$ in the same form as Eq. (S99), a birefringent calcite beam splitter (BD) splits the single photon into two path modes, resulting in a path-polarised entangled state

$$\cos 2\theta_1 |s_0\rangle |H\rangle + e^{i\alpha_1} \sin 2\theta_1 |s_1\rangle |V\rangle\,, \tag{S100}$$

where s_0 and s_1 denote the lower and upper path modes, respectively. In the path mode s_1, a HWP with angle 45° rotates $|V\rangle$ to $|H\rangle$. Another HWP with angle 0° is placed in path mode s_0 for optical path compensation. Then the state becomes

$$\cos 2\theta_1 |s_0\rangle |H\rangle + e^{i\alpha_1} \sin 2\theta_1 |s_1\rangle |H\rangle\,. \tag{S101}$$

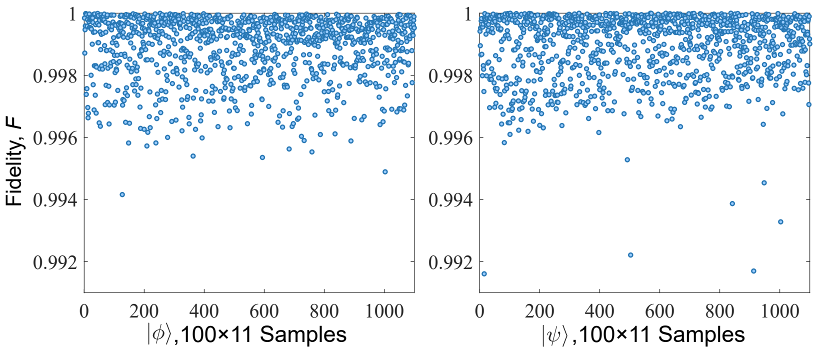

FIG. S8. Fidelity of state preparation in TT strategy. The fidelity is defined as $F = |\langle\phi|\tilde{\phi}\rangle|^2$, where $|\phi\rangle$ is the target state and $|\tilde{\phi}\rangle$ is the state reconstructed by quantum state tomography using 180,000 measurements.

The second qubit $|\phi\rangle$ is encoded on the single photon using an E-HWP and a phase retarder implemented with an E-HWP sandwiched by two quarter-wave plates (QWP-HWP-QWP configuration, QHQ) to circumvent the non-uniform adding phase on different position of the LCPR. The QHQ phase retarder implements a unitary transformation in each path mode using two QWPs set at 45° in combination with an E-HWP at an angle of $(\alpha_2 - \pi)/4$, given by

$$U_{QHQ} = |H\rangle\langle H| + e^{i\alpha_2}|V\rangle\langle V|, \tag{S102}$$

where a global phase is ignored. After going through the second E-HWP with angle θ_2 and the QHQ phase retarder, the two-qubit state of single photons can be prepared as

$$\begin{aligned}|\psi\rangle\otimes|\phi\rangle =& \cos 2\theta_1\cos 2\theta_2|s_0\rangle|H\rangle + e^{i\alpha_2}\cos 2\theta_1\sin 2\theta_2|s_0\rangle|V\rangle\\ &+ e^{i\alpha_1}\sin 2\theta_1\cos 2\theta_2|s_1\rangle|H\rangle + e^{i(\alpha_1+\alpha_2)}\sin 2\theta_1\sin 2\theta_2|s_1\rangle|V\rangle\\ =&(\cos 2\theta_1|s_0\rangle + e^{i\alpha_1}\sin 2\theta_1|s_1\rangle)\otimes(\cos 2\theta_2|H\rangle + e^{i\alpha_2}\sin 2\theta_2|V\rangle).\end{aligned} \tag{S103}$$

Measurements. For measurement modules in each strategy, photon detection is performed by multiple SPCMs. The detected counts are processed by a time tagger (Swabian, Ultra Performance), which outputs the measurement results. In the tomography module, a HWP and a QWP with three angle configurations {HWP, QWP}: $\{22.5°, 0°\}$, $\{0°, -45°\}$, $\{0°, 0°\}$, followed by a BD and two SPCMs, perform measurements of the three Pauli operators $(\hat{\sigma}_x, \hat{\sigma}_y, \hat{\sigma}_z)$, respectively. In the projection module, the LCPR and the E-HWP perform the inverse unitary $U^\dagger$, where $U^\dagger|\tilde{\phi}\rangle = |0\rangle$, to realize the projection onto $|\tilde{\phi}\rangle$. The successful projection is indicated by the detection of the photon in the horizontal polarization.

In the SCM module, the Schur collective measurement based on the Schur transform consists of four projectors:

$$\hat{E}_1 = |00\rangle\langle 00|,\ \hat{E}_2 = |11\rangle\langle 11|,\ \hat{E}_+ = |\Psi_+\rangle\langle\Psi_+|,\ \hat{E}_- = |\Psi_-\rangle\langle\Psi_-|, \tag{S104}$$

where $|00\rangle,\ |11\rangle,\ |\Psi_+\rangle = \frac{1}{\sqrt{2}}(|01\rangle + |10\rangle)$ are the triplet states and $|\Psi_-\rangle = \frac{1}{\sqrt{2}}(|01\rangle - |10\rangle)$ is the singlet state. $\hat{E}_-$ is the projector onto the anti-symmetric subspace. The combination of the first three projectors, $\hat{E}_1 + \hat{E}_2 + \hat{E}_+$, forms a single measurement $\hat{E}_{sym}$, which projects onto the symmetric subspace. The probabilities of projecting a two-qubit state $|\psi\rangle|\phi\rangle$ onto the symmetric and anti-symmetric subspaces solely depend on the overlap c.

The measurement setup of SCM is illustrated in Fig. S9. The initial state is prepared as a general two-qubit state

$$|\Phi_0\rangle = a_1|s_0, H\rangle + a_2|s_0, V\rangle + a_3|s_1, H\rangle + a_4|s_1, V\rangle,\ |a_1|^2 + |a_2|^2 + |a_3|^2 + |a_4|^2 = 1, \tag{S105}$$

where two-qubit state is encoded as $|00\rangle = |s_0, H\rangle, |01\rangle = |s_0, V\rangle, |10\rangle = |s_1, H\rangle, |11\rangle = |s_1, V\rangle$. The first BD evolves the initial state as

$$|\Phi_1\rangle = a_1|s_{-1}, H\rangle + a_2|s_0, V\rangle + a_3|s_0, H\rangle + a_4|s_1, V\rangle, \tag{S106}$$

where s_{-1} denotes the additional path mode introduced by the first BD. The state after HWP with angle 22.5° on the path mode s_0 is

$$|\Phi_2\rangle = a_1|s_{-1}, H\rangle + \frac{(a_3 - a_2)}{\sqrt{2}}|s_0, V\rangle + \frac{(a_3 + a_2)}{\sqrt{2}}|s_0, H\rangle + a_4|s_1, V\rangle. \tag{S107}$$

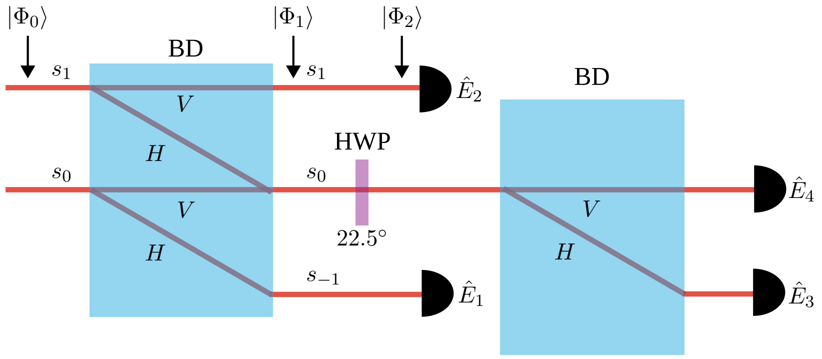

FIG. S9. Realization of the Schur collective measurement. The input state $|\Phi_0\rangle$ is a two-qubit state, encoded using the polarization and path degrees of a single photon. The final detection of photons is achieved using four single photon counting modules (SPCMs). BD beam displacer, HWP half wave-plate.

The two SPCMs following the first BD realize the first two projectors in Eq. (S104) with the detection probabilities as

$$\begin{aligned} p_1 &= |a_1|^2 = |\langle s_{-1}, H|\Phi_2\rangle|^2 = \langle\Phi_0|\hat{E}_1|\Phi_0\rangle, \\ p_2 &= |a_4|^2 = |\langle s_1, V|\Phi_2\rangle|^2 = \langle\Phi_0|\hat{E}_2|\Phi_0\rangle. \end{aligned} \tag{S108}$$

The second BD together with two SPCMs realize the last two projectors with the detection probabilities as

$$\begin{aligned} p_+ &= \left|\frac{(a_3+a_2)}{\sqrt{2}}\right|^2 = |\langle s_0, H|\Phi_2\rangle|^2 = \langle\Phi_0|\hat{E}_+|\Phi_0\rangle, \\ p_- &= \left|\frac{(a_3-a_2)}{\sqrt{2}}\right|^2 = |\langle s_0, V|\Phi_2\rangle|^2 = \langle\Phi_0|\hat{E}_-|\Phi_0\rangle. \end{aligned} \tag{S109}$$

When the initial state takes as a two-qubit product state as

$$|\Phi_0\rangle = |\psi\rangle \otimes |\phi\rangle = (b_1|0\rangle + b_2|1\rangle) \otimes (d_1|0\rangle + d_2|1\rangle) = b_1d_1|00\rangle + b_1d_2|01\rangle + b_2d_1|10\rangle + b_2d_2|11\rangle, \tag{S110}$$

the outcome probability of $\hat{E}_-$ is given by

$$p_- = \left|\frac{b_2d_1 - b_1d_2}{\sqrt{2}}\right|^2 = \frac{1}{2}(|b_2|^2|d_1|^2 + |b_1|^2|d_2|^2 - b_1^*b_2d_1d_2^* - b_1b_2^*d_1^*d_2) = \frac{1-|\langle\psi|\phi\rangle|^2}{2}, \tag{S111}$$

here the overlap c is given by $|\langle\psi|\phi\rangle|^2 = |b_1|^2|d_1|^2 + |b_2|^2|d_2|^2 + b_1^*b_2d_1d_2^* + b_1b_2^*d_1^*d_2$ with normalization conditions $|b_1|^2 + |b_2|^2 = 1$ and $|d_1|^2 + |d_2|^2 = 1$. The outcome probabilities of the other three projectors can be combined to a single one associated with the overlap as

$$p_1 + p_2 + p_+ = 1 - p_- = \frac{1+|\langle\psi|\phi\rangle|^2}{2}. \tag{S112}$$

We note that the SCM yields the same probability distribution as the ideal OST in Eq. (S80). In fact, the SCM strategy is equivalent to the Bell-basis algorithm proposed in [15], as an improved version of the swap test.

V. SUPPLEMENTARY RESULTS

Overlap estimation with a known state. In this section, we discuss the overlap estimation when one of the two states, denoted as $|\phi\rangle$, is already known. The optimal strategy in this scenario involves projecting the unknown state $|\psi\rangle$ onto $|\phi\rangle$ using N copies of $|\psi\rangle$. The number of successful projections k follows a binomial distribution $\mathrm{Bin}(k, N, c)$, and we estimate the overlap by the successful fraction k/N. The average variance through projection-based overlap estimation with a known state is given by $v_{proj} = c(1-c)/N$.

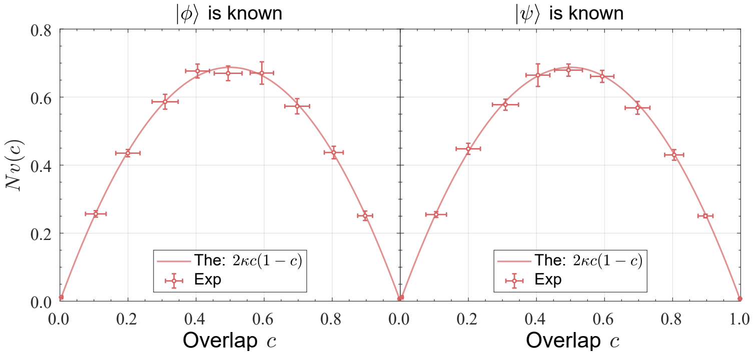

FIG. S10. Experimental and theoretical results of scaled average variance $Nv(c)$ for tomography-based overlap estimation with a known state. The experimental results (markers) show good agreement with the theoretical results (solid lines).

Additionally, we can reconstruct the unknown state $|\psi\rangle$ as $|\tilde{\psi}\rangle$ through quantum state tomography and calculate the estimated overlap $\tilde{c}_{tomo} = |\langle\tilde{\psi}|\phi\rangle|^2$. We derive $\tilde{c}_{tomo}$ from Eq. (S38) by only considering the errors form $|\tilde{\psi}\rangle$ and approximating it as:

$$\begin{aligned}\tilde{c}_{tomo} &= \frac{1}{2}\left[1 + (2c-1)\cos\chi_1 + 2\sqrt{c(1-c)}\cos(\zeta_1 - \varphi)\sin\chi_1\right] \\ &\approx c + \sqrt{c(1-c)}(t_1^c\cos\varphi + t_1^s\sin\varphi) - \frac{2c-1}{4}\chi_1^2,\end{aligned} \tag{S113}$$

where higher-order terms in the second equation are neglected. With the analysis in Section II B, the average variance of tomography-based overlap estimation with a known state can be expressed as:

$$\begin{aligned}v_{tomo} = \overline{\langle(\tilde{c}_{tomo} - c)^2\rangle} &= \frac{c(1-c)}{2}\left(\overline{\langle(t_1^c)^2\rangle} + \overline{\langle(t_1^c)^2\rangle}\right) + O(\frac{1}{N^2}) \\ &= \frac{2\kappa c(1-c)}{N} + O(\frac{1}{N^2}).\end{aligned} \tag{S114}$$

To verify this theoretical result, we utilize the experimental data from the TT strategy to get experimental results for tomography-based overlap estimation with a known state. For the known state, we employ the complete measurement results (180,000 copies) to reconstruct the exact state. For the unknown state, we reconstruct the estimated state using $N = 900$ copies. This allows us to obtain two groups of average variance results, as depicted in Fig. S10. Consequently, we can attribute the errors in TT and TP strategy to the combinations of v_{tomo} and v_{proj}, as mentioned in the main text.

Overlap estimation overhead. Our analysis of various overlap estimation strategies shows that the average variance for all of them can be represented in a similar form: $v_s(c, N) = f_s(c)/N$, where $f_s(c)$ denotes the scaled average variance and N is the number of copies. We can determine the overhead for overlap estimation from these variance results using Chebyshev's inequality. Specifically, we can estimate the overlap $\tilde{c}_s$ with the probability

$$\Pr(|\tilde{c}_s - c| \geq \varepsilon) \leq \frac{v_s(c,N)}{\varepsilon^2} = \frac{f_s(c)}{N\epsilon^2} = \eta, \tag{S115}$$

where ε is the estimation error bound and η is a threshold probability. Given a pair of ε and η, the estimation error is less than ε with a probability greater than $1-\eta$ by consuming $N \sim f_s(c)/\eta\varepsilon^2$ pairs of states, which represents the overhead for strategy s. From $\varepsilon = \sqrt{f_s(c)/N\eta}$, the estimation error ε for all strategies scales as $O(1/\sqrt{N})$.

Extending the SCM strategy to qudits. The SCM strategy can be naturally extended to higher dimensions, where $|\psi\rangle$ and $|\phi\rangle$ are defined in a d-dimensional Hilbert space:

$$|\psi\rangle = \sum_{i=0}^{d-1}\alpha_i|i\rangle, \quad |\phi\rangle = \sum_{j=0}^{d-1}\beta_j|j\rangle, \tag{S116}$$

where $|i\rangle$ represents the basis for the qudits. The SCM strategy for qudits utilizes a POVM with two elements:

$$\hat{E}_{sym} = \hat{I} - \hat{E}_{ans}, \;\; \hat{E}_{ans} = \sum_{i<j}|\Pi_{ij}\rangle\langle\Pi_{ij}|, \tag{S117}$$

where $\hat{E}_{ans}$ projects onto the anti-symmetric subspace, spanned by the vectors:

$$|\Pi_{ij}\rangle = \frac{1}{\sqrt{2}}\left(|i\rangle|j\rangle - |j\rangle|i\rangle\right), \quad i < j. \tag{S118}$$

The rank of $\hat{E}_{ans}$ is $d(d-1)/2$. The probability of obtaining the outcome associated with $\hat{E}_{ans}$, denoted as p_{ans}, is given by:

$$\begin{aligned} p_{ans} &= \langle\psi|\langle\phi|\hat{E}_{ans}|\psi\rangle|\phi\rangle \\ &= \frac{1}{2}\sum_{i<j}|\alpha_i\beta_j - \alpha_j\beta_i|^2 = \frac{1}{4}\sum_{i\neq j}|\alpha_i\beta_j - \alpha_j\beta_i|^2 = \frac{1}{4}\sum_{i,j=0}^{d-1}|\alpha_i\beta_j - \alpha_j\beta_i|^2 \\ &= \frac{1}{2}\sum_{i=0,j=0}^{d-1}|\alpha_i|^2|\beta_j|^2 - \frac{1}{2}(\sum_{i=0}^{d-1}\alpha_i\beta_i^*)(\sum_{j=0}^{d-1}\alpha_j^*\beta_j) \\ &= \frac{1}{2}(1 - |\langle\psi|\phi\rangle|^2). \end{aligned} \tag{S119}$$

Therefore, the overlap is given by $c = 1 - 2p_{ans}$. The estimation variance for high-dimensional SCM strategy is still $v_{scm}(c, N) = (1 - c^2)/N$ and independent of d. This result highlights the scalability of the SCM strategy for overlap estimation in high-dimensional quantum systems.

Hong-Ou-Mandel interference. As shown in Fig. S7, our experiments for the OST strategy involve two-mode Hong-Ou-Mandel interference (HOMI) between two input photons, with the delay between their arrival times at the interference NPBS set to zero. We use four SPCMs which are threshold photon detectors, to record the detection events of the 6 two-fold coincidence channels. We observe the HOMI varying with delay (path difference) for all 6 outcomes when two input photons are both in horizontal polarization mode, and the results are illustrated in Fig. S11. The two coincidence channels between the SPCMs located on same sides of NPBS-1, as shown in Fig. S11a, correspond to the"pass" outcomes in OSTs, which manifest as peaks in HOMI pattern. The other four coincidence channels, corresponding to the "fail" outcomes, manifest as dips in Fig. S11b. The high probability of "pass" outcomes when the delay is zero is consistent with the overlap estimation results obtained through the OST strategy when the overlap is 1.

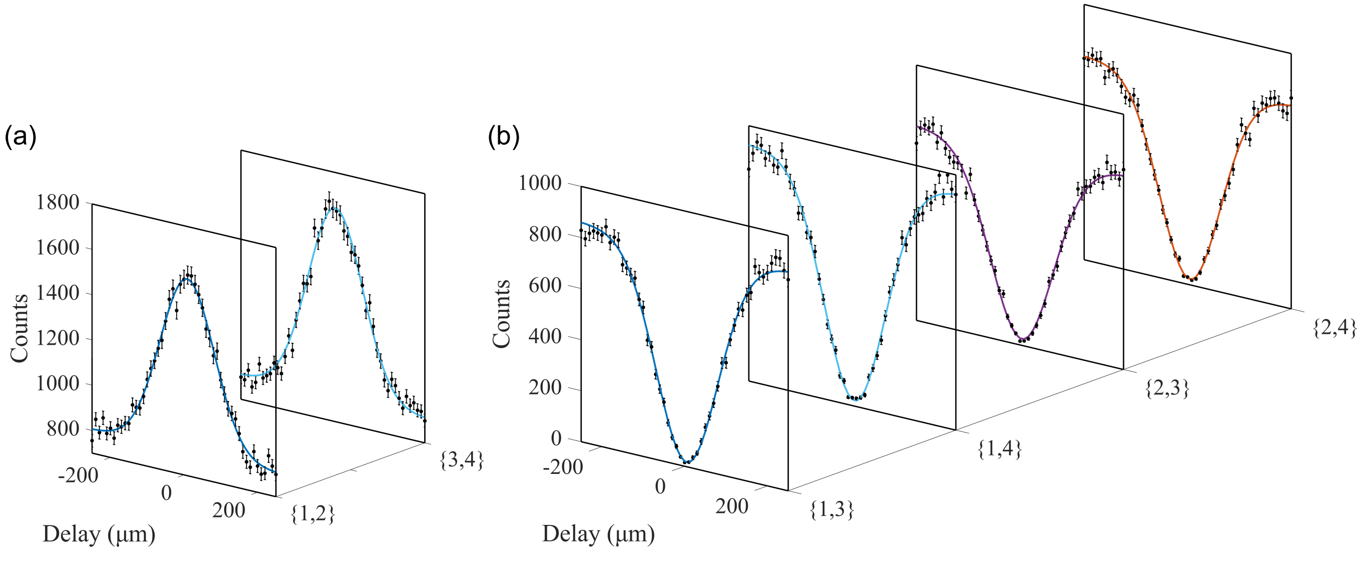

FIG. S11. Hong-Ou-Mandel interference patterns for the 6 coincidence channels. **a** 2 one-side (of the NPBS-1 in Fig. S7) coincidence channels correspond to the "pass" outcomes in optical swap tests. **b** 4 two-side coincidence channels correspond to the "fail" outcomes. The colored solid lines represent curve fittings of the data (black dot) to a Gaussian function, while the error bars indicate uncertainties assuming Poisson count statistics.

Uncertainties of experimental measured $Nv(c)$. As mentioned in the main text, the scaled average variance $Nv(c)$ measured in our experiments exhibits two types of uncertainties, represented by vertical and horizontal error

bars in main text Fig. 2. Vertical errors indicate statistical uncertainties arising from the finite number of measuring the average variance, which can be reduced by increasing the number n of experimental runs.

Horizontal errors reflect systematic uncertainties associated with our experimental setups for both state preparation and measurements. In state preparation, for each target overlap c, 100 qubit pairs are required to be prepared with the same overlap. However, due to experimental imperfections, the actual overlaps of different qubit pairs may deviate from the target value, leading to horizontal uncertainties in $Nv(c)$. Furthermore, systematic errors introduced by the measurement setups can result in biased estimations of the true overlaps, further contributing to the horizontal uncertainties.

These systematic errors can be attributed to imperfections in the optical elements used in our experimental setups. Specifically, the HWP and QWP suffer misalignment of the optics axis (typically ~ 0.1 degree), retardation errors (typically $\sim \lambda/300$ where $\lambda = 830$nm) and inaccuracies in setting angles (typically ~ 0.2 degree). The LCPR is pre-calibrated by a co-linear inteferometer formed by an HWP-PR-HWP configuration and a BD, introducing some experimental errors. In the SCM and OST strategies, the interference visibility may experience slow drift and slight vibrations during the measurement process. In the SCM strategy, the average interference visibility between the two BDs is above 99.8%, resulting in a minor influence on the precision of overlap estimation. Conversely, in the OST strategy, the average HOM interference visibility is approximately 96.5%, significantly affecting the overlap estimation precision, as discussed in Section III B. It is worth noting that the TT and TP strategies are subject to more systematic errors compared to SCM and OST. This is evident from the larger horizontal uncertainties observed in TT and TP, in contrast to OST and SCM. This phenomenon can be attributed to the fact that TT and TP involve more measurement configurations, such as the three Pauli operators in the tomography process and projecting onto different states in the projection process. In contrast, the measurement setups in SCM and OST remain static, introducing fewer systematic errors and resulting in smaller horizontal uncertainties.

---

[1] M. Fanizza, M. Rosati, M. Skotiniotis, J. Calsamiglia, and V. Giovannetti, Beyond the Swap Test: Optimal Estimation of Quantum State Overlap, Phys. Rev. Lett. **124**, 060503 (2020).
[2] H. de Guise, O. Di Matteo, and L. L. Sánchez-Soto, Simple factorization of unitary transformations, Phys. Rev. A **97**, 022328 (2018).
[3] A. Papoulis and S. Unnikrishna Pillai, *Probability, Random Variables and Stochastic Processes* (McGraw-Hill, 2002).
[4] E. Bagan, M. Baig, and R. Muñoz Tapia, Optimal scheme for estimating a pure qubit state via local measurements, Phys. Rev. Lett. **89**, 277904 (2002).
[5] S. Massar and S. Popescu, Optimal extraction of information from finite quantum ensembles, Phys. Rev. Lett. **74**, 1259 (1995).
[6] A. A. Mele, Introduction to Haar Measure Tools in Quantum Information: A Beginner's Tutorial, Quantum **8**, 1340 (2024).
[7] A. Hayashi, T. Hashimoto, and M. Horibe, Reexamination of optimal quantum state estimation of pure states, Phys. Rev. A **72**, 032325 (2005).
[8] D. Bruß and C. Macchiavello, Optimal state estimation for d-dimensional quantum systems, Physics Letters A **253**, 249 (1999).
[9] R. Kueng, H. Rauhut, and U. Terstiege, Low rank matrix recovery from rank one measurements, Applied and Computational Harmonic Analysis **42**, 88 (2017).
[10] J. Haah, A. W. Harrow, Z. Ji, X. Wu, and N. Yu, Sample-optimal tomography of quantum states, in *Proceedings of the Forty-Eighth Annual ACM Symposium on Theory of Computing*, STOC '16 (Association for Computing Machinery, New York, NY, USA, 2016) p. 913–925.
[11] C. Davis and W. M. Kahan, The Rotation of Eigenvectors by a Perturbation. III, SIAM Journal on Numerical Analysis **7**, 1 (1970).
[12] R. O'Donnell and J. Wright, Efficient quantum tomography ii, in *Proceedings of the 49th Annual ACM SIGACT Symposium on Theory of Computing*, STOC 2017 (Association for Computing Machinery, New York, NY, USA, 2017) p. 962–974.
[13] S. T. Flammia, D. Gross, Y.-K. Liu, and J. Eisert, Quantum tomography via compressed sensing: error bounds, sample complexity and efficient estimators, New Journal of Physics **14**, 095022 (2012).
[14] N. Yu, Sample efficient tomography via Pauli Measurements, arXiv:2009.04610 (2020).
[15] L. Cincio, Y. Subaşı, A. T. Sornborger, and P. J. Coles, Learning the quantum algorithm for state overlap, New Journal of Physics **20**, 113022 (2018).
