# Supplementary material for: Experimental benchmarking of quantum state overlap estimation strategies with photonic systems
Source: Light Sci Appl. 2025 Feb 12;14:83. doi: 10.1038/s41377-025-01755-8 (PMC11814415; doi:10.1038/s41377-025-01755-8)
Supplement: Supplementary file 2 — Source files of figures in the main text [file 41377_2025_1755_MOESM2_ESM.zip › Figures_Main_Text/links/Fig1_Schematic_Setup_LaTeX2AI_2cefff0e6177e0a.pdf]

U_{Sch}
