# Supplementary figures and images for: Experimental benchmarking of quantum state overlap estimation strategies with photonic systems
Source: Light Sci Appl. 2025 Feb 12;14:83. doi: 10.1038/s41377-025-01755-8 (PMC11814415; doi:10.1038/s41377-025-01755-8)

## a Schematics

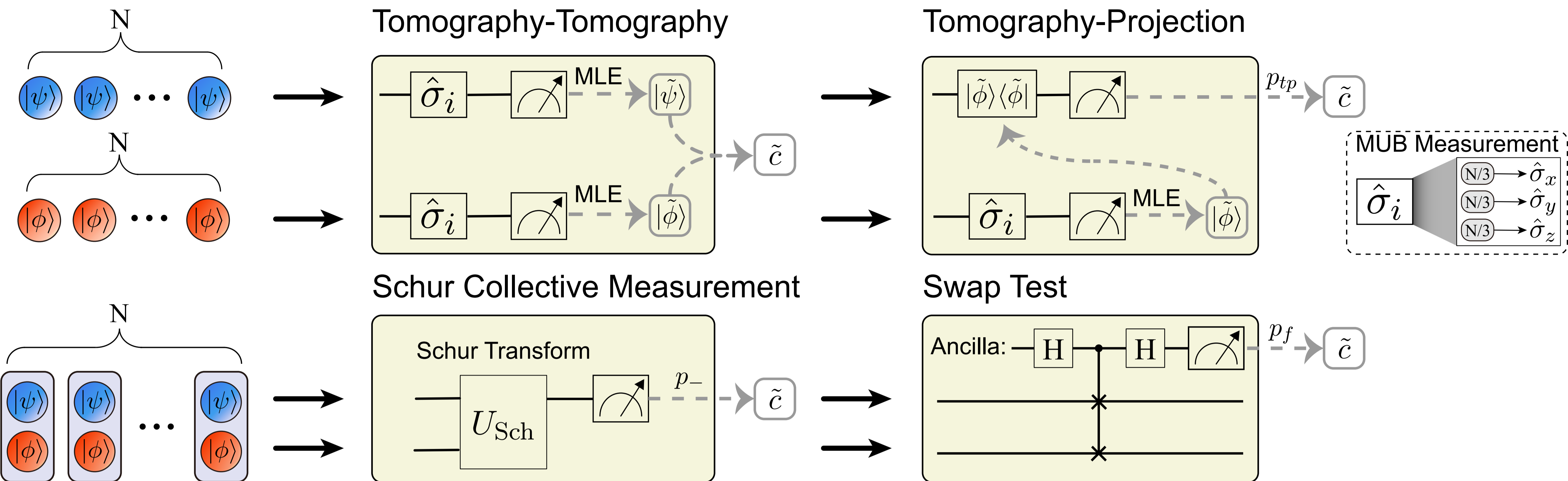

## b Experimental Setups

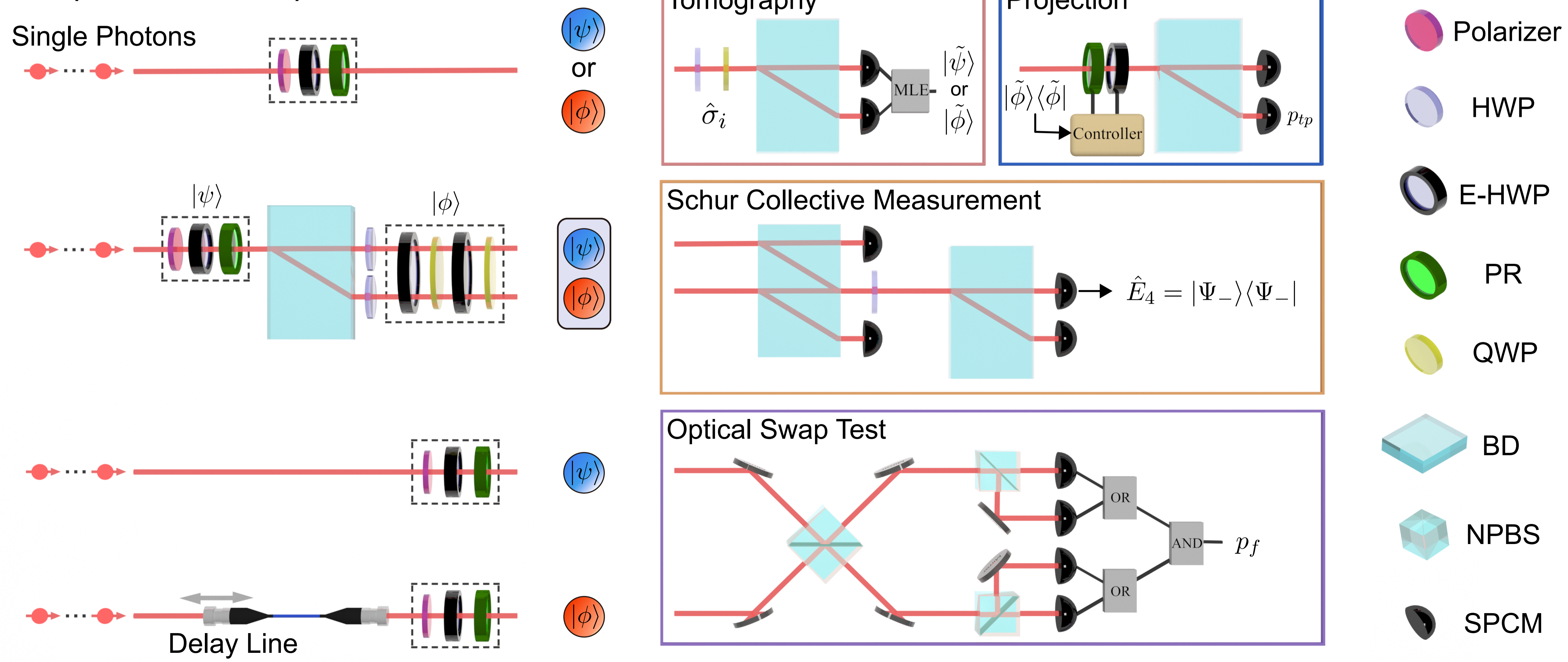

Supplement: Supplementary file 2 — Source files of figures in the main text [file 41377_2025_1755_MOESM2_ESM.zip › Figures_Main_Text/Fig1_Schematic_Setup.pdf]

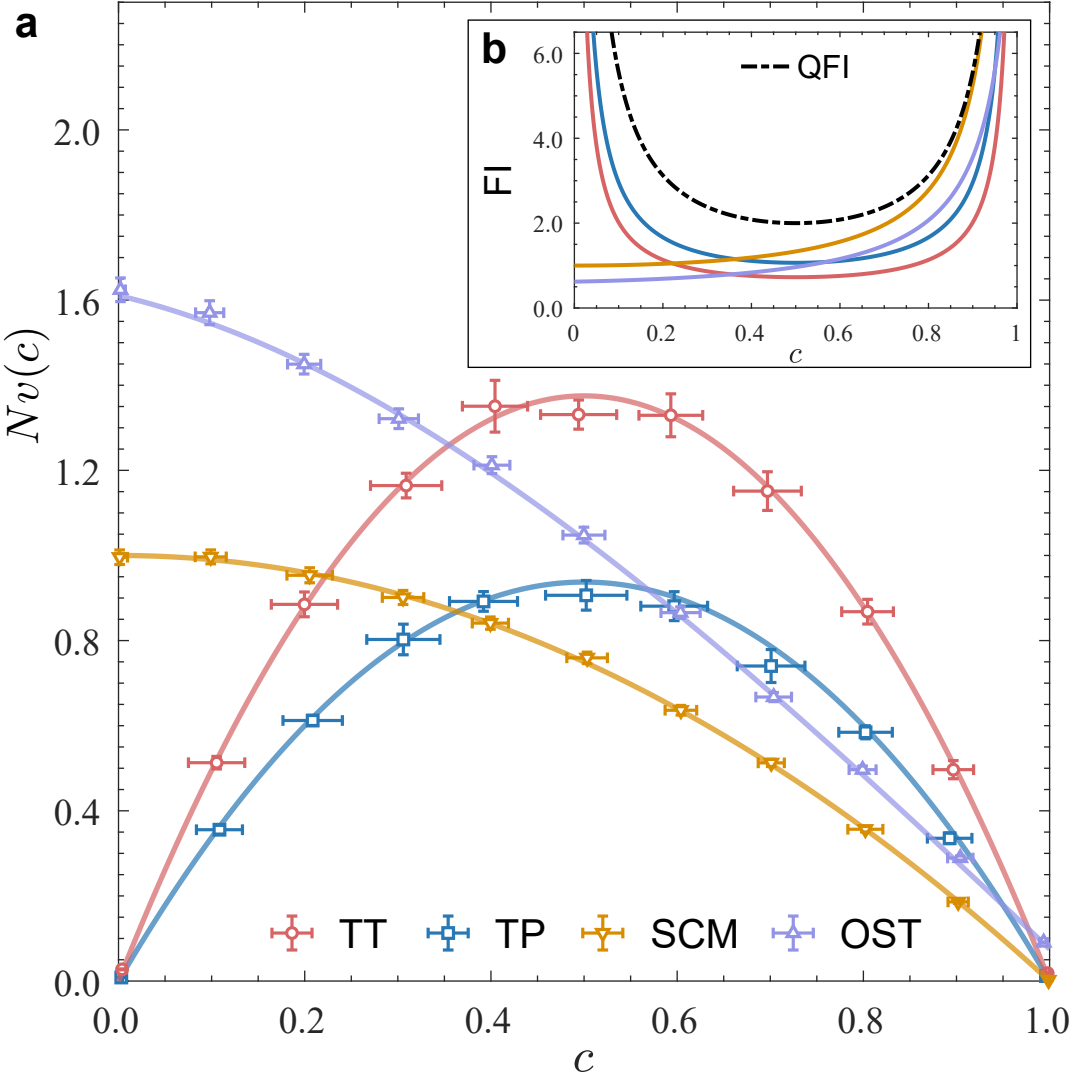

Supplement: Supplementary file 2 — Source files of figures in the main text [file 41377_2025_1755_MOESM2_ESM.zip › Figures_Main_Text/Fig2_Data_OVE.pdf]

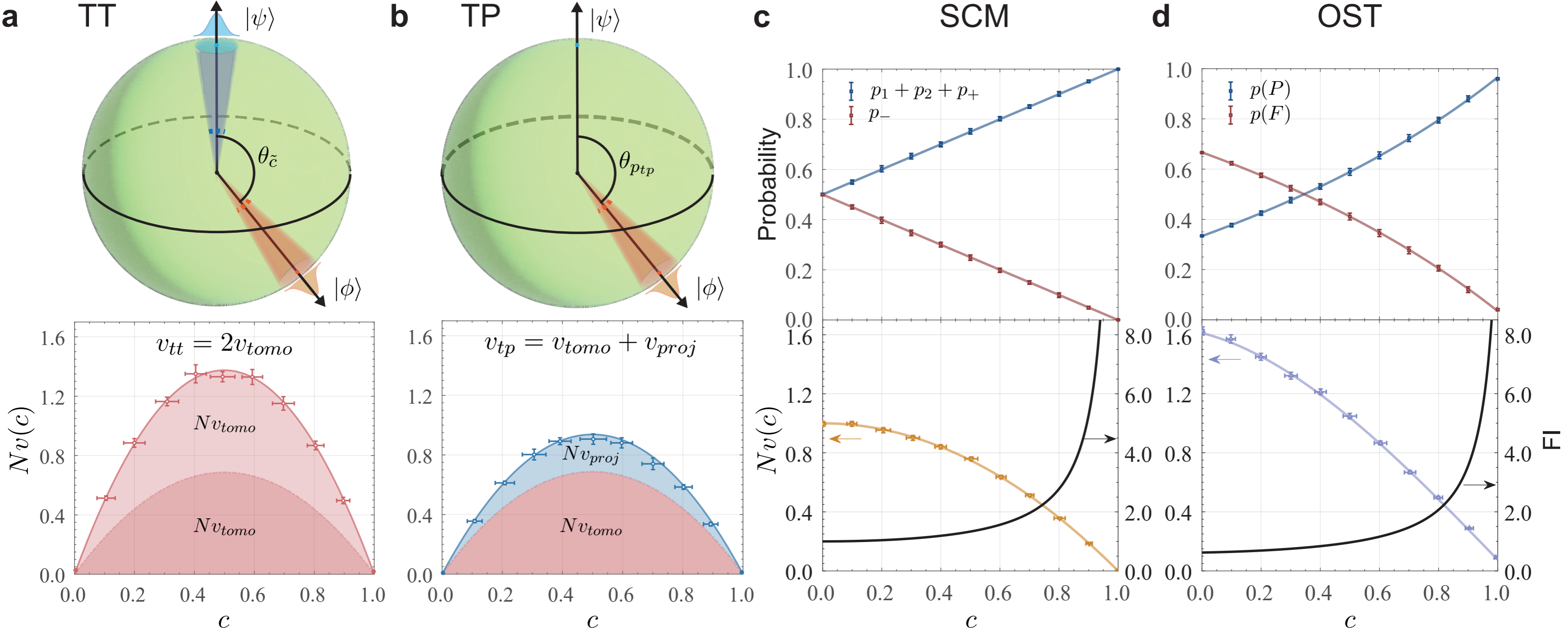

Supplement: Supplementary file 2 — Source files of figures in the main text [file 41377_2025_1755_MOESM2_ESM.zip › Figures_Main_Text/Fig3_Analysis.pdf]

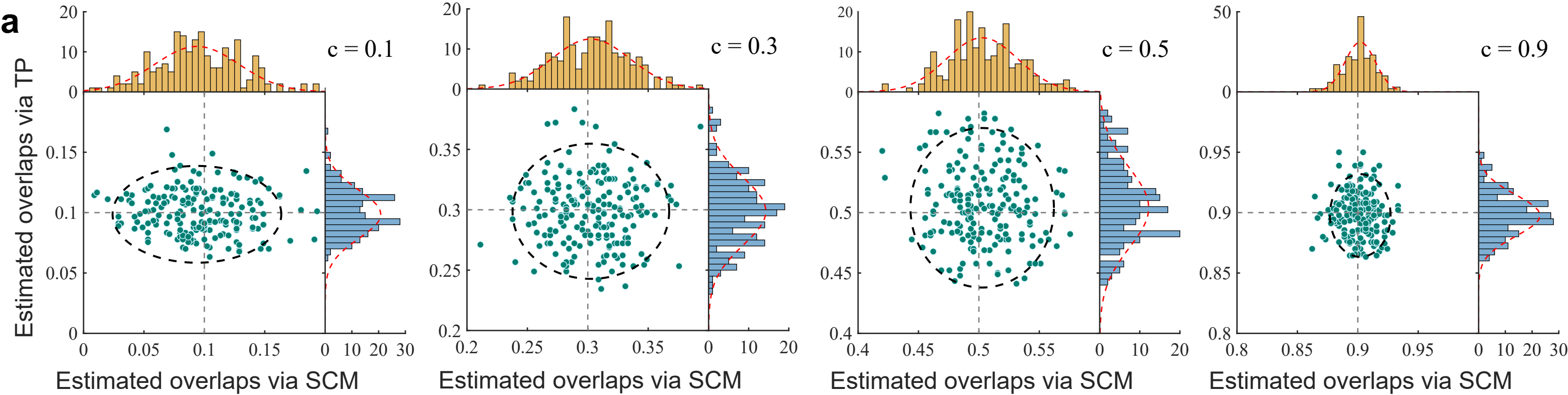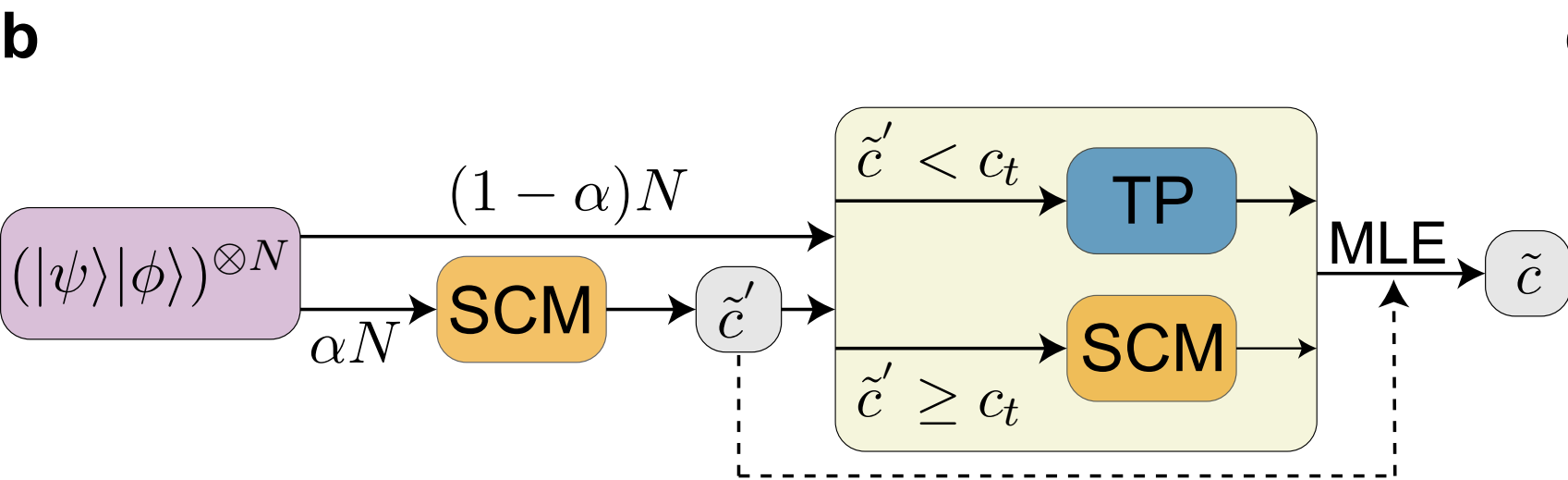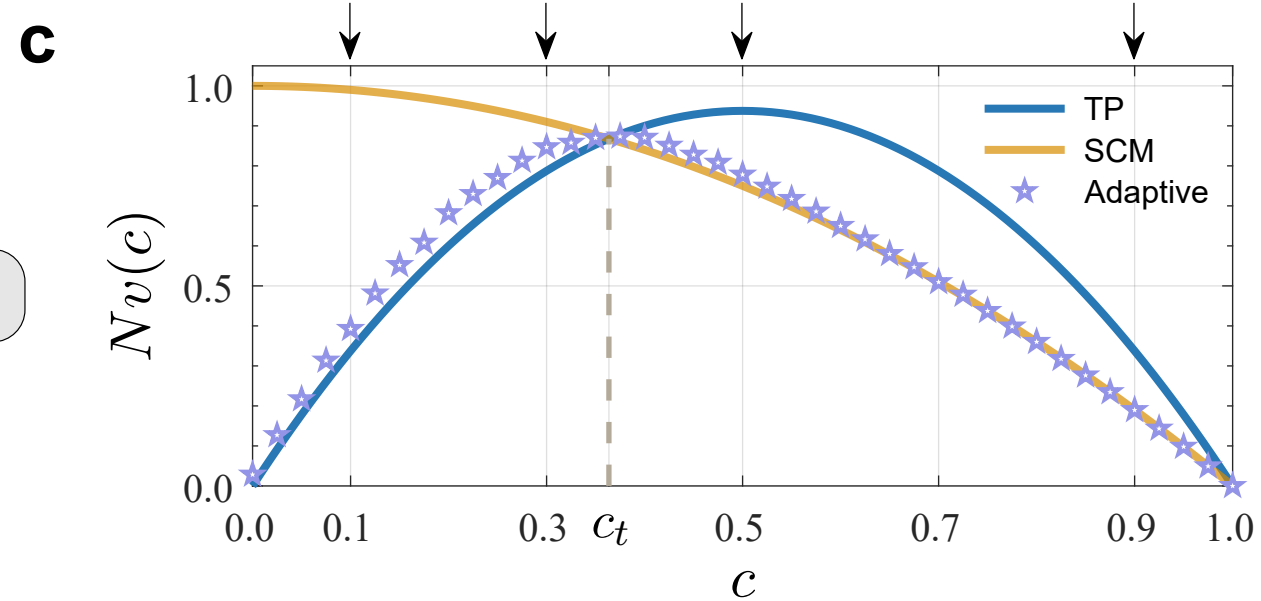

Supplement: Supplementary file 2 — Source files of figures in the main text [file 41377_2025_1755_MOESM2_ESM.zip › Figures_Main_Text/Fig4_Comparison_Adaptive.pdf]

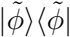

Supplement: Supplementary file 2 — Source files of figures in the main text [file 41377_2025_1755_MOESM2_ESM.zip › Figures_Main_Text/links/Fig1_Schematic_Setup_LaTeX2AI_1c68e74339172945.pdf]

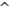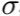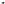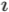

Supplement: Supplementary file 2 — Source files of figures in the main text [file 41377_2025_1755_MOESM2_ESM.zip › Figures_Main_Text/links/Fig1_Schematic_Setup_LaTeX2AI_4c0e0ae59ca3ac72.pdf]

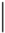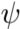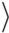

Supplement: Supplementary file 2 — Source files of figures in the main text [file 41377_2025_1755_MOESM2_ESM.zip › Figures_Main_Text/links/Fig1_Schematic_Setup_LaTeX2AI_539d24efe0f6ffa3.pdf]

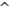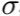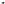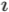

Supplement: Supplementary file 2 — Source files of figures in the main text [file 41377_2025_1755_MOESM2_ESM.zip › Figures_Main_Text/links/Fig1_Schematic_Setup_LaTeX2AI_5ee99512302b6115.pdf]

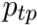

Supplement: Supplementary file 2 — Source files of figures in the main text [file 41377_2025_1755_MOESM2_ESM.zip › Figures_Main_Text/links/Fig1_Schematic_Setup_LaTeX2AI_68d11dae762b1165.pdf]

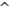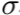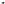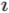

Supplement: Supplementary file 2 — Source files of figures in the main text [file 41377_2025_1755_MOESM2_ESM.zip › Figures_Main_Text/links/Fig1_Schematic_Setup_LaTeX2AI_708cb27a6dbc4d6c.pdf]

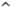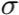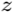

Supplement: Supplementary file 2 — Source files of figures in the main text [file 41377_2025_1755_MOESM2_ESM.zip › Figures_Main_Text/links/Fig1_Schematic_Setup_LaTeX2AI_888b1d940e4b34ee.pdf]

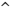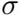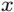

Supplement: Supplementary file 2 — Source files of figures in the main text [file 41377_2025_1755_MOESM2_ESM.zip › Figures_Main_Text/links/Fig1_Schematic_Setup_LaTeX2AI_a76e730ba744aba9.pdf]

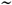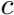

Supplement: Supplementary file 2 — Source files of figures in the main text [file 41377_2025_1755_MOESM2_ESM.zip › Figures_Main_Text/links/Fig1_Schematic_Setup_LaTeX2AI_a7931a2fe39735cd.pdf]

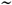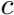

Supplement: Supplementary file 2 — Source files of figures in the main text [file 41377_2025_1755_MOESM2_ESM.zip › Figures_Main_Text/links/Fig1_Schematic_Setup_LaTeX2AI_aa2334ba31cb8fd0.pdf]

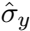

Supplement: Supplementary file 2 — Source files of figures in the main text [file 41377_2025_1755_MOESM2_ESM.zip › Figures_Main_Text/links/Fig1_Schematic_Setup_LaTeX2AI_aae3af8cda48afa2.pdf]

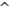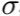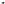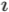

Supplement: Supplementary file 2 — Source files of figures in the main text [file 41377_2025_1755_MOESM2_ESM.zip › Figures_Main_Text/links/Fig1_Schematic_Setup_LaTeX2AI_abdc4cda519d52.pdf]

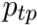

Supplement: Supplementary file 2 — Source files of figures in the main text [file 41377_2025_1755_MOESM2_ESM.zip › Figures_Main_Text/links/Fig1_Schematic_Setup_LaTeX2AI_b06e370952d1ad8a.pdf]

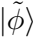

Supplement: Supplementary file 2 — Source files of figures in the main text [file 41377_2025_1755_MOESM2_ESM.zip › Figures_Main_Text/links/Fig1_Schematic_Setup_LaTeX2AI_b3779e0d5da3753b.pdf]

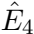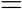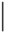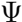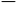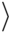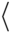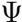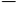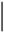

Supplement: Supplementary file 2 — Source files of figures in the main text [file 41377_2025_1755_MOESM2_ESM.zip › Figures_Main_Text/links/Fig1_Schematic_Setup_LaTeX2AI_d0ebc660a1cbc84d.pdf]

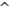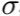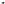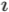

Supplement: Supplementary file 2 — Source files of figures in the main text [file 41377_2025_1755_MOESM2_ESM.zip › Figures_Main_Text/links/Fig1_Schematic_Setup_LaTeX2AI_ddd8814181807e45.pdf]

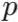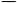

Supplement: Supplementary file 2 — Source files of figures in the main text [file 41377_2025_1755_MOESM2_ESM.zip › Figures_Main_Text/links/Fig1_Schematic_Setup_LaTeX2AI_e4180fedfdca9350.pdf]

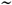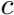

Supplement: Supplementary file 2 — Source files of figures in the main text [file 41377_2025_1755_MOESM2_ESM.zip › Figures_Main_Text/links/Fig1_Schematic_Setup_LaTeX2AI_e6882e60e8cfec67.pdf]

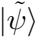

Supplement: Supplementary file 2 — Source files of figures in the main text [file 41377_2025_1755_MOESM2_ESM.zip › Figures_Main_Text/links/Fig1_Schematic_Setup_LaTeX2AI_e9e98469f4c4815f.pdf]

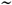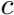

Supplement: Supplementary file 2 — Source files of figures in the main text [file 41377_2025_1755_MOESM2_ESM.zip › Figures_Main_Text/links/Fig1_Schematic_Setup_LaTeX2AI_eca94225b8d27e1d.pdf]

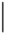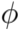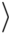

Supplement: Supplementary file 2 — Source files of figures in the main text [file 41377_2025_1755_MOESM2_ESM.zip › Figures_Main_Text/links/Fig1_Schematic_Setup_LaTeX2AI_f5abf3bce9b795e8.pdf]

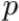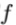

Supplement: Supplementary file 2 — Source files of figures in the main text [file 41377_2025_1755_MOESM2_ESM.zip › Figures_Main_Text/links/Fig1_Schematic_Setup_LaTeX2AI_feb70db6e24286f9.pdf]

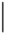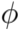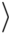

Supplement: Supplementary file 2 — Source files of figures in the main text [file 41377_2025_1755_MOESM2_ESM.zip › Figures_Main_Text/links/Fig3_Analysis_LaTeX2AI_304e4fb3f7d0f908.pdf]

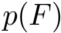

Supplement: Supplementary file 2 — Source files of figures in the main text [file 41377_2025_1755_MOESM2_ESM.zip › Figures_Main_Text/links/Fig3_Analysis_LaTeX2AI_403adc4e99f4f14a.pdf]

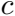

Supplement: Supplementary file 2 — Source files of figures in the main text [file 41377_2025_1755_MOESM2_ESM.zip › Figures_Main_Text/links/Fig3_Analysis_LaTeX2AI_4738eda1c506123c.pdf]

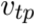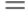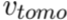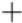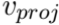

Supplement: Supplementary file 2 — Source files of figures in the main text [file 41377_2025_1755_MOESM2_ESM.zip › Figures_Main_Text/links/Fig3_Analysis_LaTeX2AI_49cc4b7dd1a8cc21.pdf]

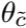

Supplement: Supplementary file 2 — Source files of figures in the main text [file 41377_2025_1755_MOESM2_ESM.zip › Figures_Main_Text/links/Fig3_Analysis_LaTeX2AI_5ec6736ff4075d6b.pdf]

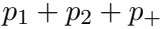

Supplement: Supplementary file 2 — Source files of figures in the main text [file 41377_2025_1755_MOESM2_ESM.zip › Figures_Main_Text/links/Fig3_Analysis_LaTeX2AI_624abe2e063efe5a.pdf]

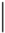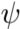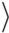

Supplement: Supplementary file 2 — Source files of figures in the main text [file 41377_2025_1755_MOESM2_ESM.zip › Figures_Main_Text/links/Fig3_Analysis_LaTeX2AI_6bfb9d43f814670e.pdf]

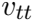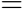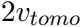

Supplement: Supplementary file 2 — Source files of figures in the main text [file 41377_2025_1755_MOESM2_ESM.zip › Figures_Main_Text/links/Fig3_Analysis_LaTeX2AI_98f7af27bc150f9f.pdf]

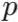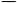

Supplement: Supplementary file 2 — Source files of figures in the main text [file 41377_2025_1755_MOESM2_ESM.zip › Figures_Main_Text/links/Fig3_Analysis_LaTeX2AI_c6924728c95afc69.pdf]

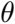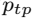

Supplement: Supplementary file 2 — Source files of figures in the main text [file 41377_2025_1755_MOESM2_ESM.zip › Figures_Main_Text/links/Fig3_Analysis_LaTeX2AI_f22e8f780aecf53f.pdf]

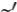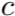

Supplement: Supplementary file 2 — Source files of figures in the main text [file 41377_2025_1755_MOESM2_ESM.zip › Figures_Main_Text/links/Fig4_Comparison_Adaptive_LaTeX2AI_2933385fdcb62859.pdf]

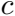

Supplement: Supplementary file 2 — Source files of figures in the main text [file 41377_2025_1755_MOESM2_ESM.zip › Figures_Main_Text/links/Fig4_Comparison_Adaptive_LaTeX2AI_33f989e8b29bb6d.pdf]

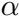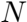

Supplement: Supplementary file 2 — Source files of figures in the main text [file 41377_2025_1755_MOESM2_ESM.zip › Figures_Main_Text/links/Fig4_Comparison_Adaptive_LaTeX2AI_4d8930464eaae7c6.pdf]

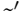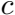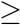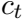

Supplement: Supplementary file 2 — Source files of figures in the main text [file 41377_2025_1755_MOESM2_ESM.zip › Figures_Main_Text/links/Fig4_Comparison_Adaptive_LaTeX2AI_717c334fadd93c7d.pdf]

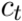

Supplement: Supplementary file 2 — Source files of figures in the main text [file 41377_2025_1755_MOESM2_ESM.zip › Figures_Main_Text/links/Fig4_Comparison_Adaptive_LaTeX2AI_7ab41a59cddbfab6.pdf]

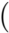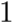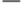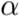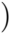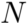

Supplement: Supplementary file 2 — Source files of figures in the main text [file 41377_2025_1755_MOESM2_ESM.zip › Figures_Main_Text/links/Fig4_Comparison_Adaptive_LaTeX2AI_84dbf895aa97f4d2.pdf]

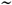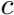

Supplement: Supplementary file 2 — Source files of figures in the main text [file 41377_2025_1755_MOESM2_ESM.zip › Figures_Main_Text/links/Fig4_Comparison_Adaptive_LaTeX2AI_a7b31869762dfd64.pdf]

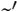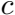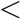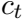

Supplement: Supplementary file 2 — Source files of figures in the main text [file 41377_2025_1755_MOESM2_ESM.zip › Figures_Main_Text/links/Fig4_Comparison_Adaptive_LaTeX2AI_fd1f1b7df40d3aab.pdf]
